# Ethnomedicinal survey of various communities residing in Garo Hills of Durgapur, Bangladesh

**DOI:** 10.1186/s13002-015-0033-3

**Published:** 2015-05-30

**Authors:** Md. Arif Khan, Md. Khirul Islam, Md. Afjalus Siraj, Sanjib Saha, Apurba Kumar Barman, Khalijah Awang, Md. Mustafizur Rahman, Jamil A. Shilpi, Rownak Jahan, Erena Islam, Mohammed Rahmatullah

**Affiliations:** Department of Biotechnology and Genetic Engineering, Mawlana Bhasani Science and Technology University, Santosh-1902, Bangladesh; Pharmacy Discipline, Life Science School, Khulna University, Khulna, Bangladesh; Department of Chemistry, Faculty of Science, University of Malaya, Kuala Lumpur, 50603 Malaysia; Centre for Natural Products and Drug, University of Malaya, Kuala Lumpur, 50603 Malaysia; Department of Biotechnology & Genetic Engineering, University of Development Alternative, Dhanmondi, Dhaka 1209 Bangladesh; Department of Pharmacy, University of Development Alternative, Dhanmondi, Dhaka 1209 Bangladesh

**Keywords:** Garo hills, Tribal people, Use value, Informant consensus factor, Fidelity level

## Abstract

**Background:**

Garo Hills represents one of earliest human habitation in Bangladesh preserving its ancient cultures due to the geographic location. It is situated in the most northern part of Durgapur sub-district having border with Meghalaya of India. Durgapur is rich in ethnic diversity with Garo and Hajong as the major ethnic groups along with Bangalee settlers from the mainstream population. Thus the ethnomedicinal practice in Garo Hills is considered rich as it encompasses three different groups. Present survey was undertaken to compile the medicinal plant usage among the various communities of the Garo Hills.

**Methods:**

The ethnomedicinal data was collected through open and focussed group discussions, and personal interviews using semi-structured questionnaire. A total of 185 people were interviewed, including the three community people and their traditional health practitioners (THPs). The usage of the plants were further analysed and are presented as use value (*UV*), informant consensus factor (*ICF*) and fidelity level (*FL*).

**Results:**

A total of 71 plants from 46 families and 64 genera were documented during our survey. Gastrointestinal disorders represented the major ailment category with the use of 36 plant species followed by dermatological problems (25 species). The *ICF* ranged from 0.90 to 0.99, with an average value of 0.96. Leaves (41) were the principle source of medication followed by fruits (27). Trees (33) were the major plant type used in the ethnobotanical practice. A total of 25 plants showed high *FL* (70.91 to 100 %) with 12 plants showing maximum *FL* (100 %). A number of the plants appear to have unique ethnomedicinal uses.

**Conclusion:**

Present investigation revealed a rich traditional practice in the studied region, which provides primary health care to the local community. This compilation of the ethnobotanical knowledge can help researchers to identify the uses of various medicinal plants that have a long history of use.

## Background

The Garo Hills in Durgapur sub-district is one of the most remote areas of the northern part of Bangladesh. Ethnic groups like the Garos and Hajongs reside in this area from ancient times along with Bangalee settlers who have also settled in this region hundreds of years ago. The Garos are one of the eminent ethnic groups of the Indian sub-continent. Around half a million Garo population can be found at various parts of the world, but most of them live in the north-eastern part of India [1]. At present, one-fifth of the total Garo population live in different regions of Bangladesh with their habitat spread in north-central districts namely Mymensingh, Netrakona, Gazipur, Sherpur and Tangail. Garos are mainly known for their matrilineal culture and individual kinship system [2]. Garos prefer to call themselves Achik (Hilly Garos) and Mandis (Plain Land Garos), although people not familiar with their culture simply call them as Garo [3]. Hajongs are also a small indigenous community of the Garo Hills. They came from Tibet to Assam and then to Bangladesh [4]. The Bangalee settlers, who although belonging to the mainstream population of Bangladesh, have settled alongside the ethnic communities and have through cross-cultural exchanges with the ethnic communities for centuries, have to some extent adopted the cultures of the ethnic communities.

Tribal people and people who live in remote areas depend considerably on medicinal plants for their primary treatment. Due to this factor, medicinal plant usage in such communities is far richer than say, the urban population. The exploration of the therapeutic activity of medicinal plants rendered by them has a long history of use passed on from generation to generation [5]. However, in recent years, there has been a continuous decline in their traditional medicinal practice due to several reasons including scarcity of medicinal plants due to mass deforestation, easier access to modern medicines, and reduced interest of younger generation towards herbal medicine. The objective of the present study was to document the medicinal plant knowledge prevailing in the Garo, Hajong and Bangalee communities residing in Garo Hills and compare the presently obtained information with previously reported ethnomedicinal uses of the plants in Bangladesh towards obtaining fresh insights into newer ethnobotanical uses of the plants. In our present study, the ethnopharmacological knowledge was collected from knowledgeable people belonging to the Garo, Hajong and Bangalee communities and the traditional health practitioners of the three communities. The results were further analyzed for comparative evaluation of the usage of individual plant species to provide an overview of medicinal plant usage in communities living in Garo Hills.

## Materials and methods

### Study area

The Garo tribal people can be found in districts north of Dhaak district in Bangladesh. These districts are Tangail, Mymensingh, Gazipur and Netrakona. They speak six dialects of the Mandi language, which are A’tong, Abeng, Brak, Chibok, Dual, and Megam. The Garos can also be found in the the adjacent bordering areas of India like Meghalaya. Most of the Garos are poor and their main occupation is agriculture or agricultutal labourers. In recent years, they are converting in mass numbers to Christianity. The Garos call themselves A-chik Mande, literally meaning the hill people.

The Hajongs are also a tribal community living alongside the Garos and can be found in districts like Mymensingh, Sherpur, Sylhet and Netrakona districts in Bangladesh, and Meghalaya in India. They have apparently come to their present region several hundred years ago. The Hajongs are basically a farming community, and by religion close to the Hindus. The Hajongs have their own language but do not have any alphabets. Some hajongs are lately converting to Christianity. In economic terms, like the Garos, most hajongs are poor and their literacy rate is very low.

The Bangalees belong to the mainstream population of Bangladesh. They have settled in the present region of survey along side the Garos and Hajongs from as early as 50 to 100 or more years. Their interaction with the Garos and Hajongs has largely been peaceful. Like the Garos and the Hajongs, the Bangalee community is also engaged in farming, and are mostly poor and illiterate.

**Fig. 1:**
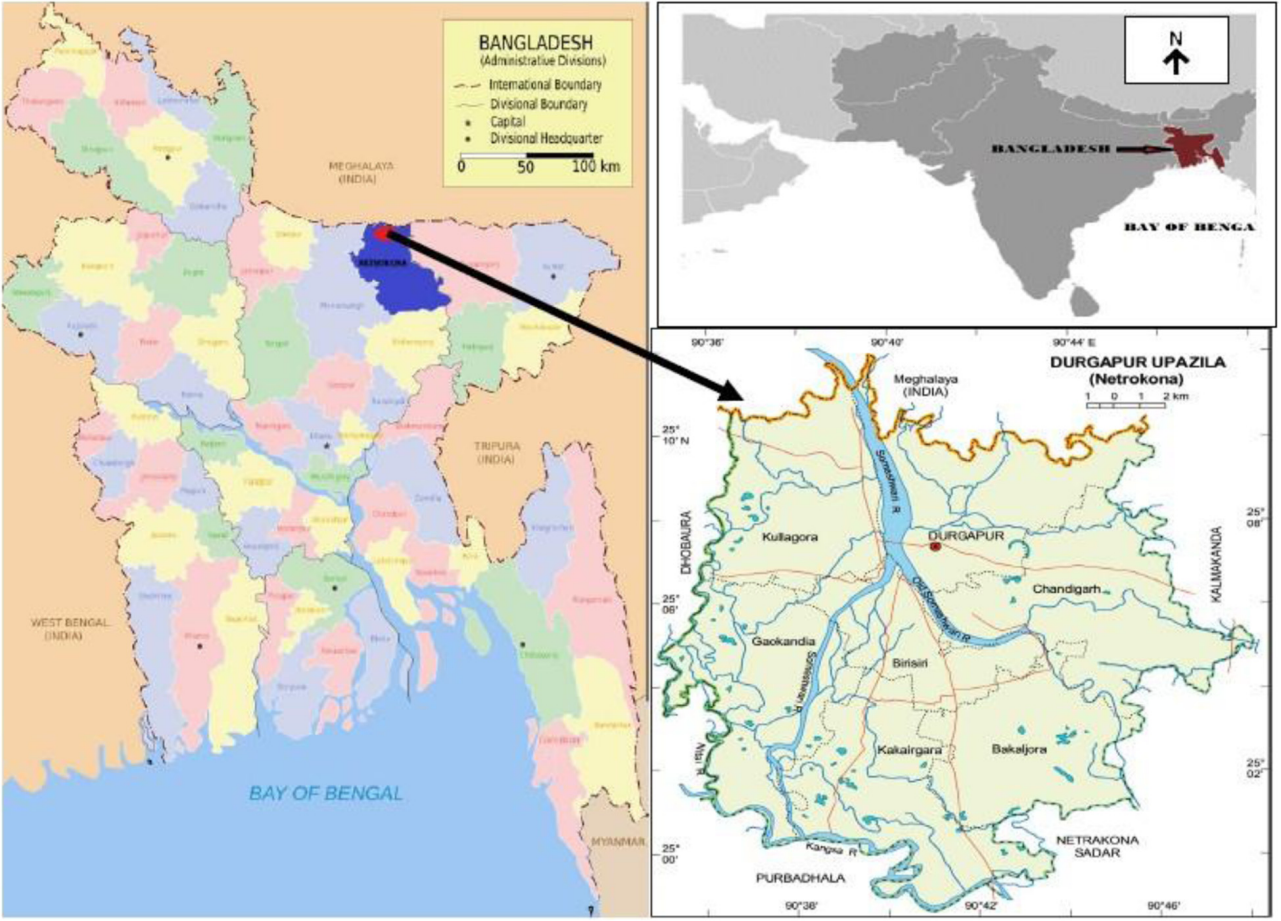
Geographical location of Durgapur Garo Hills area, Bangladesh

The survey was carried out at Garo hills, Durgapur sub-district which is under the district of Netrakona in Dhaka division, Bangladesh (Fig. 1). The area of Durgapur is 293.43 sq km. The study area is located in the most northern part of Durgapur, having the coordinates of 25.1250 °N and 90.6875 °E. Durgapur is surrounded by Meghalaya state of India on the north, Purbadhala and Netrakona Sadar on the south, Kalmakanda on the east, and Dhobaura sub-district on the west. The main rivers of this sub-district are Old Someshwari, Kangsa and Someshwari. The Garo valleys and hills are situated in the northern part of this sub-district. The Garo villages in Durgapur where the survey was conducted were Noluapara, Gupalpur, Bhobanipur, Badambari, Farongpara, Dahapara, and Fulbari. The Hajong villages were Gupalpur, Bhobanipur, Badambari, Shamnogor, and Baromari. The villages where the Bangalee communities resided and which were included in the survey were Atrakhali, Noluapara, Baromari, Fanda, and Cholk Lengura. It may be noted that the village of Noluapara contained both Garo and Bangalee communities, while the village of Badambari contained both Garo and Hajong communities, and the village of Baromari contained both Hajong and Bangalee communities. Thus to some extent, the three communities co-resided in the same village, and all the villages fell within Durgapur sub-district and thus were close to or adjacent to each other. As a result, there was a large amount of cross-cultural relationships between the three communities.

Government establishments are the only health facilities provided for inhabitants of Durgapur which include one health complex, one health center and seven family planning centers. However, such establishments lack adequate facilities and trained medical professionals. Geologically all part of the study area is almost identical. Topographically, the study area is characterized by its large hillocks, known as *tilla*. The soil pH fluctuates from 6 to 6.5. This area is located in the semi-drier part of Bangladesh. The highest temperature reaches to 30 °C during May and coldest to around 10 °C during January. The most common ethnic group of this area is Garo and Hajong with Bangalee community settlers interspersed within the two communities. So far, a total of 2924 Garo households and 505 Hajong households have been recorded in this sub-district; the number of Bangalee households have been recorded as 4778 [6].

### Ethnobotanical survey

The survey was conducted during the period from 10^th^ October, 2013 to 7^th^ May, 2014. Before starting the survey, general information was collected about the study area as well as THPs and general people. The data was collected following the standard guidelines of ethnobotanical data collection [7, 8]. A total of 185 people participated in interviews including THPs and knowledgeable people from all three communities. All were permanent residents of the study area. The highest number of respondents of our study belonged to the Garo and Hajong ethnic communities. Respondents were selected on the basis of whether they gave an affirmative answer when asked about their medicinal plant knowledge. Following an affirmative answer, detailed interviews were conducted with the respondents where they discussed their knowledge on medicinal plants and showed the plants. Multiple interviews (occasionally as many as 4–5) were conducted with the informants to gather as much detailed knowledge of the medicinal plants as possible.

### Data collection

While some people were co-operative to our initiative, some were less interested to continue with the conversation. In most cases, the preliminary hesitation was recovered through a brief explanation of the objective of our study in the native language of the informants. As the conversation continued, we tried to build the confidence of the interviewee so the person interacted spontaneously. Senior citizens, forest office and local administrations were consulted to identify personnel with sufficient knowledge in local ethnobotanical practices. During the study period, the ethnomedicinal data was collected through the open-ended, semi-structured interviews according to Martin and Cotton [9, 10]. The information of each plant was documented along with the local name, nature of the plant, plant part used, medicinal uses, mode of preparation, routes of administration, and degree of scarcity in this area. Recorded demographic data include the gender, age, ethnicity, experiences, and educational background. All information in relevance to data collection was carefully noted down. The informants were also requested for a walk to introduce us with the plants. Necessary plant parts were collected for the purpose of identification. Pictures of the plant/plant part were also taken to assist in the identification. Plant specimens were verified as to taxonomy at the Bangladesh National Herbarium. Voucher specimens were deposited with the Medicinal Plant Collection Wing of the University of Development Alternative (MPCW-UODA) and accession numbers obtained from there. For ethical issues connected to field work permission was obtained from various bodies like the Ethical Committee and the Institutional Review Board of the University of Development Alternative, and local Government bodies dealing with indigenous/tribal people. A clear understanding was made with the people surveyed that their intellectual property rights as to the information supplied will not be violated if the results lead to any economic benefits for us.

### Quantitative analysis

The medicinal uses of various plant species were listed in alphabetical order of the scientific names of the plants along with their family name, local name, nature of the plant, ethnomedicinal applications, mode of preparation, and route of administration. The results were presented and analysed further on the basis of their use and ailment categories. The diversity of the uses of medicinal plants were evaluated by calculating use value (*UV*), informant consensus factor (*ICF*), and fidelity level (*FL*).

Use values are computed for each plant to provide a quantitative measure of its comparative significance to the informants [11]. *UV* was calculated by the equation:

$$ U{V}_s={\varSigma}_iU{V}_{is}/{n}_s $$

where, ‘*UV*_*s*_’ indicates the use value of a particular species, ‘*UV*_*is*_’ is the number of use reports mentioned by the informants for that particular plant species and ‘*n*_*s*_’ is the total number of informants participated in our study. The main objective of *UV* calculation is to find out the degree of ethnomedicinal use for a particular plant species. High *UV* value indicates the broad acceptance of that particular plant species for a particular therapeutic use.

Informant consensus factor (*ICF*) was calculated to measure the homogeneity of the information for a specific plant to cure a specific ailment [12, 13]. The lowest and highest values of *ICF* can be 0.00 and 1.00, respectively. *ICF* was calculated using the equation:

$$ ICF=\left({N}_{ur}-{N}_t\right)/\left({N}_{ur}-1\right) $$

Where, ‘*N*_*ur*_’ refers to the total number of use reports for a particular ailment category, and ‘*N*_*t*_’ is the total number of species used for this ailment category. Several diseases were sorted out into a broad ailment category depending on similarity for the ease of the distribution of the plants.

Fidelity level (*FL*) expresses the priority of a species over the others in the management of a particular ailment and was calculated using the following formula:

$$ FL={I}_p/{I}_u\times 100 $$

Where, ‘*I*_*p*_’ is the number of informants stating the use of a species for a particular ailment category while ‘*I*_*u*_’ is the number of informants stating the use of that plant for any sort of ailment category. Higher *FL* value indicates more frequent of use of a given plant species for treating a particular ailment category by the informants.

## Results and discussion

### Demographic characteristics of participants in the study

The Garo tribe is one of the leading indigenous communities of Bangladesh which claims to be distributed among approximately 45 clans. The last population estimation of Garos was in 1991, when it was numbered at 68,210. While known to all as Garos, they refer to themselves as Aa’chik or Mandi. Anthropologists accept as true that they are a Tibetan-Burmese sub-group of the Mongoloid race which possesses language, shared history and culture. The Garos are divided into four sects, namely, Chatchi, Marakh, Momin, and Sangma. Their society is matriarchal with daughters inheriting their mother’s belongings. Garo’s novel religion used to be Shangsharik, but recently about 99 % of the Garos have converted to Christianity and belong to a variety of denominations like Baptists, Presbyterians, Protestants, Roman Catholics and Seventh Day Adventists.

Scholars do not have a clear opinion on the Hajong’s history, even the Hajong themselves. Some say the Hajong originated in the Hill Tracts of Chittagong, Bangladesh and afterward migrated to the northern areas of Bangladesh and into India. Another important opinion is that the Hajong tribe came from Tibet as descendents of the Kachhari people and settled in the Kamrup area of Assam state for several years. The northern Dhaka division, which constitutes the land of the Hajong in Bangladesh, consists of districts like Netrakona, Mymensingh, Sherpur and Jamalpur. Today, the Hajong of the Durgapur area continue to live primarily in Hajong villages together with Bengalis (Bangalee or Bengali/Bangla-speaking mainstream population) and other tribal communities, such as Garo and Koch, whose village homes are simple but clean. Women perform the family cooking in a separate attached hut adjacent to the living house, cooking rice as their staple food. Hajong are mainly a farming community and some work as hired day laborers in the fields, whereas others farm their own land. Some Hajong of Durgapur areas are also involved in collecting and selling wood from the Garo hills along the border. In terms of religion, Hajongs are close to Hindus. Hajongs worship Durga as well as other Hindu gods and goddesses. But Shiva is their principal deity. Hajongs wear ‘paita’ (a thread) on their bodies similar to Hindu Brahmans. Hajongs are believers in reincarnation too, like the Hindus.

The Bangalee community belongs to the mainstream population of Bangladesh, who speak Bengali or Bangla. While some families claim to have settled in the general areas of the Garos and Hajongs for more than a hundred years, others claim to have settled in the area in comparatively recent times like about 50 years ago. But in general, the Bangalee community lives in close association with the Garos and the Hajongs and the cultural practices of the three communities have intertwined in some aspects, the major difference being that most Bangalee settlers belong to the Muslim religion.

The survey was conducted among all three communities. The participants consisted of 62.16 % male and 37.83 % female. Out of 185 informants, 30 were THPs, 60 Bangalee people and the remaining 95 were tribal people from the Garo and Hajong communities (that is a total of 155 persons were non-traditional health practitioners but claimed to have good knowledge on medicinal plants). Prior Informed Consent was first obtained from all informants. According to the age, most of the informants (42.16 %) were 50–60 years old followed by informants (25.41 %) who were 40–50 years old (Table 1). Among 30 traditional health practitioners, 50 % had 10–20 years of experience in ethnomedicinal practice in their present area. According to the educational background, majority of the informants were illiterate (42.62 %), with only two persons holding post-graduate degrees. Among the participants, it was observed that as a general rule, THPs do not disclose the plant name or the formula of their preparation to other people (however, they provided this information to us after proper explanation as described before). They transfer their knowledge verbally either to one or more of their family members or to their assistant (known as the ‘Sishya’ of the THPs). On the other hand, non-THPs always reveal their knowledge to their family member or anyone from their community who is interested in such knowledge.

**Table 1:** Demographic data of the informants

Variable	Categories	No. of person	% Of informants
Gender	Male	115	62.16 %
	Female	70	37.83 %
Age	20-30 year	20	10.81 %
	30-40 year	25	13.51 %
	40-50 year	47	25.41 %
	50-60 year	78	42.16 %
	More than 60 years old	15	8.11 %
Informant category	Non-THPs (Garo and Hajong)	95	51.35 %
	THPs	30	16.22 %
	Non-THPs (Bangalee)	60	32.43 %
Type of the non-THP ethnic communities	Garo	70	73.68 %
	Hajong	25	26.32 %
Experience of the THPs	Less than 2 year	2	6.67 %
	2-5 year	3	10 %
	6-9 year	4	13.33 %
	10-20 year	15	50 %
	More than 20 year	6	20 %
Educational background	Illiterate	77	41.62 %
	Completed 5 years’ education	35	18.92 %
	Completed 8 years’ education	23	12.43 %
	Completed 10 years’ education	20	10.81 %
	Completed 12 years’ education	18	9.73 %
	Graduate (16 years’ education)	10	5.41 %
	Post-graduate (18 years’ education)	2	1.08 %

### Medicinal plants recorded

Through this survey, a total of 71 plant species belonging to 46 families were recorded, that have various medicinal uses in the study area. The highest number of medicinal plants belong to Fabaceae (6 species), followed by Rutaceae (4 species), Anacardiaceae (3 species), Asteraceae (3 species), Combretaceae (3 species), Myrtaceae (3 species) and Zingiberaceae (3 species). The results are shown in Table 2.

**Table 2:** Distribution of medicinal plant species of Durgapur Garo Hills according to their family

Family	No. of species	Family	No. of species
Fabaceae	6	Dilleniaceae	1
Rutaceae	4	Ebenaceae	1
Anacardiaceae	3	Euphorbiaceae	1
Asteraceae	3	Leguminosae	1
Combretaceae	3	Lythraceae	1
Myrtaceae	3	Malvaceae	1
Zingiberaceae	3	Meliaceae	1
Acanthaceae	2	Moringaceae	1
Amaranthaceae	2	Musaceae	1
Arecaceae	2	Oleaceae	1
Convolvulaceae	2	Oxalidaceae	1
Lamiaceae	2	Pedaliaceae	1
Lauraceae	2	Piperaceae	1
Moraceae	2	Poaceae	1
Apiaceae	1	Rhamnaceae	1
Asparagaceae	1	Rubiaceae	1
Araceae	1	Scrophulariaceae	1
Bombacaceae	1	Solanaceae	1
Brassicaceae	1	Sterculiaceae	1
Bromeliaceae	1	Thymelaeaceae	1
Caricaceae	1	Verbenaceae	1
Crassulaceae	1	Vitaceae	1
Cucurbitaceae	1	Xanthorrhoeaceae	1

Botanical families including Fabaceae, Rutaceae, Anacardiaceae, Asteraceae, Combretaceae, and Zingiberaceae have previously been shown to be the major medicinal plant families of Durgapur Garo Hills [6]. Similar results were found in surveys carried out in the Eastern Himalayan region of India [14]. The family Fabaceae reportedly has the highest number of species, more than any other plant family in the world [15, 16].

In the current study, high *UV*s were observed for *Ananas sativus* (1.96), *Aegle marmelos* (1.88), *Terminalia arjuna* (1.85), *Zingiber officinale* (1.75), *Bombax ceiba* (1.72) *Terminalia bellirica* (1.71), *Ocimum tenuiflorum* (1.71), *Carica papaya* (1.65), and *Adhatoda vasica* (1.65) indicating their wide usage in the ethnomedicinal practices in the study area. Multipurpose use of above plants might have contributed towards their high *UV*s [1].

The lowest *UV*s were obtained for *Cassia fistula* (0.21), *Lannea coromandelica* (0.24), *Cinnamomum verum* (0.28), *Ipomoea aquatica* (0.29), and *Acacia catechu* (0.33) (Tables 3, 4 and 5). However, a low *UV* does not nullify the merit of the medicinal value of a plant species as the low *UV* might be linked to low availability of the plant in the study area.

**Table 3:** List of medicinal plants of the Durgapur Garo Hills reported by THPs and local knowledgeable people of three communities

Serial No.	Scientific name and accession number	Family	Local name	Plant type	Parts used	Ailments	No. of ailment categories	Use value
01	*Abroma augusta* (L.) L.f. MPCW-UODA 1219/2014	Sterculiaceae	Ulot-kombol	W,T	Leaf, bark, root	Juice of the leaf and root for treatment of diabetes. Decoction of bark for female sexual disorders. Sap juice to treat menstrual disorders [O].	2	1.55
02	*Acacia catechu* (L. f.) Willd. MPCW-UODA 1220/2014	Fabaceae	Khoyer	W,T	Bark, wood	Decoction of bark for diarrhea [O]. Wood extract for skin diseases [T].	2	0.33
03	*Achyranthes aspera* L. MPCW-UODA 1221/2014	Amaranthaceae	Ubuth nangra	W,H	Root	Root is tied to head to treat headache [T]. Root paste for hemorrhage, eczema, diarrhea and constipation [O].	4	1.12
04	*Adhatoda vasica* Nees, Syn: *Justicia adhatoda* L. MPCW-UODA 1223/2014	Acanthaceae	Bashok/Adabaush	W,H	Whole plant, leaf	Decoction of whole plant for intestinal disorders. Leaf juice to treat pneumonia and cough [O]. Decoction of leaf for scabies and other skin diseases [T].	3	1.65
05	*Aegle marmelos* (L.) Corrêa ex Roxb. MPCW-UODA 1225/2014	Rutaceae	Bel	W,T	Leaf, fruit	Ripe and unripe fruits are eaten for constipation. Decoction of leaves for peptic ulcer. Leaf oil to treat respiratory disorders [O].	2	1.88
06	*Aloe barbadensis* Mill. MPCW-UODA 1226/2014	Xanthorrhoeaceae	Ghrita-kumari	W,H	Leaf, Latex	Leaf for skin dandruff and burns [T]. Leaf juice for stomach disorders [O]. Leaf-latex for constipation [O].	2	1.32
07	*Amaranthus spinosus* L. MPCW-UODA 1230/2014	Amaranthaceae	katakhuduira	W,H	Leaf, Stem	Hot extraction for the treatment of rheumatic pain, pain in the bones, blood or pus coming out with urine [O].	2	0.56
08	*Ananas sativus* Schult.f. MPCW-UODA 1231/2014	Bromeliaceae	Anarosh	C,H	Leaf, Fruit	Fruit juice is taken for fever. Young leaf is chewed for helminthiasis and jaundice [O].	3	1.96
09	*Andrographis paniculata* Nees MPCW-UODA 1232/2014	Acanthaceae	Kalomegh	W,H	Whole plant	Juice of whole plant for the treatment of fever, boil, ulcer and to enhance appeal for food [O].	3	1.16
10	*Anthocephalus chinensis* Hassk. MPCW-UODA 1233/2014	Rubiaceae	Kodom	W,T	Leaf, Bark	Leaf and bark decoction for ulcers, wounds, pain, constipation, and edema [O].	4	0.66
11	*Aquilaria malaccensis* Lamk., Syn: *Aquilaria agallocha* Roxb. MPCW-UODA 1234/2014	Thymelaeaceae	Agar	W,T	Wood	Wood extract used for body pain, and skin diseases [T]. Wood decoction for ulcer, edema, and jaundice [O].	5	0.41
12	*Asparagus racemosus* Willd. MPCW-UODA 1235/2014	Asparagaceae	Shotomuli	W,H	Leaf, Root	Decoction of leaf for epilepsy and stomach ulcers. Root extract with milk for physical weakness in male [O].	3	1.27
13	*Averrhoa carambola* L. MPCW-UODA 1236/2014	Oxalidaceae	Kamranga	W,T	Leaf, Fruit	Ripe fruits are eaten for cough, fever, appetite stimulant and jaundice [O]. Leaf juice for ringworm and chickenpox [T].	6	1.18
14	*Azadirachta indica* A. Juss. MPCW-UODA 1238/2014	Meliaceae	Neem	W,T	Leaf	Leaves are dried and powdered and taken every morning for allergy, eczema, skin diseases and diabetes [O].	2	1.52
15	*Bombax ceiba* L. MPCW-UODA 1240/2014	Bombacaceae	Shimul	W,T	Root, seed	Root juice of young plants for sexual disorders. Decoction of root to arouse sexual desire. Seed oil for gonorrhoea [O].	2	1.72
16	*Brassica oleracea* L. MPCW-UODA 1242/2014	Brassicaceae	Patakopi	C,H	Leaf	Cooked or salad to treat tonic, gynecological disorders [O].	2	0.67
17	*Calendula officinalis* L. MPCW-UODA 1243/2014	Asteraceae	Gada ful	G,H	Leaf, Flower	Mashed leaf and flower to treat old wound, menstrual problems and itches [T]. Flowers to treat stomach upset, ulcers and inflammation [O].	4	0.88
18	*Carica papaya* L. MPCW-UODA 1245/2014	Caricaceae	Pepe/Pabda	C,S	Leaf, Latex, Fruit	Ripe and unripe fruits are eaten to treat dysentery, diabetes, constipation and chronic indigestion. Latex for the treatment of peptic ulcer. Leaf paste for ringworm [O].	3	1.65
19	*Cassia alata* L*.* Syn: *Senna alata* (L.) Roxb. MPCW-UODA 1246/2014	Fabaceae	Daudraj	W,T	Leaf	Leaf paste is applied to treat skin disease, and scabies [T].	1	0.73
20	*Cassia fistula* L. MPCW-UODA 1247/2014	Fabaceae	Sonalu	W,T	Leaf, Fruit, Root	Ripe fruits to treat helminthiasis. Juice of leaf and root for long-term cough, nervous weakness [O].	3	0.48
21	*Cassia occidentalis* L. MPCW-UODA 1249/2014	Fabaceae	Jhi jhi gach	W,H	Root	Root paste is massaged on the leg with mustard oil for leg pain [T].	1	0.21
22	*Centella asiatica* (L.) Urb. MPCW-UODA 1250/2014	Apiaceae	Thankuni	W, H, G	Leaf	Leaf juice for dysentery [O]. Leaf paste applied on wounds, burns, and skin lesion [T].	2	1.04
23	*Cinnamomum tamala* (Buch.-Ham.) T.Nees & C.H.Eberm MPCW-UODA 1252/2014	Lauraceae	Tejpata	W,T	Leaf	Leaf paste to treat headache and pimples [T].	2	0.62
24	*Cinnamomum verum* J. Presl. MPCW-UODA 1251/2014	Lauraceae	Daruchini	W, T	Bark	Bark for asthma and coughs [O].	1	0.28
25	*Cissus quadrangularis* L. MPCW-UODA 1255/2014	Vitaceae	Harjora	W,H	Leaf, Stem, rhizome	Stem and rhizome paste for pain and bone fracture [T]. Dried leaf and stem for stomach upset, stomach ulcer, and malaria fever [O].	4	0.75
26	*Citrus acida* Pers*.* MPCW-UODA 1257/2014	Rutaceae	Lebu	G,S	Fruit	Fruit juice for facial scars and spots [T]. Fruits increase appetite [O].	2	1.55
27	*Citrus grandis* Hassk. MPCW-UODA 1258/2014	Rutaceae	Jambura	C,T	Fruit	Fruit juice for the treatment of fever [O], scabies, eczema, and itches [T].	2	1.47
28	*Clerodendrum viscosum* Vent. MPCW-UODA 1259/2014	Verbenaceae	Bhati	W,H	Leaf, Fruit	Juice from crushed leaf and fruit for helminthiasis and dysentery [O].	2	0.93
29	*Coccinia cordifolia* (L.) Cogn. MPCW-UODA 1261/2014	Cucurbitaceae	Telakucha	W,H	Whole plant, Leaf	Juice is produced from whole plant to treat diabetes, gonorrhea and constipation [O]. Leaf for wounds [T].	4	1.20
30	*Cocos nucifera* L. MPCW-UODA 1262/2014	Arecaceae	Narikel	C,T	Fruit	The inner portion of unripe fruit is used to treat skin disease and to remove skin spots [T]. Coconut water for diarrhea [O].	2	0.58
31	*Colocasia esculenta* (L.) Schott MPCW-UODA 1265/2014	Araceae	Kochu	C,H	Stem	Stem paste to stop bleeding from cuts and wounds [T].	1	1.02
32	*Curcuma longa* L. MPCW-UODA 1267/2014	Zingiberaceae	Holud	C,H	Rhizome	Rhizome juice for diarrhea and flatulence [O]. Rhizome paste to remove face spots [T].	2	0.63
33	*Cuscuta reflexa* Roxb. MPCW-UODA 1268/2014	Convolvulaceae	Shorno lota	W,H	Whole plant	Juice obtained from macerated whole plant for the treatment of jaundice [O].	1	0.89
34	*Cynodon dactylon* (L.) Pers. MPCW-UODA 1269/2014	Poaceae	Durba gash	W,H	Whole plant, Rhizome	Macerated whole plant is applied to stop bleeding from cuts and wounds [T]. Rhizome for heart failure. Whole plant extract for diabetes [O].	3	1.45
35	*Datura metel* L. MPCW-UODA 1270/2014	Solanaceae	Dhutura	W,H	Leaf, Flower, Seed	Flower and seed for cold and nervous disorders [O]. Crushed leaf is applied to painful areas [T].	2	0.51
36	*Delonix regia* (Bojer) Raf. MPCW-UODA 1271/2014	Fabaceae	Krishnochura	W,T	Leaf, Fruit	Fruit and leaf paste for piles and boils [O].	2	0.38
37	*Dillenia indica* L. MPCW-UODA 1273/2014	Dilleniaceae	Chalta	W,T	Fruit	Fruit juice is taken for fever and cough [O].	2	0.88
38	*Diospyros peregrina* Gürke MPCW-UODA 1275/2014	Ebenaceae	Gab	W,T	Bark, Fruit	Bark decoction to treat dysentery and cholera [O].	1	0.55
39	*Elettaria cardamomum* (L.) Maton MPCW-UODA 1276/2014	Zingiberaceae	Elach	W,T	Fruit, seed	Fruit and seed for asthma and coughs [O].	1	0.92
40	*Emblica officinalis* Gaertn. MPCW-UODA 1277/2014	Euphorbiaceae	Amloki	W,T	Fruit	Fruits for allergy, as food, and for gastric [O].	2	1.62
41	*Enydra fluctuans* Lour. MPCW-UODA 1278/2014	Asteraceae	Helencha	C,H	Whole plant,	Fried whole plant for intestinal disorders [O].	1	0.44
42	*Feronia limonia* Swingle Syn: *Limonia acidissima* Houtt. MPCW-UODA 1279/2014	Rutaceae	Kadbael	W,T	Fruit	Ripe fruits are eaten for flatulence, and pimple [O].	2	0.55
43	*Ficus racemosa* L. MPCW-UODA 1280/2014	Moraceae	Jog dumur	W,S	Leaf, Fruit	Leaf paste to arouse sexual desire and biliary disorders. Fruits are taken for treatment of diabetes [O].	3	0.44
44	*Hibiscus rosa-sinensis* L. MPCW-UODA 1284/2014	Malvaceae	Jaba	G,S	Whole plant, Leaf	Whole plant for dysentery. Leaf juice is taken to treat debility [O].	2	0.67
45	*Hyptis suaveolens* (L.) Poit.MPCW-UODA 1285/2014	Lamiaceae	Tokma	W,H	Fruit	Fruits for the treatment of flatulence, acidity, gastric troubles [O].	1	1.24
46	*Ipomoea aquatic* Forssk.MPCW-UODA 1287/2014	Convolvulaceae	Kalmi shak	W,H	Leaf	Fried leaf for constipation and piles [O].	2	0.29
47	*Kalanchoe pinnata* (Lam.) Pers. MPCW-UODA 1288/2014	Crassulaceae	Patharkuchi	W,H	Leaf, Root	Leaf juice to treat cholera, diarrhea, and dysentery [O].	1	0.37
48	*Lannea coromandelica* (Houtt.) Merr. MPCW-UODA 1289/2014	Anacardiaceae	Jiga	W,S	Leaf	Leaf paste for urinary problems, diabetes [O].	2	0.24
49	*Mangifera indica* L. MPCW-UODA 1290/2014	Anacardiaceae	Aam	C,T	Bark, flower, fruit	Ripe and unripe fruits used to treat dysentery [O]. Decoction of bark and flower to prevent graying of hair [T].	2	0.80
50	*Mikania cordata* (Burm.f.) B.L.Rob. MPCW-UODA 1292/2014	Asteraceae	Refugee lota/jarmani lota	W,H	Leaf	Macerated leaf to stop bleeding from external cuts and wounds [T].	1	0.93
51	*Mimosa pudica* L. MPCW-UODA 1293/2014	Leguminosae	Lajjaboti	W,H	Root	Decoction of roots for jaundice treatment [O].	1	0.66
52	*Moringa oleifera* Lam. MPCW-UODA 1295/2014	Moringaceae	Sojina	W,T	Leaf, Fruit	Leaf juice for fat control. Fruits for diabetes [O].	2	1.06
53	*Musa paradisiaca* L. MPCW-UODA 1296/2014	Musaceae	Bichi kola	C,S	Leaf, Fruit	Leaf juice for anemia. Unripe fruits to treat dysentery [O].	2	0.78
54	*Nyctanthes arbor-tristis* L. MPCW-UODA 1297/2014	Oleaceae	Sheuly	W,T	Leaf, Flower, Seed	Seed and flower paste for constipation. Leaf juice to treat fever [O].	2	0.41
55	*Ocimum tenuiflorum* L. MPCW-UODA 1298/2014	Lamiaceae	Tulsi	G,H	Leaf, Stem	Juice from macerated leaves to treat coughs [O]. Stems are worn as garland around the neck for tuberculosis [T].	2	1.71
56	*Phoenix sylvestris* (L.) Roxb. MPCW-UODA 1299/2014	Arecaceae	Khejur	W,V,T	Root, Fruit	Root juice for treatment of nervousness, cough and fever [O].	3	0.41
57	*Piper betel* Blanco MPCW-UODA 1301/2014	Piperaceae	Paan	W,V	Leaf	Juice from leaf for diabetes and acidity [O].	2	1.48
58	*Psidium guajava* L. MPCW-UODA 1302/2014	Myrtaceae	Peyara	G,S	Leaf	Juice from crushed leaves for piles, indigestion, diarrhea, dysentery and menstrual disorders [O].	3	0.86
59	*Punica granatum* L. MPCW-UODA 1303/2014	Lythraceae	Dalim	G,S	Fruit	Ripe fruit and leaf juice to treat diabetes, intestinal worms [O].	2	0.91
60	*Scoparia dulcis* L*.* MPCW-UODA 1304/2014	Scrophulariaceae	Dhonia	C,H	Leaf	Leaf is taken on an empty stomach for fever [O].	1	0.48
61	*Sesamum indicum* L. MPCW-UODA 1305/2014	Pedaliaceae	Tiil	C,S	Leaf, Seed	Leaf paste is used in fistula. Seed oil for the treatment of burns associated with infection, pain and blisters [T].	3	0.69
62	*Spondias pinnata* (L.f.) Kurz MPCW-UODA 1308/2014	Anacardiaceae	Amra	W,T	Bark, Fruit	Unripe fruits are eaten for dyspepsia [O]. Bark juice to treat dysentery [O].	1	0.64
63	*Streblus asper* Lour. MPCW-UODA 1310/2014	Moraceae	Sheora	W, T	Leaf, Root	Leaf paste for gastrointestinal disorders. Mashed root for increasing energy [O].	2	0.42
64	*Syzygium aromaticum* (L.) Merr. & L.M. Perry MPCW-UODA 1313/2014	Myrtaceae	Lobongo	W,T, C	Flower bud	Flower bud to treat asthma and coughs [O].	1	0.71
65	*Syzygium cumini* (L.) Skeels MPCW-UODA 1314/2014	Myrtaceae	Jaam	C,T	Leaf, Fruit	Decoction of leaf to induce vomiting. Ripe fruits to treat diabetes [O]. Bark juice is taken for excessive bleeding during menstruation and chronic dysentery [O].	3	1.45
66	*Tamarindus indica* L. MPCW-UODA 1315/2014	Fabaceae	Tetul	W,T	Leaf	Decoction of leaf for the treatment of sinusitis and chronic cold [O].	2	0.95
67	*Terminalia arjuna* (Roxb. ex DC.). Wight & Arn MPCW-UODA 1316/2014	Combretaceae	Arjun	W,T	Leaf, Bark	Soaked water of bark for the treatment of heart problem and burning sensations. Leaf juice to treat jaundice, dysentery [O].	3	1.85
68	*Terminalia bellirica* (Gaertn.) Roxb. MPCW-UODA 1317/2014	Combretaceae	Bohera	W,T	Fruit	Fruits are eaten for helminthiasis [O] and loss of hair [T].	2	1.71
69	*Terminalia chebula* Retz. MPCW-UODA 1319/2014	Combretaceae	Horitoki	W,T	Fruit	Fruits are soaked in water and then taken for constipation and vomiting [O].	1	1.01
70	*Zingiber officinale* Roscoe MPCW-UODA 1320/2014	Zingiberaceae	Ada	C,H	Rhizome	Rhizome is eaten for coughs, stomach pain and gastric [O].	3	1.75
71	*Ziziphus mauritiana* Lam. MPCW-UODA 1322/2014	Rhamnaceae	Boroi	W,T	Leaf, fruit	Leaf juice for dysentery and diarrhea. Ripe fruits are eaten for constipation [O].	1	1.28

T: Tree, H: Herb, S: Shrub, V: Vine, C: Cultivated, G: Grown in the garden, W: Wild; [O]: Oral, [T]: Topical

To calculate *ICF*s, all the recorded 82 ailments were grouped into 16 major ailment categories (Table 4) [35, 36]. The medicinal plants were distributed according to these major categories. *ICF*s were calculated for the pre-recorded plants and ranged from 0.90 to 0.99 (Table 5). High *ICF* value refers to an increased evidence of the efficacy of a plant species to treat a given ailment [37]. The ailment category of ‘sexual stimulant’ has the highest *ICF* scoring (0.99). *Bombax ceiba* (*UV* 1.72) is mainly used as sexual stimulant. Although the *ICF* for gastrointestinal disorders (0.96) was less compared to ailment categories of sexual stimulant, heart diseases, or hematological disorders, the highest number of plant usage (36 species) was documented under this category. Use of too many plant species for a wide range of ailments can render a low *ICF* and may require further analysis of the results to identify the most useful plant for a given ailment. On the other hand, a high *ICF* signifies that the users have greater reliability with the use of the plants for the respective major ailment category. There is a higher possibility of for plants with higher *ICF* to contain relevant bioactive phytochemicals [35]

**Table 4:** Ailments grouped by major ailment categories

Category	Common diseases/Medical terms	No. of species used
Gastrointestinal disorders	Constipation, peptic ulcer, stomach disorders, ulcer, appeal for food, stomach ulcer, dysentery, increase appetite, diarrhea, flatulence, intestinal disorders, cholera, acidity, dyspepsia, gastrointestinal disorders, vomiting, chronic dysentery, burning sensations, gastric troubles, chronic indigestion	36
Fever	Fever, malaria fever	09
Respiratory complaints	Pneumonia, cough, respiratory disorders, long-term cough, asthma, tuberculosis, chronic cold	13
Diabetes	Diabetes, fat control	11
Liver disorders	Jaundice, biliary disorders	07
Dermatological problems	Dandruff, burns, boil, wounds, skin diseases, allergy, eczema, itch, scabies, skin lesion, pimples, bone fracture, facial scars, spots, bleeding from cuts and wounds, chicken pox	25
Urinary and rectal diseases	Blood or pus coming out with urine, gonorrhea, urinary problems, fistula, piles, edema	09
Inflammation and pain	Headache, rheumatic pain, bone pain, body pain, leg pain, sinusitis, stomach pain	12
Hematological disorders	Anaemia, hemorrhage	02
Sexual stimulant	Arouse sexual desire, sexual disorders	02
Hair growth	Prevent graying of hair, loss of hair	02
General health	Tonic, increasing energy, physical weakness in male, debility	04
Nervous system	Epilepsy, nervous weakness	04
Helminthiasis	Helminthiasis, ringworm, intestinal worms	07
Female sexual disorders	Menstrual disorders, gynecological disorders, excessive bleeding during menstruation	05
Heart problems	Heart diseases	02

**Table 5:** Informant consensus factor (ICF) for categorized ailments

Category	No. of species	% Of all species	No. of use reports	% Of all use reports	Informant consensus factor (*ICF*)	Most frequently used plant species
Gastrointestinal disorders	36	24	860	25.73	0.96	*Aegle marmelos*
Fever	09	6	210	6.28	0.96	*Citrus grandis*
Respiratory complaints	13	8.67	237	7.09	0.95	*Ocimum tenuiflorum*
Diabetes	11	7.33	190	5.68	0.95	*Syzygium cumini*
Liver disorders	07	4.67	102	3.05	0.90	*Cuscuta reflexa*
Dermatological problems	25	16.67	620	18.55	0.96	*Azadirachta indica*
Urinary and rectal diseases	09	6	160	4.79	0.95	*Calendula officinalis*
Inflammation and pain	12	8	225	6.73	0.95	*Zingiber officinale*
Hematological disorders	02	1.33	55	1.65	0.98	*Musa paradisiaca*
Sexual stimulant	02	1.33	79	2.36	0.99	*Bombax ceiba*
Hair growth	02	1.33	37	1.11	0.97	*Terminalia belerica*
General health	04	2.67	114	3.41	0.97	*Asparagus racemosus*
Nervous system	04	2.67	65	1.94	0.97	*Cassia fistula*
Helminthiasis	07	4.67	150	4.49	0.96	*Ananas sativus*
Female sexual disorders	05	3.33	108	3.23	0.96	*Abroma augusta*
Heart problems	02	2.67	57	1.71	0.98	*Terminalia arjuna*
Total	150^a^		3343			

^**a**^Each species may be listed in several categories

The fidelity level (*FL*) of the plants, which were cited 25 times or more for any particular disease are listed in Table 6 with the lowest and highest *FL*s being 70.91 % and 100 %, respectively. The highest *FL* of 100 % was recorded for 12 plant species of which, three species namely, *Aegle marmelos*, *Carica papaya, Terminalia chebula* were used for gastrointestinal disorders. Thus, among 36 plant species used in gastrointestinal disorders, three were found to be used extensively. Among 25 plants used for dermatological problems, only *Azadirachta indica* scored 100 % *FL*.

**Table 6:** Fidelity level (FL) of medicinal plants cited for 25 or more times

Sl. no.	Medicinal plant	Ailment category	Lp^a^	Lu^b^	FL^c^(%)
01	*Abroma augusta*	Female sexual disorders	55	57	95.74
02	*Adhatoda vasica*	Respiratory complaints	48	53	90.57
03	*Aegle marmelos*	Gastrointestinal disorders	110	110	100.00
04	*Aloe barbadensis*	Gastrointestinal disorders	62	72	86.11
05	*Ananas sativus*	Helminthiasis	120	120	100.00
06	*Andrographis paniculata*	Gastrointestinal disorders	51	62	82.26
07	*Asparagus racemosus*	General health	46	61	75.40
08	*Averrhoa carambola*	Helminthiasis	86	86	100.00
09	*Azadirachta indica*	Dermatological problems	90	90	100.00
10	*Bombax ceiba*	Sexual stimulant	77	77	100.00
11	*Calendula officinalis*	Urinary and rectal diseases	37	48	77.08
12	*Carica papaya*	Gastrointestinal disorders	102	102	100.00
13	*Cassia fistula*	Nervous system	45	49	91.84
14	*Centella asiatica*	Gastrointestinal disorders	62	75	82.66
15	*Citrus grandis*	Fever	106	106	100.00
16	*Coccinia cordifolia*	Diabetes	55	61	90.16
17	*Cuscuta reflexa*	Liver disorders	51	67	76.11
18	*Cynodon dactylon*	Dermatological problems	48	59	81.36
19	*Emblica officinalis*	Gastrointestinal disorders	72	85	84.71
20	*Hyptis suaveolens*	Gastrointestinal disorders	48	59	81.36
21	*Musa paradisiacal*	Hematological disorders	34	47	72.34
22	*Ocimum tenuiflorum*	Respiratory complaints	105	105	100.00
23	*Piper betel*	Diabetes	39	55	70.91
24	*Syzygium cumini*	Diabetes	92	92	100.00
25	*Terminalia arjuna*	Heart diseases	97	97	100.00
26	*Terminalia bellirica*	Hair growth	55	65	84.62
27	*Terminalia chebula*	Gastrointestinal disorders	77	77	100.00
28	*Zingiber officinale*	Inflammation and pain	95	95	100.00
29	*Ziziphus mauritiana*	Gastrointestinal disorders	38	44	86.36

^**a**^The number of use reports cited for a given species for a particular ailment category

^**b**^The number of informants cited the species for any ailment category

^**c**^Fidelity level

Plants having high *FL* values in other ailment categories are *Ananas sativus, Averrhoa carambola*, *Bombax ceiba, Citrus grandis, Ocimum tenuiflorum, Syzygium cumini, Terminalia arjuna,* and *Zingiber officinale.* These plants are widely used in many ethnobotanical practices around the world with sufficient scientific validations of their ethnomedicinal use [16–18].

### Information regarding the preparation

**Fig. 2:**
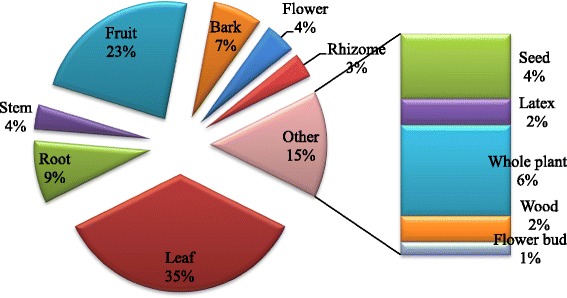
Pi chart representing relative uses of various plant parts in traditional practice

Various plant parts including leaves, root, stem, fruits, bark, flowers, seeds, whole plant, and rhizomes were widely used for the treatment of diverse types of ailments. Leaves were found to be the most used plant part in the ethnomoedicinal practice of Garo Hills, which was followed by fruits (23 %), and root (9 %) (Fig. 2). Similar to our present finding, leaves were found to be the most used plant part in many other ethnomedicinal practices [19–23]. Metabolically the most active part of the plant, leaves are known to synthesize a wide range of secondary metabolites [24, 25]. Leaves are also the first choice in ethnomedicine due to the easy collection and preparation procedure [26, 27].

**Fig. 3:**
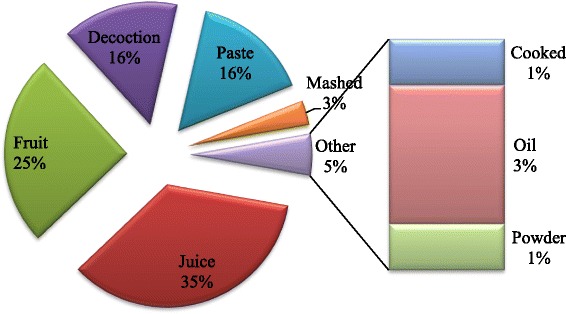
Pi chart of mode of preparation

**Fig. 4:**
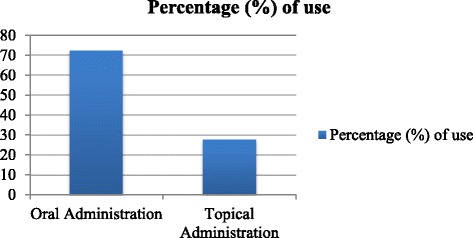
Mode of administration of ethnomedicinal preparation

There are several modes of preparation of ethnomedicines, including juice, decoction, powder, paste, oil, etc. The major mode of ethnobotanical preparations in Garo Hills was found to be juice (35 %), followed by fresh fruits (25 %), decoction (16 %), and paste (16 %) (Fig. 3). The local people of Garo Hills often add salt, sugar, banana, milk or lemon (e.g., see *Asparagus racemosus*) to enhance the effectiveness or palatability of a preparation. Paste is prepared using mortar and pestle, and then often mixing it with mustard oil, coconut oil or ginger (e.g., *Cassia occidentalis*). For THPs, it is more common to use more than one plant in the formulation of a preparation, to be used for the treatment of a particular ailment. A comparison of the mode of administration of the preparations is presented in Fig. 4, which has a similar trend as that of some other ethnobotanical reports [28].

### Habit, habitat, and nature of the plants

**Fig. 5:**
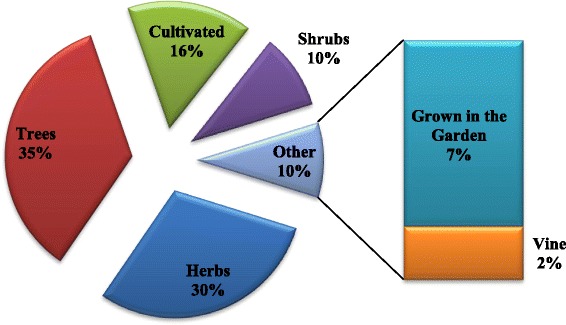
Type of plants used in the traditional medicine of Garo Hills

Among the recorded 71 medicinal plant species of Garo Hills, 35 % are trees followed by 30 % herbs, and 10 % shrubs (Fig. 5). While some plants are grown in home gardens, most of them can be found growing naturally in places including pond side, roadside, riverside or in the hills. Trees and herbs enjoy a higher usage in ethnomedicinal practice because of their greater availability [29, 30]. Of the recorded 71 plant species; THPs reported the highest number of plants species used by them as trees during our current study.

### Comparative ethnomedicinal uses of the plants in Bangladesh

A review of the comparative ethnomedicinal uses of the 71 medicinal plants by the Garo, Hajong and Bangalee communities in the present study versus previously reported ethnomedicinal uses of those plants in Bangladesh is shown in Table 7. Of the 71 plant species obtained in the present survey, 39 plant species appears to be quite extensively used by folk and tribal medicinal practitioners in other parts of Bangladesh as judged from the various ethnomedicinal uses of those plant species reported in the published literature. These plant species are *Abroma augusta*, *Achyranthes aspera*, *Adhatoda vasica*, *Aegle marmelos*, *Aloe barbadensis*, *Amaranthus spinosus*, *Andrographis paniculata*, *Asparagus racemosus*, *Azadirachta indica*, *Bombax ceiba*, *Carica papaya*, *Cassia alata*, *Centella asiatica*, *Cissus quadrangularis*, *Clerodendrum viscosum*, *Coccinia cordifolia*, *Colocasia esculenta*, *Curcuma longa*, *Cuscuta reflexa*, *Cynodon dactylon*, *Datura metel*, *Emblica officinalis*, *Hibiscus rosa-sinensis*, *Kalanchoe pinnata*, *Mangifera indica*, *Mikania cordata*, *Mimosa pudica*, *Moringa oleifera*, *Nyctanthes arbour-tristis*, *Ocimum tenuiflorum*, *Psidium guajava*, *Scoparia dulcis*, *Streblus asper*, *Syzygium cumini*, *Tamarindus indica*, *Terminalia arjuna*, *Terminalia bellirica*, *Terminalia chebula*, and *Zingiber officinale*. All of these plants have multiple uses, and the uses observed in the present survey match at least one of the reported uses.

**Table 7:** Other reported ethnomedicinal uses of the plants in Bangladesh

Plant name	Other reported ethnomedicinal uses in Bangladesh
*Abroma augusta* (L.) L.f.	Diabetes, sexual disorder by the Garo tribe living in Netrakona district [19]; weakness and headache by folk medicinal practitioners (FMPs) of two villages in Rajshahi district [38]; used against diabetes by FMPs of Vasu Bihar village, Bogra district [39]; used against spermatorrhea by a village FMP in Faridpur district [40]; spermatorrhea by FMPs of Sylhet Division, Bangladesh [41]; dyspepsia, dysentery, physical sickness, urinary incontinence, burning sensations in the urinary tract in Shitol Para village, Jhalokati district [42]; leucorrhea, scabies by FMPs of three villages in Natore and Rajshahi districts [43]; sex stimulant by FMPs of Daudkandi sub-district of Comilla district [44]; leucorrhea, menstrual problems by FMPs of Dinajpur district [45]; gonorrhea, leucorrhea, constipation, menstrual troubles by the Garo tribe inhabiting the Madhupur forest region of Bangladesh [46]; irregular menstruation, painful menstruation, burning sensations in the uterus by FMPs of Noakhali district [47]; debility by FMPs in villages by the Ghaghot River of Rangpur district [48]; debility by FMPs in villages by the Padma River of Rajshahi district [48]; diabetes by FMPs of Vasu Bihar village, Bogra district [49]; astringent by FMPs of Balidha village, Jessore district [50]; to induce regular menstruation, abscess, sexual diseases in men, low sperm count by FMPs of Station Purbo Para village, Jamalpur district [33]; menstrual problems, meho (endocrinological disorders, diabetes) by FMPs of Shetabganj village, Dinajpur district [32]; passing of semen with urine by FMPs of Daulatdia Ghat, Kushtia district [51]; debility, infertility in women due to problems in uterus (badhok disease) by FMPs of Vitbilia village in Pabna district [52]; to increase libido by FMPs of six villages in Greater Naogaon district [53]; gonorrhea, sexual weakness by FMPs of seven villages in Ishwardi Upazilla, Pabna district [54]; burning sensations during urination, dysentery, pain by FMPs of a village in Narayanganj district [55]; sexual disorders by a FMP of Gachabari village in Tangail district [56]; irregular menstruation by tribal medicinal practitioners (TMPs) of the Chakma tribe residing in Rangamati district [57]; meho (endocrinological disorders), promeho (sexually transmitted diseases like gonorrhea) by FMPs of three villages in Kurigram district [58]; burning sensations during urination, sexual problems by TMPs of Goala tribe of Moulvibazar district [59]; to keep body cool by a FMP of Savar in Dhaka district [60]; sexual weakness, general weakness by FMPs of four villages in Natore and Rajshahi districts [61]; ‘meho’ (endocrinological disorder but generally indicative of diabetes) by TMPs of the Tudu sub-clan of the Santal tribe in Joypurhat district [62]; sexual weakness, irregular menstruation, pain and burning sensations during menstruation by TMPs of 15 clans of the Garo tribe of Madhupur, Tangail district [63]; rise of blood pressure during night by FMPs of Bhola district [64]; irregular menstruation, leucorrhea, anemia by a FMP of Jhalokathi in Barisal district [65]; decrease in sexual strength by FMPs of two villages in Natore district [31]; headache, hardening of skins in the body by FMPs of Chuadanga district [66]; premature ejaculation, antidote to poisoning, intestinal dysfunction by the tribal healers of Oraon tribe of Sylhet district [67]; weakness by the TMPs of the Rai Clan of the Tipra tribe of Sylhet Division [68]; spermatorrhea by a FMP of a village in Faridpur district [40]; vaginitis by TMPs of two Marma tribal communities in two villages of Khagrachhari district [69]; low sperm count by TMPs and FMPs practicing within a Khasia tribal community in Jaflong area, Sylhet district [70]; diabetes, urinary disorder by a Pahan TMP in Dinajpur district [71]; diabetes by FMPs and TMPs in the vicinity of Lawachara Forest Reserve, Moulvibazar district [72]; physical weakness by TMPs of Kole and Rai tribes of Rajshahi and Nawabganj districts [73].
*Acacia catechu* (L. f.) Willd.	Blood dysentery in humans and cattle by FMPs of three areas in Pirojpur district [74].
*Achyranthes aspera* L.	Snake bite, diabetes, gonorrhoea by the Garo tribe living in Netrakona district [19]; menstrual disorders and burning sensations during urination in two villages of Rajshahi district [38];]; frequent ejaculation by itself in Faridpur district [40]; leucorrhea, dysentery, jaundice in Shitol Para village, Jhalokati district [42]; blood dysentery, toothache, wound, sciatica, abortifacient, eczema by FMPs of three villages in Natore and Rajshahi districts [43]; abortifacient by the Garo tribe inhabiting the Madhupur forest region of Bangladesh [46]; coughs, pneumonia, snake bite, eczema, stomach pain, gonorrhea, low semen count, leucorrhea by FMPs of Noakhali district [47]; hematuria by FMPs in villages by the Ghaghot River of Rangpur district [48]; to stop bed-wetting in children, tooth diseases, enlargement or shrinking of scrotum by FMPs in villages by the Padma River of Rajshahi district [48]; low sperm count, to increase sexual power, debility, jaundice by FMPs of Station Purbo Para village, Jamalpur district [33]; jaundice, to increase libido by FMPs of six villages in Greater Naogaon district [53]; eczema by FMPs of seven villages in Ishwardi Upazilla, Pabna district [54]; leucorrhea by a FMP of Gachabari village in Tangail district [56]; burning sensations during urination, kidney stone by FMPs of three villages in Kurigram district [58]; tooth infections, irregular menstruation by TMPs of Goala tribe of Moulvibazar district [59]; dermatitis, chronic dysentery, blood dysentery, menorrhagia by FMPs of four villages in Natore and Rajshahi districts [61]; leucorrhea by TMPs of 15 clans of the Garo tribe of Madhupur, Tangail district [63]; skin infections by FMPs of two villages in Natore district [31]; infertility in woman, bleeding while pregnant (blood can be present in urine or just comes out of the vagina) by FMPs of Chuadanga district [66]; low density of semen by FMP of Jhenidah district [66]; frequent ejaculation by itself by a FMP of a village in Faridpur district [40]; vomiting tendency, diarrhea, excessive blood during menstruation by TMPs and FMPs practicing within a Khasia tribal community in Jaflong area, Sylhet district [70]; injury by a Pahan TMP in Dinajpur district [71]; dental abscess, diabetes by TMPs of Kole and Rai tribes of Rajshahi and Nawabganj districts [73]; jaundice, respiratory problems by the Marma tribe living in Naikhongchhari, Bandarban district [75]; poisonous animal bites, inflammation of the body, fever, cough and mucus due to cold, asthma, tonsillitis by the Rakhain tribe inhabiting the Chittagong Hill Tracts region [76]; severe stomach pain, excessive bleeding following menstruation by the Santal tribe residing in Rajshahi district [77]; edema by the Tripura tribe residing in Chittagong Hill Tracts, Bangladesh [78]; to increase libido by FMPs of Badarganj and Shekhertek villages in Rangpur district [79]; bitter, to increase appetite, vomiting tendency, coughs, obesity, respiratory tract disorders, piles, pain, gastrointestinal disorders, jaundice by FMPs of three villages in Sreepur Upazilla, Magura district [80]; nocturnal emissions, constipation, burning sensations during urination by FMPs of Terbaria and Babla villages in Tangail district [81]; stomach pain, flatulency by TMPs of Bongshi tribe in Tangail district [82]; severe pain, whitish discharge from vagina, hair loss, jaundice, tooth infections, uterine problems by FMPs of several areas of Faridpur and Rajbari districts [83]; diabetes by TMPs of Naik clan of Rajbongshi tribe of Moulvibazar district [84]; to increase sexual strength by TMPs of Rai Kshatriya tribe of Pabna district [85]; urinary problems like passing of blood in urine by TMPs of the Pankho tribe of Bilaichari Union, Rangamati district [86]; abscess by TMPs of the Murmu tribal community residing in Rajshahi district [87]; bitter, to increase appetite, piles, respiratory tract disorders, pain, gastrointestinal disorders, to increase sperm, vomiting tendency, cough, obesity, jaundice by FMPs of two villages in Bagerhat district [88]; uterine inversion by TMPs of the Khatriya and Kashya clans of the Bagdi tribe in Rajbari district [89]; jaundice by TMPs of the Tripura tribe residing in Comilla district [90]; having trouble during urination, passing of blood during urination by a Tonchongya tribal healer of Rangamati district [91]; jaundice by a FMP practicing among tea garden workers in Sreemangal, Maulvibazar district [92]; asthma by a Garo TMP practicing among Garo and Kush tribes in Sherpur district [93]; rabies, stomach pain, cough, pneumonia by folk herbalists in Comilla district [94]; jaundice by TMPs of the Harbang clan of the Tripura tribe of Mirsharai area, Chittagong district [95]; male infertility, impotency by TMPs of the Nag clan of the Rai Ghatual tribe in Moulvibazar district [96].
*Adhatoda vasica* Nees	Cough, pneumonia, asthma by the Garo tribe living in Netrakona district [19]; respiratory problems by FMPs of Sylhet Division, Bangladesh [41]; coughs, asthma, bleeding from piles in Shitol Para village, Jhalokati district [42]; anthelmintic, cough, sedative, sprain by FMPs of three villages in Natore and Rajshahi districts [43]; cough by FMPs of Daudkandi sub-district of Comilla district [44]; coughs and pain in humans, any type of porcine diseases by FMPs of Dinajpur district [45]; pain, cold, asthma, wounds, cough, mucus by FMPs of Noakhali district [47]; rabies, pneumonia, jaundice by FMPs in villages by the Ghaghot River of Rangpur district [48]; antidote to poisoning, bronchitis, malaria, skin eruption, astringent in humans, cold in cattle by FMPs in villages by the Bangali River of Bogradistrict [48]; fever, cold, coughs by FMPs of Balidha village, Jessore district [50];cough, asthma, menstrual problems, jaundice, hepatitis B by FMPs of Station Purbo Para village, Jamalpur district [33]; asthma by FMPs of Shetabganj village, Dinajpur district [32]; leucorrhea, chronic respiratory disorders, coughs by FMPs of Daulatdia Ghat, Kushtia district [51]; whooping cough by FMPs of Vitbilia village in Pabna district [52]; severe fever with mucus by FMPs of six villages in Greater Naogaon district [53];chronic asthma, leprosy by FMPs of seven villages in Ishwardi Upazilla, Pabna district [54]; respiratory difficulties, asthma by FMPs of a village in Narayanganj district [55]; coughs and mucus by FMPs of three villages in Kurigram district [58]; cold, coughs, fever, ear lobe infection by FMPs of four villages in Natore and Rajshahi districts [61]; mucus, coughs by TMPs of 15 clans of the Garo tribe of Madhupur, Tangail district [63]; coughs, mucus, fever, tuberculosis, passing of blood through the mouth due to lung disorders by FMPs of Bhola district [64]; leprosy, allergy by a FMP of Jhalokathi in Barisal district [65]; fever, pneumonia, mucus, helminthiasis by FMPs of two villages in Natore district [31]; bone fracture, to increase appetite by FMPs of Chuadanga district [66]; coughs by FMP of Jhenidah district [66]; coughs by the tribal healers of Gor tribe of Sylhet district [67]; dry cough by the TMPs of the Rai Clan of the Tipra tribe of Sylhet Division [68]; tuberculosis by TMPs of two Marma tribal communities in two villages of Khagrachhari district [69]; coughs in children by TMPs and FMPs practicing within a Khasia tribal community in Jaflong area, Sylhet district [70]; coughs by FMPs of three areas in Pirojpur district [74]; helminthiasis, diarrhea and constipation by the Marma tribe living in Naikhongchhari, Bandarban district [75]; coughs, asthma by FMPs of Badarganj and Shekhertek villages in Rangpur district [79]; tuberculosis by FMPs of Terbaria and Babla villages in Tangail district [81]; coughs, biliary problems (bile turns the color of blood), frequent thirsts, respiratory problems, fever, vomiting tendency, diabetes, leprosy, tuberculosis by FMPs of three villages in Sreepur Upazilla, Magura district [80]; coughs by TMPs of Naik clan of Rajbongshi tribe of Moulvibazar district [84]; coughs by TMPs of the Pankho tribe of Bilaichari Union, Rangamati district [86]; flatulency, low sperm count, sperm incapable of being fertilized, astringent, bitter, to increase flow of bile, biliary problems like bile turning the color of blood, diarrhea, dysentery, tuberculosis, coughs, fever, asthma, eczema, leprosy by FMPs of two villages in Bagerhat district [88]; coughs, tuberculosis, toothache by TMPs of the Tripura tribe residing in Comilla district [90]; fever, coughs by a Kush tribal practitioner practicing among Garo and Kush tribes in Sherpur district; dry coughs by a Chakma tribal practitioner practicing among Garo and Kush tribes in Sherpur district; asthma by a Garo tribal practitioner practicing among Garo and Kush tribes in Sherpur district [93]; coughs, mucus by folk herbalists in Comilla district [94]; cough, mucus, asthma by TMPs of the Harbang clan of the Tripura tribe of Mirsharai area, Chittagong district [95]; fever, loss of appetite by TMPs of the Nag clan of the Rai Ghatual tribe in Moulvibazar district [96]; mucus by FMPs of Dhamrai sub-district, Dhaka district [97]; coughs by FMPs of Barisal Town, Barisal district [98]; malaria, coughs, colds by TMPs of Tonchongya tribe of Roangchaari Upazila in Bandarban district [99]; coughs, mucus by the Teli clan of the Telegu tribe of Maulvibazar district [100]; coughs by TMPs of Chakma tribe of Rangapanir Chara Area in Khagrachaari district [34]; diabetes, coughs by TMPs of the Manipuri tribe in Kamalganj Upazila, Moulvibazar district [101]; fever, coughs by TMPs of the Bauri tribal community of Moulvibazar district [102]; skin infections, tuberculosis by a TMP of the Deb barma clan of the Tripura tribe of Moulvibazar district [103].
*Aegle marmelos* (L.) Corrêa ex Roxb.	Constipation, dysentery, indigestion, pain by the Garo tribe living in Netrakona district [19]; constipation, dysentery, small size of penis in two villages of Rajshahi district [38]; dysentery in Vasu Bihar village, Bogra district [39]; urinary bladder stone by FMPs of Sylhet Division, Bangladesh [41]; pain under the umbilicus or stomach pain due to helminthic infestations, constipation, helminthiasis, decreased sperm count, aphrodisiac in Shitol Para village, Jhalokati district [42]; indigestion, cooling, appetizer, loss of libido, weakness, paralysis by FMPs of three villages in Natore and Rajshahi districts [43]; dysentery, peptic ulcer by FMPs of Daudkandi sub-district of Comilla district [44]; indigestion, loss of appetite, constipation, weakness, dysentery, snake bite, skin infections by FMPs of Noakhali district [47]; flatulence by FMPs in villages by the Ghaghot River of Rangpur district [48]; liver disorder, sunstroke, jaundice, constipation, sexual disorder, piles in humans, apepsia in cow or sheep by FMPs in villages by the Bangali River of Bogra district [48]; dysentery by FMPs of Vasu Bihar village, Bogra district [49]; chronic dysentery, diabetes by FMPs of Station Purbo Para village, Jamalpur district [33]; to remove foul odor of sweat in adults, vomiting in children by FMPs of Shetabganj village, Dinajpur district [32]; to remove odor from sweat, incoherency or insanity, acidity, ear and eye diseases by FMPs of Daulatdia Ghat, Kushtia district [51]; digestive aid by FMPs of six villages in Greater Naogaon district [53]; blood dysentery, constipation, to increase memory, to prevent stomach upsets by FMPs of a village in Narayanganj district [55]; gastrointestinal disorders like flatulence, constipation, stomach pain by tribal medicinal practitioners (TMPs) of the Chakma tribe residing in Rangamati district [57]; dysentery, to remove odor of sweat, vomiting in children by FMPs of three villages in Kurigram district [58]; acne by a FMP of Savar in Dhaka district [60]; chronic dysentery, dandruff by FMPs of four villages in Natore and Rajshahi districts [61]; dysentery, to keep stomach cool by FMPs of Bhola district [64]; blood dysentery by a FMP of Jhalokathi in Barisal district [65]; reduced sexual desire in humans by FMPs of Chuadanga district [66]; snake bite, stomach disorders by FMP from Jhenaidah district [66]; chronic dysentery, burning sensations in the body, heart palpitations by the TMPs of the Rai Clan of the Tipra tribe of Sylhet Division [68]; indigestion, loss of appetite by FMPs and TMPs in the vicinity of Lawachara Forest Reserve, Moulvibazar district [72]; dysentery by TMPs of two Marma tribal communities in two villages of Khagrachhari district [69]; to keep head cool, sprain, fracture by TMPs of Kole and Rai tribes of Rajshahi and Nawabganj districts [73]; chronic dysentery, constipation, indigestion by FMPs of three areas in Pirojpur district [74]; sedative by the Marma tribe living in Naikhongchhari, Bandarban district [75]; indigestion, piles, constipation, respiratory problem, inflammation, poisonous insect or snake bites, heart palpitations, fever, clearing of bowels by the Rakhain tribe inhabiting the Chittagong Hill Tracts region [76]; to keep body cool, diarrhoea, dysentery, constipation, astringent, repeat fevers, contagious fevers, frequent urination (diabetes) by the Tripura tribe residing in Chittagong Hill Tracts, Bangladesh [78]; acidity, skin allery, excessive sexual desire, carminative, coughs, astringent by FMPs of three villages in Sreepur Upazilla, Magura district [80]; severe pain, whitish discharge from vagina, hair loss, gastric problems by FMPs of several areas of Faridpur and Rajbari districts [83]; constipation by TMPs of Naik clan of Rajbongshi tribe of Moulvibazar district [84]; sexual disorder in males, to increase attraction in a female for a male by TMPs of Rai Kshatriya tribe of Pabna district [85]; rheumatism, insect repellent, flatulency by TMPs of the Soren clan of the Santal tribe in Rajshahi district [104]; jaundice, indigestion by TMPs of the Murmu tribal community residing in Rajshahi district [87]; to increase digestive capability, flatulence, coughs, to keep body cool, to clear stool by FMPs of two villages in Bagerhat district [88]; dysentery, diarrhea by TMPs of the Tripura tribe residing in Comilla district [90]; stomach pain, constipation, memory enhancer by folk herbalists in Comilla district [94]; jaundice, asthma by TMPs of the Bauri tribal community of Moulvibazar district [102]; stomach disorders, watery stool, loss of appetite by a TMP of the Deb barma clan of the Tripura tribe of Moulvibazar district [103]; diarrhea by a FMP of Sreemangal Upazila in Maulvibazar district [105]; spermatorrhea, insomnia by a FMP of Jamalpur district [106]; anti-inflammatory, constipation, blood dysentery, diabetes by TMPs of Santal tribe of Rangpur district [107].
*Aloe barbadensis* Mill.	Headache, hot feeling in head, and stomach disorders in two villages of Rajshahi district [38]; liver disorders and bloating in Faridpur district [40]; constipation, rheumatism, digestive aid by FMPs of Sylhet Division, Bangladesh [41]; burns due to fire, energizer, low semen density, to increase eyesight, spermatorrhea, constipation, to improve texture of skin in Shitol Para village, Jhalokati district [42]; ecbolic, irregular menstruation, wound, appetizer, blood dysentery by FMPs of three villages in Natore and Rajshahi districts [43]; purgative, piles by FMPs of Daudkandi sub-district of Comilla district [44]; leucorrhea by FMPs of Dinajpur district [45]; chronic constipation, diabetes, asthma, burning sensation during sexual ejaculation, to stimulate hair growth, carminative, respiratory problems by FMPs of Noakhali district [47]; debility by FMPs in villages by the Ghaghot River of Rangpur district [48]; to disinfect water by FMPs of Station Purbo Para village, Jamalpur district [33]; tuberculosis by FMPs of Shetabganj village, Dinajpur district [32]; to increase strength, increase semen, enlarged spleen, bloating, hepatic diseases, pain, boils by FMPs of Daulatdia Ghat, Kushtia district [51]; sexually transmitted diseases in men, skin disorders by FMPs of Vitbilia village in Pabna district [52]; severe headache by FMPs of seven villages in Ishwardi Upazilla, Pabna district [54]; constipation, burns, skin disorders, diabetes by FMPs of a village in Narayanganj district [55]; to increase libido, to improve texture of skin, blisters due to burns, tuberculosis by FMPs of three villages in Kurigram district [58]; constipation, hypertension, anxiety by FMPs of four villages in Natore and Rajshahi districts [61]; skin disorders, burning sensations in skin, hair loss, physical weakness, sexually transmitted diseases, leucorrhea by TMPs of 15 clans of the Garo tribe of Madhupur, Tangail district [63]; piles, stool clarification, physical weakness by the TMPs of the Rai Clan of the Tipra tribe of Sylhet Division [68]; liver disorders, bloating by a FMP of a village in Faridpur district [40]; low sperm count by TMPs of two Marma tribal communities in two villages of Khagrachhari district [69]; tendon pain by FMPs and TMPs in the vicinity of Lawachara Forest Reserve, Moulvibazar district [72]; weakness, tuberculosis, meho (endocrinological disorders, diabetes) by FMPs of three areas in Pirojpur district [74]; dysentery, blood dysentery by a Chakma TMP practicing among Garo and Kush tribes in Sherpur district; physical weakness by a Garo TMP practicing among Garo and Kush tribes in Sherpur district [93]; to keep head cool, dysentery by FMPs of Dhamrai sub-district, Dhaka district [97]; to keep body cool, burning sensations during urination by Christians living in Mirzapur village of Dinajpur ditrict, Bangladesh [108]; laxative, appetizer, alopecia, asthma, tuberculosis by FMPs of Boalia sub-district, Rajshahi district [109]; constipation by FMPs of Bheramara area in Kushtia district [110].
*Amaranthus spinosus* L.	Dysentery in two villages of Rajshahi district [38]; gastrointestinal disorders in Vasu Bihar village, Bogra district [39]; frequent ejaculation of sperm with blood in Faridpur district, Bangladesh [40]; to increase strength and to stimulate appetite by FMPs of Sylhet Division, Bangladesh [41]; leucorrhea, cough, dysentery by FMPs of three villages in Natore and Rajshahi districts [43]; to stop frequent urination by FMPs of Dinajpur district [45]; boils, abscesses by the Garo tribe inhabiting the Madhupur forest region of Bangladesh [46]; blood dysentery by FMPs in villages by the Ghaghot River of Rangpur district [48]; gastrointestinal disorders by FMPs of Vasu Bihar village, Bogra district [49]; debility, diabetes by FMPs of Station Purbo Para village, Jamalpur district [33]; stoppage of urination and defecation, diabetes, burning sensations during urination by FMPs of Shetabganj village, Dinajpur district [32]; diarrhoea, heat stroke, leucorrhea by FMPs of Vitbilia village in Pabna district [52]; hoarseness of voice, throat pain by FMPs of six villages in Greater Naogaon district [53]; stoppage of urination and defecation, toothache, bleeding from gums by FMPs of three villages in Kurigram district [58]; red color of urine by TMPs of Bongshi tribe in Tangail district [82]; waist pain by TMPs of Goala tribe of Moulvibazar district [59]; dysentery, sexual stimulant by FMPs of four villages in Natore and Rajshahi districts [61]; low semen density by the tribal healers of Oraon tribe of Sylhet district [67]; frequent ejaculation of sperm along with blood by a FMP of a village in Faridpur district [40]; flatulence, dysentery by FMPs and TMPs in the vicinity of Lawachara Forest Reserve, Moulvibazar district [72]; irregular whitish discharge in urine of women by TMPs of Kole and Rai tribes of Rajshahi and Nawabganj districts [73]; dysentery by FMPs of Badarganj and Shekhertek villages in Rangpur district [79]; jaundice by FMPs of Terbaria and Babla villages in Tangail district [81]; to regularize urine and stool, lack of appetite, bitter taste in all foods, blood purifier, antidote to poisoning by FMPs of three villages in Sreepur Upazilla, Magura district [80]; excessive bleeding during menstruation by TMPs of the Pankho tribe of Bilaichari Union, Rangamati district [86]; leucorrhea by FMPs of two villages in Bagerhat district [88]; jaundice by TMPs of the Tripura tribe residing in Comilla district [90]; physical weakness by a Garo tribal practitioner practicing among Garo and Kush tribes in Sherpur district [93]; stomach ache by TMPs of the Harbang clan of the Tripura tribe of Mirsharai area, Chittagong district [95]; diabetes in humans, to increase lactation in cows by FMPs of Dhamrai sub-district, Dhaka district [97]; rheumatic pain, pain in the bones, blood or pus coming out with urine by FMPs of Barisal Town, Barisal district [98]; bone fracture, eczema by TMPs of the Manipuri tribe in Kamalganj Upazila, Moulvibazar district [101]; low semen density by a FMP of Sreemangal Upazila in Maulvibazar district [105]; dysentery by Christians living in Mirzapur village of Dinajpur ditrict, Bangladesh [108]; gonorrhoea, laxative, expectorant by FMPs of Boalia sub-district, Rajshahi district [109]; to increase lactation in cows by the Santal tribe residing in Thakurgaon district [111]; urine coming out in small drops in breast-fed infants because of kidney stones arising from problems in mother’s milk by TMPs of the Hodi tribe in Sherpur district [112].
*Ananas sativus* Schult.f.	Fever, helminthiasis, jaundice by the Garo tribe living in Netrakona district [19]; helminthiasis, stomach pain by FMPs of Sylhet Division, Bangladesh [41]; jaundice, helminthiasis by FMPs of Noakhali district [47]; helminthiasis by FMPs of Shetabganj village, Dinajpur district [32]; wasting away of body in women by FMPs of six villages in Greater Naogaon district [53]; helminthiasis, fever, cold, coughs by FMPs of a village in Narayanganj district [55]; helminthiasis by FMPs of three villages in Kurigram district [58]; fever, hookworm infection by TMPs of 15 clans of the Garo tribe of Madhupur, Tangail district [63]; helminthiasis, to increase appetite, mucus by FMPs of Bhola district [64]; helminthiasis by the tribal healers of Gor tribe of Sylhet district [67]; pneumonia, asthma, respiratory problems by the Marma tribe living in Naikhongchhari, Bandarban district [75]; helminthiasis, jaundice by the Rakhain tribe inhabiting the Chittagong Hill Tracts region [76]; anthelmintic, antibacterial, urinary problem, stimulate appetite by the Tripura tribe residing in Chittagong Hill Tracts, Bangladesh [78].
*Andrographis paniculata* Nees	Fever and helminthiasis in Faridpur district, Bangladesh [40]; liver diseases, helminthiasis in Shitol Para village, Jhalokati district [42]; emetic, anthelmintic, sexual disorders by FMPs of three villages in Natore and Rajshahi districts [43]; long-term fever, any type of severe body pain by FMPs of Dinajpur district [45]; fever, headache, vertigo by the Garo tribe inhabiting the Madhupur forest region of Bangladesh [46]; liver diseases by FMPs of Feni district [47]; fever by FMPs in villages by the Padma River of Rajshahi district [48]; liver disorders, helminthiasis, acidity by FMPs of Vitbilia village in Pabna district [52]; stomach and heart disorders by TMPs of Goala tribe of Moulvibazar district [59]; jaundice, malaria, blood purifier, allergy by TMPs of 15 clans of the Garo tribe of Madhupur, Tangail district [63]; fever, pneumonia, mucus, helminthiasis by FMPs of two villages in Natore district [31]; allergy, chronic fever by FMP of Jhenidah district [66]; constipation, stomach pain by the TMPs of the Rai Clan of the Tipra tribe of Sylhet Division [68]; fever, helminthiasis by a FMP of a village in Faridpur district [40]; diabetes, stomach pain by a Pahan TMP in Dinajpur district [71]; coughs, cold by FMPs and TMPs in the vicinity of Lawachara Forest Reserve, Moulvibazar district [72]; helminthiasis, fever by TMPs of Kole and Rai tribes of Rajshahi and Nawabganj districts [73]; fever, constipation by TMPs of Bongshi tribe in Tangail district [82]; stomach disorders, to improve digestion, bloating with burning sensations in chest by FMPs of several areas of Faridpur and Rajbari districts [83]; common cold, uncomplicated sinusitis, pharyngotonsillitis, lower urinary tract infections, acute diarrhea, bacillary dysentery, bronchitis, carbuncles, colitis, cough, dyspepsia, fever, hepatitis, malaria, mouth ulcers, sores, tuberculosis, venomous snake bite, colic, otitis media, vaginitis, pelvic inflammatory disease, chicken pox, eczema, burns by FMPs of two villages in Bagerhat district [88]; cold, coughs, fever by TMPs of the Khatriya and Kashya clans of the Bagdi tribe in Rajbari district [89]; fever arising suddenly during the night, toothache, skin infections by TMPs of the Tripura tribe residing in Comilla district [90]; fever by a Chakma tribal practitioner practicing among Garo and Kush tribes in Sherpur district; rheumatic problems, gastric problems by a Kush tribal practitioner practicing among Garo and Kush tribes in Sherpur district [93]; fever, loss of appetite by TMPs of the Nag clan of the Rai Ghatual tribe in Moulvibazar district [96]; jaundice, helminthiasis by TMPs of Chakma tribe of Rangapanir Chara Area in Khagrachaari district [34]; fever, malarial fever by TMPs of the Bauri tribal community of Moulvibazar district [102]; malaria by a TMP of the Deb barma clan of the Tripura tribe of Moulvibazar district [103]; diabetes by TMPs of the Soren clan of the Santal tribe in Rajshahi district [104]; anthelmintic, dysentery, rectal diseases, cough, cold, mucus, fever by FMPs of Bheramara area in Kushtia district [110]; indigestion in Rampal sub-district of Bagerhat district [113].
*Anthocephalus chinensis* Hassk.	Snake bite by FMPs of Sylhet Division, Bangladesh [41]; scar, myopathic spasm, indigestion in humans, flatulency in cattle by FMPs in villages by the Bangali River of Bogra district [48]; infertility in men or women, infections in diabetic patients, bloating in cattle by FMPs in villages by the Padma River of Rajshahi district [48]; mouth wounds by FMPs of Vasu Bihar village, Bogra district [49]; elephantiasis, any problem of scrotum by FMPs of Balidha village, Jessore district [50]; helminthiasis in children, tumor, jaundice by FMPs of Shetabganj village, Dinajpur district [32]; piles by FMPs of six villages in Greater Naogaon district [53]; fever, snake bite by FMPs of three areas in Pirojpur district [74]; helminthiasis, tumor, swelling by FMPs of Dhamrai sub-district, Dhaka district [97]; headache, burns, eczema, itches by FMPs of Barisal Town, Barisal district [98]; fever, coughs, eye diseases, labor pain by FMPs of Boalia sub-district, Rajshahi district [109]; tumor by the Santal tribe residing in Thakurgaon district [111].
*Aquilaria malaccensis* Lamk.	Headache by FMPs of Dinajpur district [45].
*Asparagus racemosus* Willd.	Snake bite, wounds by the Garo tribe living in Netrakona district [19]; diabetes, tuberculosis in Shitol Para village, Jhalokati district [42]; sexual disorders, anti-hemorrhagic, sore throat, night blindness, blood dysentery by FMPs of three villages in Natore and Rajshahi districts [43]; physical and mental weakness by FMPs of Dinajpur district [45]; bacterial or fungal infections, edema, tonic, bloating, hypertension, to increase lactation, malnutrition in children, to increase memory, nerve weakness, to increase strength by FMPs of Noakhali district [47]; burning sensation during urination, bloating by FMPs in villages by the Ghaghot River of Rangpur district [48]; debility, to keep body healthy by FMPs of Station Purbo Para village, Jamalpur district [33]; physical weakness in males by FMPs of Shetabganj village, Dinajpur district [32]; constipation, impotency in men by FMPs of Vitbilia village in Pabna district [52]; impotency in males, to increase libido by FMPs of six villages in Greater Naogaon district [53]; hypertension, to increase lactation in nursing mother by FMPs of seven villages in Ishwardi Upazilla, Pabna district [54]; gonorrhea, spermatorrhea, erectile dysfunction, premature ejaculation, frequent urge for urination but only 1–2 drops of urine coming out each time by a FMP of Gachabari village in Tangail district [56]; weakness, diabetes, urinary problems by FMPs of four villages in Natore and Rajshahi districts [61]; sexual weakness, physical weakness by TMPs of 15 clans of the Garo tribe of Madhupur, Tangail district [63]; night blindness, blood dysentery, filariasis by a FMP of Jhalokathi in Barisal district [65]; used as preventive medicine against spermatorrhea and cardiovascular disorders as well as to raise body resistance against diseases and to keep the body healthy and mind contented by FMP of Jhenidah district [66]; body pain, leucorrhea by TMPs of two Marma tribal communities in two villages of Khagrachhari district [69]; to increase lactation by FMPs and TMPs in the vicinity of Lawachara Forest Reserve, Moulvibazar district [72]; physical weakness by TMPs of Kole and Rai tribes of Rajshahi and Nawabganj districts [73]; tuberculosis by the Santal tribe residing in Rajshahi district [77]; asthma during winter, all food tasting bitter, nutritive, to increase intelligence, to maintain good eyes, to increase sperm, to increase lactation, to increase strength, diarrhoea by FMPs of three villages in Sreepur Upazilla, Magura district [80]; burning sensations during urination, weakness by TMPs of Bongshi tribe in Tangail district [82]; asthma, leucorrhea by TMPs of Rai Kshatriya tribe of Pabna district [85]; swelling or enlargement of testicles by TMPs of the Pankho tribe of Bilaichari Union, Rangamati district [86]; asthma during winter, bitter taste in mouth, nutritive, to increase intelligence, to maintain good eyes, to increase sperm, to increase lactation, to increase strength, diarrhea, hyperacidity by FMPs of two villages in Bagerhat district [88]; snake bite by TMPs of the Khatriya and Kashya clans of the Bagdi tribe in Rajbari district [89]; physical weakness by a Kush tribal practitioner practicing among Garo and Kush tribes in Sherpur district; physical weakness by a Garo tribal practitioner practicing among Garo and Kush tribes in Sherpur district [93]; asthma, cough, cold by TMPs of Tonchongya tribe of Roangchaari Upazila in Bandarban district [99]; all diseases by a TMP of the Deb barma clan of the Tripura tribe of Moulvibazar district [103]; stone lodged in penis, diabetes by FMPs of Bheramara area in Kushtia district [110].
*Averrhoa carambola* L.	Diarrhea, vomiting, influenza by the Garo tribe living in Netrakona district [19]; coughs and mucus in two villages of Rajshahi district [38]; eczema, digestive aid, to keep body cool by FMPs of Sylhet Division, Bangladesh [41]; to stop bleeding, bone fractures in Shitol Para village, Jhalokati district [42]; tonic, appetizer by FMPs of three villages in Natore and Rajshahi districts [43]; dandruff by FMPs of Station Purbo Para village, Jamalpur district [33]; bone fracture, jaundice, bleeding from haemorrhoids by FMPs of seven villages in Ishwardi Upazilla, Pabna district [54]; stoppage of menstruation by FMPs of three villages in Kurigram district [58]; frequent coughs by a FMP of a village in Faridpur district [40]; constipation by TMPs of two Marma tribal communities in two villages of Khagrachhari district [69]; sperm incapable of being fertilized, constipation, coughs, flatulency by FMPs of two villages in Bagerhat district [88]; jaundice by a FMP practicing among tea garden workers in Sreemangal, Maulvibazar district [92]; liver diseases, fever, jaundice by a FMP of Sreemangal Upazila in Maulvibazar district [105].
*Azadirachta indica* A. Juss.	Fever, chicken pox, measles, skin disease by the Garo tribe living in Netrakona district [19]; itch, scabies, allergy, pus formation, skin disorders in Shitol Para village, Jhalokati district [42]; dental diseases, scabies by FMPs of Daudkandi sub-district of Comilla district [44]; syphilis, skin diseases, scabies, leprosy by FMPs in villages by the Ghaghot River of Rangpur district [48]; helminthiasis, antibacterial, itch by FMPs in villages by the Padma River of Rajshahi district [48]; insecticide by FMPs of Vasu Bihar village, Bogra district [49]; considered useful in any type of disease by FMPs of Balidha village, Jessore district [50]; skin diseases, body ache, bone pain, diabetes, measles, pox, itches, scabies, indigestion, cataract, decreased eye sight, abscess by FMPs of Station Purbo Para village, Jamalpur district [33]; helminthiasis, hepatic pain by FMPs of Shetabganj village, Dinajpur district [32]; regularize menstruation, diabetes by FMPs of Daulatdia Ghat, Kushtia district [51]; antiseptic, skin diseases, helminthiasis, pimples, acidity, blood purifier by FMPs of seven villages in Ishwardi Upazilla, Pabna district [54]; skin diseases, tooth infections, helminthiasis by FMPs of a village in Narayanganj district [55]; toothache, helminthiasis, skin diseases, chicken pox by a FMP of Gachabari village in Tangail district [56]; fever, pain, itches, rheumatic pain, skin infections, bleeding from gums, swelling of gums, tingling sensation in gums, to remove foul odor from mouth by FMPs of three villages in Kurigram district [58]; stomach and heart disorders by TMPs of Goala tribe of Moulvibazar district [59]; oral hygiene, itching, pain by FMPs of four villages in Natore and Rajshahi districts [61]; diarrhea, blood purifier, loss of appetite by TMPs of 15 clans of the Garo tribe of Madhupur, Tangail district [63]; leprosy, helmintic infections in children, acne, allergy, blood purification by a FMP of Jhalokathi in Barisal district [65]; burns, large size cuts and wounds by FMP from Jhenaidah district [66]; itch by the tribal healers of Oraon tribe of Sylhet district [67]; swelling of veins, scabies, helminthiasis, nocturnal emissions by the TMPs of the Rai Clan of the Tipra tribe of Sylhet Division [68]; helminthiasis, skin diseases by TMPs of two Marma tribal communities in two villages of Khagrachhari district [69]; skin infections by FMPs and TMPs in the vicinity of Lawachara Forest Reserve, Moulvibazar district [72]; scabies, eczema, itches by FMPs of Badarganj and Shekhertek villages in Rangpur district [79]; diarrhoea, blood purifier, loss of appetite, indigestion by FMPs of Terbaria and Babla villages in Tangail district [81]; good for eyes, increases lung capacity, fatigue, thirsts, coughs, fever, loss of appetite, helminthiasis, acne, biliary disorders, leprosy, wasting away of body, diabetes by FMPs of three villages in Sreepur Upazilla, Magura district [80]; skin diseases, allergy by TMPs of Bongshi tribe in Tangail district [82]; severe pain, whitish discharge from vagina, hair loss, rheumatic pain by FMPs of several areas of Faridpur and Rajbari districts [83]; paralysis, skin infections by TMPs of Rai Kshatriya tribe of Pabna district [85]; diabetes by TMPs of the Pankho tribe of Bilaichari Union, Rangamati district [86]; fever, pain, to prevent tooth infections by TMPs of the Murmu tribal community residing in Rajshahi district [87]; pimple, tiredness, coughs, vomiting, helminthiasis, gall bladder problems, pain, dyspepsia, leprosy, acne, gleet, gonorrhea, diabetes by FMPs of two villages in Bagerhat district [88]; skin disorders, tooth infections, foul odor in mouth by TMPs of the Tripura tribe residing in Comilla district [90]; diabetes by a Chakma tribal practitioner practicing among Garo and Kush tribes in Sherpur district; skin diseases by a Kush TMP practicing among Garo and Kush tribes in Sherpur district [93]; skin diseases, toothache by folk herbalists in Comilla district [94]; skin diseases, helminthiasis by TMPs of the Harbang clan of the Tripura tribe of Mirsharai area, Chittagong district [95]; itches, ringworm, helminthiasis, chicken pox by a TMP of the Deb barma clan of the Tripura tribe of Moulvibazar district [124]; diabetes by FMPs of Barisal Town, Barisal district [98]; skin diseases, itches, helminthiasis (especially in children), cough-induced chest pain by TMPs of the Manipuri tribe in Kamalganj Upazila, Moulvibazar district [101]; helminthiasis, fever with shivering by TMPs of the Bauri tribal community of Moulvibazar district [102]; blood purifier by TMPs of the Soren clan of the Santal tribe in Rajshahi district [104]; body pain, itches by a FMP of Sreemangal Upazila in Maulvibazar district [105]; to induce fertility, cancer, acne, itch, carminative by TMPs of Santal tribe of Rangpur district [107]; cancer, skin diseases, helminthiasis, wounds, diabetes, rheumatoid arthritis by FMPs of Boalia sub-district, Rajshahi district [109]; fever, fever arising from gall bladder disorders by FMPs of Bheramara area in Kushtia district [110]; abscess by the Santal tribe residing in Thakurgaon district [111]; to strengthen base of tooth, acne by a FMP of Savar in Dhaka district [114]; scabies, itches by TMPs of the Sigibe clan of the Khumi tribe of Thanchi sub-district in Bandarban district [115]; infections of the tooth by a TMP of the Sardar (Dhangor) community in Chuadanga district [116]; cuts and wounds, allergy, premature graying of hair by TMPs of Khasia tribe in several sub-districts in Sylhet district [117].
*Bombax ceiba* L.	Urinary calculus, loss of libido by the Garo tribe living in Netrakona district [19]; low semen density in two villages of Rajshahi district [38]; loss of libido in Vasu Bihar village, Bogra district [39]; stoppage of urination, debility, sexual weakness, to increase semen by FMPs of Sylhet Division, Bangladesh [41]; acne, spermatorrhea, leucorrhea, presence of blood in vomit or bleeding through nose due to liver disorders, blood clotting problems or tuberculosis in Shitol Para village, Jhalokati district [42]; tonic, sexual disorders, edema by FMPs of three villages in Natore and Rajshahi districts [43]; to increase sexual activity by FMPs of Daudkandi sub-district of Comilla district [44]; debility, to increase growth by FMPs of Dinajpur district [45]; gonorrhea by the Garo tribe inhabiting the Madhupur forest region of Bangladesh [46]; sex stimulant by FMPs in villages by the Ghaghot River of Rangpur district [48]; erectile dysfunction, snake bite by FMPs in villages by the Padma River of Rajshahi district [48]; loss of libido by FMPs of Vasu Bihar village, Bogra district [49]; aphrodisiac, passing of semen with urine by FMPs of Balidha village, Jessore district [50]; to keep body healthy, to increase sexual power by FMPs of Station Purbo Para village, Jamalpur district [33]; sex stimulant by FMPs of Shetabganj village, Dinajpur district [32]; impotency by FMPs of Daulatdia Ghat, Kushtia district [51]; physical weakness, stoppage of urination by FMPs of six villages in Greater Naogaon district [53]; pimple, spermatorrhea by FMPs of seven villages in Ishwardi Upazilla, Pabna district [54]; to increase libido, sexually transmitted diseases by FMPs of three villages in Kurigram district [58]; sexual weakness by a FMP of Savar in Dhaka district [60]; osteoporosis, blood dysentery, piles by FMPs of four villages in Natore and Rajshahi districts [61]; leucorrhea by TMPs of 15 clans of the Garo tribe of Madhupur, Tangail district [63]; used as preventive medicine against spermatorrhea and cardiovascular disorders as well as to raise body resistance against diseases and to keep the body healthy and mind contented by FMP of Jhenidah district [66]; decreased semen density, erectile dysfunction by the tribal healers of Oraon tribe of Sylhet district [67]; physical weakness, sexual weakness, puerperal fever by the TMPs of the Rai Clan of the Tipra tribe of Sylhet Division [68]; diabetes by a Pahan TMP in Dinajpur district [71]; weakness, diarrhea by FMPs of three areas in Pirojpur district [74]; to increase libido, gastric ulcer by FMPs of Badarganj and Shekhertek villages in Rangpur district [79]; passing of semen with urine by FMPs of several areas of Faridpur and Rajbari districts [83]; diarrhea, dysentery by TMPs of Rai Kshatriya tribe of Pabna district [85]; diarthea, indigestion, burning sensations due to dehydration by FMPs of two villages in Bagerhat district [88]; physical weakness by a Chakma TMP practicing among Garo and Kush tribes in Sherpur district [93]; debility, infrequent urination by FMPs of Dhamrai sub-district, Dhaka district [97]; stoppage of urination and defecation by FMPs of Barisal Town, Barisal district [98]; being touched by ‘evil wind’ by TMPs of the Soren clan of the Santal tribe in Rajshahi district [104]; to increase sperm count by Christians living in Mirzapur village of Dinajpur ditrict, Bangladesh [108]; to increase sperm count by FMPs of Bheramara area in Kushtia district [110]; gonorrhea, acne by a FMP of Jamalpur district [106]; having difficulties in urinating by TMPs of Mro community of Gazalia Union in Bandarbans district [118].
*Brassica oleracea* L.	
*Calendula officinalis* L.	Ear ache, skin infections, insect bite by FMPs of Bheramara area in Kushtia district [110].
*Carica papaya* L.	Dysentery, ringworm by the Garo tribe living in Netrakona district [19]; blood with cough, eczema, piles by FMPs of Sylhet Division, Bangladesh [41]; fever, blood dysentery by FMPs of Daudkandi sub-district of Comilla district [44]; digestive aid, to increase strength, respiratory problems, diabetes by FMPs in villages by the Padma River of Rajshahi district [48]; indigestion, bloating, stomach ache, jaundice, toothache, headache, kidney stones by FMPs of Station Purbo Para village, Jamalpur district [33]; piles by FMPs of six villages in Greater Naogaon district [53]; burning sensations and pain in the stomach, loss of appetite, indigestion by FMPs of a village in Narayanganj district [55]; to maintain healthy liver by FMPs of two villages in Natore district [31]; jaundice, diabetes by the tribal healers of Oraon tribe of Sylhet district [67]; spleen enlargement, eczema, loss of appetite, indigestion, constipation by the TMPs of the Rai Clan of the Tipra tribe of Sylhet Division [68]; dengue fever by FMPs and TMPs in the vicinity of Lawachara Forest Reserve, Moulvibazar district [72]; jaundice, to keep stomach cool by FMPs of three areas in Pirojpur district [74]; hematemesis, piles, liver, spleen and hepatic impairment, constipation by FMPs of two villages in Bagerhat district [88]; jaundice by the Teli clan of the Telegu tribe of Maulvibazar district [100]; constipation, anti-inflammatory, diabetes, anorexia, abortifacient by TMPs of Santal tribe of Rangpur district [107]; tuberculosis, constipation, helminthiasis, cooling, leucoderma, ecbolic, fever by FMPs of Boalia sub-district, Rajshahi district [109].
*Cassia alata* L*.*	Ring worm by the Garo tribe living in Netrakona district [19]; ringworm in Shitol Para village, Jhalokati district [42]; eczema, wound, anthelmintic, dermatitis, leucorrhea by FMPs of three villages in Natore and Rajshahi districts [43]; scabies, skin diseases by FMPs of Dinajpur district [45]; skin diseases by the Garo tribe inhabiting the Madhupur forest region of Bangladesh [46]; skin diseases by FMPs of Noakhali district [47]; ringworm by FMPs of Balidha village, Jessore district [50]; scabies by FMPs of Station Purbo Para village, Jamalpur district [33]; skin diseases by tribal medicinal practitioners (TMPs) of the Chakma tribe residing in Rangamati district [57]; eczema by TMPs of Goala tribe of Moulvibazar district [59]; sexual disorder by FMPs of four villages in Natore and Rajshahi districts [61]; eczema, scabies, skin infections by TMPs of 15 clans of the Garo tribe of Madhupur, Tangail district [63]; skin diseases by FMPs and TMPs in the vicinity of Lawachara Forest Reserve, Moulvibazar district [72]; ringworm, eczema by the Marma tribe living in Naikhongchhari, Bandarban district [75]; ringworm by the Tripura tribe residing in Chittagong Hill Tracts, Bangladesh [78]; skin disorder, eczema by TMPs of the Pankho tribe of Bilaichari Union, Rangamati district [86]; eczema by TMPs of the Khatriya and Kashya clans of the Bagdi tribe in Rajbari district [89]; skin infections by a Tonchongya tribal healer of Rangamati district [91]; eczema, any type of skin disorder by TMPs of the Harbang clan of the Tripura tribe of Mirsharai area, Chittagong district [95]; ringworm, eczema, itch, scabies, skin disease by TMPs of Tonchongya tribe of Roangchaari Upazila in Bandarban district [99]; skin diseases by TMPs of the Manipuri tribe in Kamalganj Upazila, Moulvibazar district [101]; eczema by a TMP of the Deb barma clan of the Tripura tribe of Moulvibazar district [103]; eczema by a FMP of Sreemangal Upazila in Maulvibazar district [105]; nocturnal emissions by FMPs of Bheramara area in Kushtia district [110]; stomach pain due to bloating or indigestion by TMPs of Tonchongya tribe of Bandarban district [114]; itches, skin disorders by a FMP of Aria Bazar village, Bogra district [119].
*Cassia fistula* L.	Cancer, injury, dermatitis by the Garo tribe living in Netrakona district [19]; asthma by FMPs of two villages in Rajshahi district [38]; helminthiasis in Shitol Para village, Jhalokati district [42]; long-term cough, nervous weakness, constipation by FMPs of Dinajpur district [45]; cracking of skin and helminthiasis in humans, dysentery of cattle by the Garo tribe inhabiting the Madhupur forest region of Bangladesh [46]; helminthiasis by FMPs of Noakhali district [47]; anal disorders (prolapse) by FMPs of Daulatdia Ghat, Kushtia district [51]; eczema, waist pain, coughs by a FMP of Savar in Dhaka district [60]; diarrhea by FMP from Jhenaidah district [66]; tonsillitis, rheumatism, leprosy by the TMPs of the Rai Clan of the Tipra tribe of Sylhet Division [68]; stomach pain by TMPs of two Marma tribal communities in two villages of Khagrachhari district [69]; tuberculosis by a Pahan TMP in Dinajpur district [71]; fevers, to stimulate appetite by the Marma tribe living in Naikhongchhari, Bandarban district [75]; coughs, obesity, laxative, fever, heart disorders, biliary problem, carminative, piles, constipation, leprosy, coughs by FMPs of three villages in Sreepur Upazilla, Magura district [80]; jaundice by TMPs of Rai Kshatriya tribe of Pabna district [85]; constipation in children by TMPs of the Pankho tribe of Bilaichari Union, Rangamati district [86]; constipation in children by a Tonchongya tribal healer of Rangamati district [91]; snake repellent, eczema by folk herbalists in Comilla district [94]; bloating, urinary problems, stoppage of urination by TMPs of the Harbang clan of the Tripura tribe of Mirsharai area, Chittagong district [95]; purgative by FMPs of Barisal Town, Barisal district [98]; coughs, helminthiasis, diabetes, irregular urination, edema, constipation by TMPs of Tonchongya tribe of Roangchaari Upazila in Bandarban district [99]; stomach pain by a FMP of Sreemangal Upazila in Maulvibazar district [105]; leprosy, tonsillitis by Christians living in Mirzapur village of Dinajpur ditrict, Bangladesh [108]; gastrointestinal disorders in goats by the Santal tribe residing in Thakurgaon district [111];.
*Cassia occidentalis* L.	Paralysis in Faridpur district [40]; itch, appetizer, antiinflammatory, asthma, whooping cough, leucoderma by FMPs of three villages in Natore and Rajshahi districts [43]; eczema, gastric problems by FMPs of Dinajpur district [45]; nocturnal dyspnoea by FMPs in villages by the Ghaghot River of Rangpur district [48]; stomach ache by FMPs in villages by the Padma River of Rajshahi district [48]; gastrointestinal problems, stomach ache by FMPs of Shetabganj village, Dinajpur district [32]; boils, skin diseases, coughs, mucus, blood purifier by FMPs of Daulatdia Ghat, Kushtia district [51]; skin infections, scabies by FMPs of Chuadanga district [66]; paralysis by a FMP of a village in Faridpur district [40]; tonic, diabetes, malaria, fistula, to induce fertility in men by TMPs of Santal tribe of Rangpur district [107]; rheumatism by Christians living in Mirzapur village of Dinajpur ditrict, Bangladesh [108]; body poisoning, gall bladder problems, constipation by FMPs of Bheramara area in Kushtia district [110].
*Centella asiatica* (L.) Urb.	Dysentery and intestinal pain by the Garo tribe living in Netrakona district [19]; weakness in two villages of Rajshahi district [38]; dysentery in Vasu Bihar village, Bogra district [39]; dysentery and blood dysentery in Faridpur district [40]; indigestion, appetite stimulant by FMPs of Sylhet Division, Bangladesh [41]; dysentery, stomach ache, to increase memory in Shitol Para village, Jhalokati district [42]; body ache, dysentery by FMPs of Daudkandi sub-district of Comilla district [44]; indigestion, stomach infection by the Garo tribe inhabiting the Madhupur forest region of Bangladesh [46]; weakness, skin problems, dysentery, indigestion, cataract, gonorrhea, low semen, leucorrhea by FMPs of Noakhali district [47]; bone fracture by FMPs in villages by the Ghaghot River of Rangpur district [48]; sexual diseases by FMPs in villages by the Padma River of Rajshahi district [48]; dysentery by FMPs of Vasu Bihar village, Bogra district [49]; to keep head cool, diabetes, swelling in eyes, conjunctivitis in humans, cataract in goats by FMPs of Station Purbo Para village, Jamalpur district [33]; hair loss, dysentery, gastrointestinal disorders, injury by FMPs of Shetabganj village, Dinajpur district [32]; blood purifier, fever, diabetes by FMPs of Daulatdia Ghat, Kushtia district [51]; anemia, vomiting, stomach pain by FMPs of six villages in Greater Naogaon district [53]; cold, dysentery, blood purifier by FMPs of seven villages in Ishwardi Upazilla, Pabna district [54]; diarrhea, dysentery by FMPs of a village in Narayanganj district [55]; stomach pain, swelling (edema) in hands or legs by a FMP of Gachabari village in Tangail district [56]; flatulence, indigestion, hair loss by FMPs of three villages in Kurigram district [58]; to increase memory by a FMP of Savar in Dhaka district [60]; stomach pain, dysentery by TMPs of the Tudu sub-clan of the Santal tribe in Joypurhat district [62]; bloating, diarrhea, dysentery, to increase memory by FMPs of Bhola district [64]; dysentery by FMPs of two villages in Natore district [31]; dysentery, intestinal dysfunction by the tribal healers of Gor tribe of Sylhet district; to increase energy by the tribal healers of Oraon tribe of Sylhet district [67]; headache, stuttering in children by the TMPs of the Rai Clan of the Tipra tribe of Sylhet Division [68]; dysentery, blood dysentery by a FMP of a village in Faridpur district [40]; dysentery, abdominal pain by TMPs of two Marma tribal communities in two villages of Khagrachhari district [69]; dysentery, to enhance memory by a Pahan TMP in Dinajpur district [71]; diarrhea, dysentery, stomach pain by FMPs and TMPs in the vicinity of Lawachara Forest Reserve, Moulvibazar district [72]; ulcer by FMPs of three areas in Pirojpur district [74]; lack of breast milk following childbirth by the Santal tribe residing in Rajshahi district [77]; gastric disorder, stomach pain, diarrhea, blood dysentery, fever, cough by the Tripura tribe residing in Chittagong Hill Tracts, Bangladesh [78]; gastrointestinal disorders by FMPs of Badarganj and Shekhertek villages in Rangpur district [79]; fever, pain by FMPs of Terbaria and Babla villages in Tangail district [81]; jaundice, dysentery by FMPs of several areas of Faridpur and Rajbari districts [83]; stomach pain by TMPs of Naik clan of Rajbongshi tribe of Moulvibazar district [84]; stomach pain in children, dysentery by TMPs of Rai Kshatriya tribe of Pabna district [85]; rheumatic problems, gastric problems by a Kush tribal practitioner practicing among Garo and Kush tribes in Sherpur district [93]; bone fracture by folk herbalists in Comilla district [94]; helminthiasis, stomach ache by TMPs of the Harbang clan of the Tripura tribe of Mirsharai area, Chittagong district [95]; loss of appetite, diarrhea by TMPs of the Nag clan of the Rai Ghatual tribe in Moulvibazar district [96]; diarrhoea, gastric problems by FMPs of Dhamrai sub-district, Dhaka district [97]; diarrhea, dysentery by the Teli clan of the Telegu tribe of Maulvibazar district [100]; abdominal pain, gastric trouble by TMPs of the Manipuri tribe in Kamalganj Upazila, Moulvibazar district [101]; dysentery by TMPs of the Bauri tribal community of Moulvibazar district [102]; stomach disorders by a TMP of the Deb barma clan of the Tripura tribe of Moulvibazar district [103]; jaundice by TMPs of the Hodi tribe in Sherpur district [112]; anemia by TMPs of Mro community of Gazalia Union in Bandarbans district [118].
*Cinnamomum tamala* (Buch.-Ham.) T.Nees & C.H.Eberm	Bloating, indigestion by FMPs of Sylhet Division, Bangladesh [41]; excessive sweat, appearance of small pustules on the body due to excessive heat or sweating which itches in Shitol Para village, Jhalokati district [42]; for strong teeth by FMPs of Daudkandi sub-district of Comilla district [44]; coughs, cold by FMPs of Dinajpur district [45]; whooping cough by FMPs of six villages in Greater Naogaon district [53]; diabetes, cold by FMPs of four villages in Natore and Rajshahi districts [61]; coughs, vomiting, loss of appetite by FMPs of Bhola district [64]; influenza by a FMP of Jhalokathi in Barisal district [65]; infertility in woman by FMPs of Chuadanga district [66]; puerperal fever, rheumatic pain by the TMPs of the Rai Clan of the Tipra tribe of Sylhet Division [68]; excessive sexual desire, coughs, bloating, piles, loss of appetite, sexual disorder by FMPs of three villages in Sreepur Upazilla, Magura district [80]; excessive sexual desire, coughs, flatulence, piles, bloating, loss of appetite, sexual disorder by FMPs of two villages in Bagerhat district [88]; coughs, bloating, appetite stimulant, biliary disorders, piles by the Santal tribe residing in Thakurgaon district [111]; colic in Paikgacha sub-district of Khulna district [113].
*Cinnamomum verum* J. Presl.	diabetes by FMPs of Station Purbo Para village, Jamalpur district [33]; bone fracture, asthma, uterine problems by FMPs of several areas of Faridpur and Rajbari districts [55]; infertility in woman by FMPs of Chuadanga district [66]; stomach pain, puerperal fever, rheumatic pain, stomach pain by the TMPs of the Rai Clan of the Tipra tribe of Sylhet Division [68]; any type of cancer by TMPs of Kole and Rai tribes of Rajshahi and Nawabganj districts [73]; pain by TMPs of the Nag clan of the Rai Ghatual tribe in Moulvibazar district [96]; to strengthen stomach by a FMP of Jamalpur district [106]; to increase sperm count, biliary disorders, rheumatism by the Santal tribe residing in Thakurgaon district [111].
*Cissus quadrangularis* L.	Wounds and sprains by the Garo tribe living in Netrakona district [19]; bone fracture in two villages of Rajshahi district [38]; tonic, sprain, sedative by FMPs of three villages in Natore and Rajshahi districts [43]; bone fracture by FMPs of Daudkandi sub-district of Comilla district [44]; bone fracture by the Garo tribe inhabiting the Madhupur forest region of Bangladesh [46]; rheumatic fever, joint pain by FMPs of Noakhali district, pain by FMPs of Feni district [47]; bone fracture by FMPs of Balidha village, Jessore district [50]; bone fracture by FMPs of Station Purbo Para village, Jamalpur district [33]; bone fracture by FMPs of Shetabganj village, Dinajpur district [32]; bone fracture by FMPs of Vitbilia village in Pabna district [52]; bone fracture, sprain by FMPs of three villages in Kurigram district [58]; bone fracture in hand or leg by TMPs of 15 clans of the Garo tribe of Madhupur, Tangail district [63]; bone fracture by FMPs of two villages in Natore district [31]; bone fracture in hands or legs by FMP from Jhenaidah district [66]; bone fracture by a Pahan TMP in Dinajpur district [71]; bone fracture in hands or legs by TMPs of Kole and Rai tribes of Rajshahi and Nawabganj districts [73].bone fracture by the Santal tribe residing in Rajshahi district [77]; bone fracture by TMPs of Bongshi tribe in Tangail district [82]; bone fracture by FMPs of several areas of Faridpur and Rajbari districts [83]; bone fracture by TMPs of the Murmu tribal community residing in Rajshahi district [87]; bone fracture by TMPs of the Khatriya and Kashya clans of the Bagdi tribe in Rajbari district [89]; bone fracture by FMPs of Dinajpur district [97]; bone fracture by TMPs of the Manipuri tribe in Kamalganj Upazila, Moulvibazar district [101]; bone fracture by a FMP of Sreemangal Upazila in Maulvibazar district [105]; sprain in hand or leg constipation by a Kush TMP practicing among Garo and Kush tribes in Sherpur district [109]; bone fracture by FMPs of Bheramara area in Kushtia district [110]; bone fractures, pain due to fractures by the Santal tribe residing in Thakurgaon district [111]; indigestion in Paikgacha sub-district of Khulna district [113];
*Citrus acida* Pers*.*	Dandruff, vomiting by FMPs of Daudkandi sub-district of Comilla district [44]; loss of appetite, indigestion, vomiting tendency, acne, dandruff by FMPs of Station Purbo Para village, Jamalpur district [33]; facial scars and spots, vitamin C deficiency by FMPs of Terbaria and Babla villages in Tangail district [81]; helminthiasis, abdominal discomfort, to increase appetite, flatulence, coughs, piles by FMPs of two villages in Bagerhat district [88]; carminative, gall bladder diseases by FMPs of Bheramara area in Kushtia district [110]; to keep head cool, restless feeling by the Santal tribe residing in Thakurgaon district [111].
*Citrus grandis* Hassk.	Appetite stimulant, vomiting, fever by FMPs of Sylhet Division, Bangladesh [41]; aphrodisiac by FMPs of Balidha village, Jessore district [50]; to increase appetite, blood purifier, fever by FMPs of Station Purbo Para village, Jamalpur district [33]; loss of appetite, vomiting, fever by the tribal healers of Oraon tribe of Sylhet district [67]; fever by FMPs of three areas in Pirojpur district [74]; to increase strength, carminative, indigestion by FMPs of Bheramara area in Kushtia district [110]; deformities in head of young children by the Santal tribe residing in Thakurgaon district [111].
*Clerodendrum viscosum* Vent.	Lice infections by the Garo tribe living in Netrakona district [19]; gastrointestinal disorders in Vasu Bihar village, Bogra district [39] coughs in children by FMPs of Sylhet Division, Bangladesh [41]; nausea, vomiting, puerperal fever in Shitol Para village, Jhalokati district [42]; tonic, gastritis, dermatitis, dysentery by FMPs of three villages in Natore and Rajshahi districts [43]; helminthiasis, dysentery, jaundice by FMPs of Dinajpur district [45] colic pain by the Garo tribe inhabiting the Madhupur forest region of Bangladesh [46]; coughs, asthma, skin diseases, snake bite, gonorrhea, low semen, leucorrhea by FMPs of Noakhali district [47]; itch by FMPs in villages by the Padma River of Rajshahi district [48]; gastrointestinal disorders by FMPs of Vasu Bihar village, Bogra district [49]; helminthiasis by FMPs of Balidha village, Jessore district [50]; blood dysentery, dysentery, infections by FMPs of Station Purbo Para village, Jamalpur district [33]; pain by FMPs of Shetabganj village, Dinajpur district [32]; fever, burning sensations in the body, helminthiasis by FMPs of six villages in Greater Naogaon district [53]; skin diseases, sexual weakness by FMPs of seven villages in Ishwardi Upazilla, Pabna district [54]; frequent urination, diabetes by tribal medicinal practitioners (TMPs) of the Chakma tribe residing in Rangamati district [57]; liver problems by a FMP of Savar in Dhaka district [60]; helminthiasis, rheumatic pain by FMPs of Bhola district [64]; coughs by the tribal healers of Gor tribe of Sylhet district; anthrax in cattle by the tribal healers of Oraon tribe of Sylhet district [67]; stomach pain by the Marma tribe living in Naikhongchhari, Bandarban district [75];]; helminthiasis, toothache, lesions within the ear, fever with convulsions, malaria by the Rakhain tribe inhabiting the Chittagong Hill Tracts region [76]; stomach pain, acidity, redness of eye, malaria fever, fever, cough, helminthiasis, respiratory problem, aphrodisiac, analgesic by the Tripura tribe residing in Chittagong Hill Tracts, Bangladesh [78]; helminthiasis, frequent urination by FMPs of Badarganj and Shekhertek villages in Rangpur district [79]; pain in body, blood purifier by FMPs of Terbaria and Babla villages in Tangail district [81]; pain by TMPs of Bongshi tribe in Tangail district [82]; malaria fever, any type of stomach pain by TMPs of Tonchongya tribe of Bandarban district [82]; feeling of weakness during time of menstruation by TMPs of the Harbang clan of the Tripura tribe of Mirsharai area, Chittagong district [95]; fever in children, toothache, pain in gums by FMPs of Dhamrai sub-district, Dhaka district [97]; hookworm infection by FMPs of Barisal Town, Barisal district [98]; stomach pain by TMPs of Chakma tribe of Rangapanir Chara Area in Khagrachaari district [34]; jaundice, helminthiasis by a TMP of the Deb barma clan of the Tripura tribe of Moulvibazar district [103]; jaundice by a FMP of Sreemangal Upazila in Maulvibazar district [105]; skin eruption, fever, dysentery by TMPs of Santal tribe of Rangpur district [107]; helminthiasis, ulcer by Christians living in Mirzapur village of Dinajpur ditrict, Bangladesh [108]; helminthiasis, infections from scorpion bites by the Santal tribe residing in Thakurgaon district [111]; burning sensations in the chest, salty taste in mouth when burping, flatulency, gastric pain by TMPs of the Sigibe clan of the Khumi tribe of Thanchi sub-district in Bandarban district [115].
*Coccinia cordifolia* (L.) Cogn.	Total paralysis or numbness of body, burning sensations in head or soles of feet by FMPs of two villages in Rajshahi district [38]; burning sensations during urination, diabetes by FMPs of Sylhet Division, Bangladesh [41]; burning sensations in the body, blood dysentery, scabies, leucoderma, diabetes in Shitol Para village, Jhalokati district [42]; coughs, diabetes, dysentery, emetic, burn by FMPs of three villages in Natore and Rajshahi districts [43]; mental disease, diabetes by FMPs of Daudkandi sub-district of Comilla district [44]; hypertension, diabetes by FMPs of Dinajpur district [45]; diabetes by FMPs of Feni district [47]; sunstroke, diabetes by FMPs in villages by the Bangali River of Bogra district [48]; headache by FMPs in villages by the Padma River of Rajshahi district [48]; diabetes, to keep head cool, dysentery, skin diseases, burning sensations in hands or feet by FMPs of Station Purbo Para village, Jamalpur district [33]; hematemesis, loss of appetite, diabetes, flatulency by FMPs of Shetabganj village, Dinajpur district [32]; typhoid, eczema, leucoderma, lesion on tongue by FMPs of Daulatdia Ghat, Kushtia district [51]; diabetes, jaundice by FMPs of Vitbilia village in Pabna district [52]; diabetes, debility, to keep head cool, burning sensations in the body by FMPs of six villages in Greater Naogaon district [53]; dysentery, burns by FMPs of seven villages in Ishwardi Upazilla, Pabna district [54]; diabetes, stomach pain by FMPs of a village in Narayanganj district [55]; diarrhea, dysentery by a FMP of Gachabari village in Tangail district [56]; menstrual problems like burning sensations during urination, frequent urination, diabetes by tribal medicinal practitioners (TMPs) of the Chakma tribe residing in Rangamati district [57]; diabetes, loss of appetite, flatulence by FMPs of three villages in Kurigram district [58]; burning sensations in the body, diabetes by a FMP of Savar in Dhaka district [60]; moisturizer for dry skin by FMPs of four villages in Natore and Rajshahi districts [61]; blood purifier, loss of appetite, diabetes, injury, sprains by TMPs of 15 clans of the Garo tribe of Madhupur, Tangail district [63]; coughs, bloating by FMPs of Bhola district [64]; diabetes, intestinal dysfunction by the tribal healers of Oraon tribe of Sylhet district [67]; diabetes, fever by the TMPs of the Rai Clan of the Tipra tribe of Sylhet Division [68]; diabetes by TMPs of two Marma tribal communities in two villages of Khagrachhari district [69]; abscess by TMPs of Kole and Rai tribes of Rajshahi and Nawabganj districts [73];diabetes, dysentery, flatulence, to keep stomach in good condition by FMPs of three areas in Pirojpur district [74]; diabetes, swellings, diarrhoea, blood purifier, loss of appetite, indigestion by FMPs of Terbaria and Babla villages in Tangail district [81]; to keep head cool, diabetes, dysentery, jaundice by FMPs of several areas of Faridpur and Rajbari districts [83]; diabetes by TMPs of Rai Kshatriya tribe of Pabna district [85]; diabetes, pain by TMPs of the Murmu tribal community residing in Rajshahi district [87]; skin disease, tumors, headache, pseudo-tumors, diabetes, jaundice, cataract, skin eruptions, laxative, gonorrhea by FMPs of two villages in Bagerhat district [88]; diabetes by TMPs of the Tripura tribe residing in Comilla district [90]; diabetes, dizziness by TMPs of the Harbang clan of the Tripura tribe of Mirsharai area, Chittagong district [95]; dysentery, oral lesions by FMPs of Dhamrai sub-district, Dhaka district [97]; to keep head cool, burning sensations in hands or feet, diabetes by FMPs of Barisal Town, Barisal district [98]; diabetes, fever, jaundice by TMPs of the Manipuri tribe in Kamalganj Upazila, Moulvibazar district [101]; diabetes by a FMP of Sreemangal Upazila in Maulvibazar district [105]; headache, lesions on tongue by a FMP of Jamalpur district [106];diabetes by Christians living in Mirzapur village of Dinajpur ditrict, Bangladesh [108]; diabetes, edema, eye diseases by FMPs of Boalia sub-district, Rajshahi district [109]; whitish discharge in urine of men by FMPs of Bheramara area in Kushtia district [110]; mental depression, disability to work, blood dysentery, body pain by the Santal tribe residing in Thakurgaon district [111]; rheumatic pain, sciatica by a TMP of the Sardar (Dhangor) community in Chuadanga district [116]; jaundice, diabetes by TMPs of Khasia tribe in several sub-districts in Sylhet district [117]; baldness, diabetes, sunstroke, scar by FMPs of two villages by the Rupsha River in Bagerhat district [120].
*Cocos nucifera* L.	Skin diseases, skin spots, diarrhea by the Garo tribe living in Netrakona district [19]; to strengthen hair, debility by FMPs of Sylhet Division, Bangladesh [41]; ringworm, oral infection, gingivitis, pain in animal in Shitol Para village, Jhalokati district [42]; diuretic, helminthiasis, jaundice, acne, lack of appetite by FMPs in villages by the Bangali River of Bogra district [48]; to keep head cool, diabetes by FMPs of Station Purbo Para village, Jamalpur district [33]; hair loss, skin sores, head lice by a FMP of Savar in Dhaka district [60]; skin disease by FMPs of three areas in Pirojpur district [74]; biliary problem, burning sensations from dehydration, hematemesis, hyperacidity by FMPs of two villages in Bagerhat district [88]; jaundice, anti-inflammatory, acne by TMPs of Santal tribe of Rangpur district [107]; syphilis, jaundice, diabetes, cholera by FMPs of Boalia sub-district, Rajshahi district [109].
*Colocasia esculenta* (L.) Schott	Cuts and wounds by the Garo tribe living in Netrakona district [19]; severe jaundice, digestive aid, constipation in Shitol Para village, Jhalokati district [42]; colic, indigestion by FMPs of three villages in Natore and Rajshahi districts [43]; astringent, carminative, scar, tumor, infertility in male or female by FMPs in villages by the Bangali River of Bogra district [48]; rheumatic pain, paralysis by FMPs of Shetabganj village, Dinajpur district [32]; rheumatic pain, paralysis by FMPs of three villages in Kurigram district [58]; severe headache by FMPs and TMPs in the vicinity of Lawachara Forest Reserve, Moulvibazar district [72]; cuts and wounds to stop bleeding, blood purifier, to strengthen bones by FMPs of Dhamrai sub-district, Dhaka district [97]; diabetes by the Teli clan of the Telegu tribe of Maulvibazar district [100]; anemia, malnutrition by TMPs of the Manipuri tribe in Kamalganj Upazila, Moulvibazar district [101]; rheumatic pain by a TMP of the Deb barma clan of the Tripura tribe of Moulvibazar district [103]; infections, whitish or darkish pathes of skin on face, infertility by a FMP of Jamalpur district [106]; piles, diarrhea, dysentery, wound by TMPs of Santal tribe of Rangpur district [107]; prolapse of uterus by TMPs of the Hodi tribe in Sherpur district [112]; astringent, dermatitis, bloating, tiger bite, helminthiasis, emetic by FMPs of two villages by the Rupsha River in Bagerhat district [120].
*Curcuma longa* L.	Skin diseases in Vasu Bihar village, Bogra district [39]; helminthiasis, itches by FMPs of Sylhet Division, Bangladesh [41]; jaundice, skin disorders, to increase brightness of skin in Shitol Para village, Jhalokati district [42]; gonorrhea, anthelmintic, sore throat, hepatitis, appetizer, allergy, eye disorders by FMPs of three villages in Natore and Rajshahi districts [43]; acne by FMPs of Daudkandi sub-district of Comilla district [44]; allergy, skin diseases, scabies, leprosy by FMPs in villages by the Ghaghot River of Rangpur district [48]; skin diseases by FMPs of Vasu Bihar village, Bogra district [49]; excessive bile secretion by FMPs of Balidha village, Jessore district [50]; allergy by FMPs of Station Purbo Para village, Jamalpur district [33]; filariasis by FMPs of Shetabganj village, Dinajpur district [32]; to improve skin texture, sprain by FMPs of Daulatdia Ghat, Kushtia district [51]; helminthiasis, skin diseases, loss of appetite, to increase memory by FMPs of a village in Narayanganj district [55]; hypertension, abscess by tribal medicinal practitioners (TMPs) of the Chakma tribe residing in Rangamati district [57]; bone fracture, sprain by FMPs of three villages in Kurigram district [58]; allergy by a FMP of Jhalokathi in Barisal district [65]; infertility in women, vomiting in children by FMPs of Chuadanga district [66]; bone fracture, sex stimulant by the tribal healers of Oraon tribe of Sylhet district [67]; puerperal fever, scabies by the TMPs of the Rai Clan of the Tipra tribe of Sylhet Division [68]; liver disorders by FMPs and TMPs in the vicinity of Lawachara Forest Reserve, Moulvibazar district [72]; kala azar by TMPs of Kole and Rai tribes of Rajshahi and Nawabganj districts [73]; arthritis, gout by FMPs of three areas in Pirojpur district [74]; nocturnal emission, scabies, eczema by FMPs of Terbaria and Babla villages in Tangail district [81]; excessive sexual desire, rheumatism, leprosy, diabetes, edema by FMPs of three villages in Sreepur Upazilla, Magura district [80]; to improve skin color, to control excessive sexual desire, rheumatism, leprosy, diabetes, edema by FMPs of two villages in Bagerhat district [88]; to whiten complexion by a FMP practicing among tea garden workers in Sreemangal, Maulvibazar district [92]; helminthiasis by folk herbalists in Comilla district [94]; diarrhea, dysentery by the Teli clan of the Telegu tribe of Maulvibazar district [100]; chicken pox by a TMP of the Deb barma clan of the Tripura tribe of Moulvibazar district [103]; chicken pox, mucus by a FMP of Jamalpur district [106]; hypotonia, scabies, leucoderma, to increase fertility in women, acne by TMPs of Santal tribe of Rangpur district [107]; jaundice, diarrhea, dysentery, small pox, gonorrhoea, eczema, sedative by FMPs of Boalia sub-district, Rajshahi district [109]; snake bite by FMPs of Bheramara area in Kushtia district [110]; jaundice, tumor, sprain, dermatitis, conjunctivitis, small pox, colic by FMPs of two villages by the Rupsha River in Bagerhat district [120].
*Cuscuta reflexa* Roxb.	Sexual diseases by the Garo tribe living in Netrakona district [19];itches in Vasu Bihar village, Bogra district [39]; gastrointestinal disorders, body pain by FMPs of Sylhet Division, Bangladesh [41]; jaundice, liver diseases, uterus and liver pain in Shitol Para village, Jhalokati district [42]; alopecia, acne, glassiness of skin by FMPs of three villages in Natore and Rajshahi districts [43]; jaundice, helminthiasis by the Garo tribe inhabiting the Madhupur forest region of Bangladesh [46]; carminative by FMPs of Noakhali district [47]; itches by FMPs of Vasu Bihar village, Bogra district [49]; fever, jaundice, to maintain good health, to keep body cool by FMPs of Station Purbo Para village, Jamalpur district [33]; stoppage of urination by FMPs of six villages in Greater Naogaon district [53]; jaundice by a FMP of Gachabari village in Tangail district [56]; female infertility, fever by FMPs of four villages in Natore and Rajshahi districts [61]; gastric troubles by FMPs of Bhola district [64]; anthrax in cattle, jaundice by the tribal healers of Oraon tribe of Sylhet district [67]; jaundice by FMPs and TMPs in the vicinity of Lawachara Forest Reserve, Moulvibazar district [72]; sexual stimulant by the Marma tribe living in Naikhongchhari, Bandarban district [75]; fever, body pain, rheumatic pain, sex stimulant by the Rakhain tribe inhabiting the Chittagong Hill Tracts region [76]; excessive bleeding following menstruation by the Santal tribe residing in Rajshahi district [77]; edema, body ache, sexual stimulant, maintain good hepatic functions, jaundice by the Tripura tribe residing in Chittagong Hill Tracts, Bangladesh [78]; hair loss by TMPs of Naik clan of Rajbongshi tribe of Moulvibazar district [84]; jaundice by TMPs of the Tripura tribe residing in Comilla district [90]; diabetes by TMPs of the Harbang clan of the Tripura tribe of Mirsharai area, Chittagong district [95]; to stop bleeding from wounds, jaundice by FMPs of Dhamrai sub-district, Dhaka district [97]; jaundice by FMPs of Barisal Town, Barisal district [98]; aphrodisiac, diabetes by TMPs of Tonchongya tribe of Roangchaari Upazila in Bandarban district [99]; abdominal pain, helminthiasis, skin diseases by TMPs of the Manipuri tribe in Kamalganj Upazila, Moulvibazar district [101]; fever, jaundice by TMPs of the Bauri tribal community of Moulvibazar district [102]; low sperm count, jaundice by a FMP of Sreemangal Upazila in Maulvibazar district [105]; heart disorders by Christians living in Mirzapur village of Dinajpur ditrict, Bangladesh [108]; indigestion in Rampal sub-district of Bagerhat district [113].
*Cynodon dactylon* (L.) Pers.	Cuts and wounds by the Garo tribe living in Netrakona district [19];yellowish coloration of urine, bleeding from cuts and wounds in two villages of Rajshahi district [38]; to stop bleeding in Vasu Bihar village, Bogra district [39]; cuts and wounds, infections by FMPs of Sylhet Division, Bangladesh [41]; to stop bleeding in Shitol Para village, Jhalokati district [42]; leucorrhea, gonorrhea, diabetes, stop bleeding, infertility by FMPs of three villages in Natore and Rajshahi districts [43]; to stop bleeding by FMPs of Daudkandi sub-district of Comilla district [44]; piles by FMPs of Noakhali district [47]; astringent, tonsillitis, dermatitis, inflammation, laxative by FMPs in villages by the Bangali River of Bogra district [48]; external wound, kidney and gall bladder stones by FMPs in villages by the Padma River of Rajshahi district [48]; to stop bleeding by FMPs of Vasu Bihar village, Bogra district [49]; chronic dysentery, to keep body healthy, to stop bleeding by FMPs of Station Purbo Para village, Jamalpur district [33]; skin diseases, less urination by FMPs of Daulatdia Ghat, Kushtia district [51]; wounds, acne by FMPs of Vitbilia village in Pabna district [52]; excessive bleeding from the ovary, anemia, vomiting, stomach pain, infection of the uterus by FMPs of six villages in Greater Naogaon district [53]; sprain, cuts and wounds by FMPs of a village in Narayanganj district [55]; cuts and wounds, infected wounds by a FMP of Gachabari village in Tangail district [56]; whitish discharge in urine of women, gastric p[roblems, sexual weakness, bleeding from external cuts and wounds by a FMP of Savar in Dhaka district [60]; to stop bleeding from cuts and wounds by FMPs of four villages in Natore and Rajshahi districts [61]; to stop bleeding from cuts and wounds by FMPs of Bhola district [64]; blood dysentery by a FMP of Jhalokathi in Barisal district [65]; infections, bleeding while pregnant by FMPs of Chuadanga district [66]; headache, infection, erectile dysfunction by the tribal healers of Oraon tribe of Sylhet district [67]; lesions on tongue by the TMPs of the Rai Clan of the Tipra tribe of Sylhet Division [68]; to stop bleeding by TMPs of two Marma tribal communities in two villages of Khagrachhari district [69]; vomiting by TMPs and FMPs practicing within a Khasia tribal community in Jaflong area, Sylhet district [70]; to stop bleeding from cuts and wounds by FMPs and TMPs in the vicinity of Lawachara Forest Reserve, Moulvibazar district [72]; coughs, cuts and wounds by FMPs of Badarganj and Shekhertek villages in Rangpur district [79]; loss of libido, provides a feeling of satisfaction, biliary/hepatic disorders, thirst, vomiting, burning sensations in the body, blood purifier, coughs, fainting, loss of appetite by FMPs of three villages in Sreepur Upazilla, Magura district [80]; bleeding from external cuts and wounds by FMPs of several areas of Faridpur and Rajbari districts [83]; stomach infections, infections of uterus, to stop bleeding from external cuts and wounds by TMPs of Rai Kshatriya tribe of Pabna district [85]; to stop bleeding by FMPs of Dhamrai sub-district, Dhaka district [97]; excessive bleeding during menstruation by FMPs of Barisal Town, Barisal district [98]; cuts and wounds by TMPs of the Manipuri tribe in Kamalganj Upazila, Moulvibazar district [101]; jaundice by TMPs of the Bauri tribal community of Moulvibazar district [102]; chicken pox by a FMP of Jamalpur district [106]; tonsillitis, astringent, snake bite, dog bite by TMPs of Santal tribe of Rangpur district [107]; diabetes by the Santal tribe residing in Thakurgaon district [111]; bleeding through the nose and mouth and passing of blood with urine by TMPs of the Hodi tribe in Sherpur district [112]; physical weakness by a TMP of the Sardar (Dhangor) community in Chuadanga district [116];.
*Datura metel* L.	Mental disorders by the Garo tribe living in Netrakona district [19]; pain in two villages of Rajshahi district [38]; paralysis in Faridpur district [40]; body ache by FMPs of Sylhet Division, Bangladesh [41]; head lice, pain and swelling in breasts of females in Shitol Para village, Jhalokati district [42]; rheumatoid arthritis, anthelmintic, carminative, acne, impotency, antidote to poison by FMPs of three villages in Natore and Rajshahi districts [43]; joint pain, pain in leg by FMPs of Dinajpur district [45]; head lice infestation by FMPs of Balidha village, Jessore district [50]; dog bite, helminthiasis by FMPs of Shetabganj village, Dinajpur district [32]; swelling and pain, excessive breathing, to enlarge pupil of eye, swelling of gums and base of ears, breast pain by FMPs of Daulatdia Ghat, Kushtia district [51]; body pain by FMPs of Vitbilia village in Pabna district [52]; respiratory difficulties by FMPs of six villages in Greater Naogaon district [53]; joint pain by FMPs of seven villages in Ishwardi Upazilla, Pabna district [54]; skin diseases, dandruff by FMPs of a village in Narayanganj district [55]; cuts and wounds (to stop bleeding) by tribal medicinal practitioners (TMPs) of the Chakma tribe residing in Rangamati district [57]; dog bite, helminthiasis, elephantiasis by FMPs of three villages in Kurigram district [58]; body irritation by FMPs of four villages in Natore and Rajshahi districts [61]; mucus, pain, insanity by TMPs of 15 clans of the Garo tribe of Madhupur, Tangail district [63]; rheumatism by FMP from Jhenaidah district [66]; intestinal dysfunction, wounds, paralysis by the tribal healers of Oraon tribe of Sylhet district [67]; whole body pain by the TMPs of the Rai Clan of the Tipra tribe of Sylhet Division [68]; paralysis by a FMP of a village in Faridpur district [40]; pus in ears by a Pahan TMP in Dinajpur district [71]; abscess by FMPs of three areas in Pirojpur district [74]; throat pain in children by the Santal tribe residing in Rajshahi district [77]; sudden insanity by FMPs of Badarganj and Shekhertek villages in Rangpur district [79]; antidote to poisoning by FMPs of Terbaria and Babla villages in Tangail district [81];]; pain, ear ache, paralysis by TMPs of Bongshi tribe in Tangail district [82]; being possessed by ‘ghosts’ by TMPs of Rai Kshatriya tribe of Pabna district [85]; allergy, asthma by TMPs of the Tripura tribe residing in Comilla district [90]; abscess, shrinking of pupils in the eyes, swelling of ear lobes by a FMP practicing among tea garden workers in Sreemangal, Maulvibazar district [92]; scabies, eczema, allergy by FMPs of Barisal Town, Barisal district [98]; cough, headache, dizziness, bloating by TMPs of the Harbang clan of the Tripura tribe of Mirsharai area, Chittagong district [95]; asthma, pain in eyes, insanity by Christians living in Mirzapur village of Dinajpur ditrict, Bangladesh [108]; joint pain by TMPs of the Sigibe clan of the Khumi tribe of Thanchi sub-district in Bandarban district [115]; hypotonic, helminthiasis, snake bite, sedative, anti-spasmodic, burn by FMPs of two villages by the Rupsha River in Bagerhat district [120].
*Delonix regia* (Bojer) Raf.	To increase sexual energy by the tribal healers of Oraon tribe of Sylhet district [67].
*Dillenia indica* L.	Sex stimulant by FMPs of Daudkandi sub-district of Comilla district [44]; dysentery by FMPs of Dinajpur district [45]; loss of appetite, to prevent stomach upsets, diarrhea, dysentery by FMPs of a village in Narayanganj district [55]; dysentery, sexually transmitted diseases by FMPs of three villages in Kurigram district [58]; indigestion, loss of appetite by a FMP of a village in Faridpur district [40]; to enhance digestion by FMPs of three areas in Pirojpur district [74]; edema, abscess, appetite stimulant by the Tripura tribe residing in Chittagong Hill Tracts, Bangladesh [78]; hydrocele, contraceptive by FMPs of Barisal Town, Barisal district [98]; diarrhea by TMPs of the Bauri tribal community of Moulvibazar district [102].
*Diospyros peregrina* Gürke	Mucus with stool by FMPs in villages by the Padma River of Rajshahi district [48]; constipation, anorexia by FMPs of Vasu Bihar village, Bogra district [49]; leucorrhea, thorn-induced infections, gangrene, cough, mucus, biliary diseases, blood purifier by FMPs of Daulatdia Ghat, Kushtia district [51]; oral lesions, skin infections by the TMPs of the Rai Clan of the Tipra tribe of Sylhet Division [68]; dysentery, diabetes by FMPs of three areas in Pirojpur district [74]; fever, skin problems, rheumatic pain, cold, respiratory problem by the Rakhain tribe inhabiting the Chittagong Hill Tracts region [76]; dysentery, injury by FMPs of Dhamrai sub-district, Dhaka district [97].
*Elettaria cardamomum* (L.) Maton	To increase strength, appetite stimulant by FMPs of Station Purbo Para village, Jamalpur district [33]; infertility in women by FMPs of Chuadanga district [66]; coughs, blood disorders, anti-infective, abnormal palpitation of heart, poisonous bites, rheumatism, stomatitis, gum disorders, vomiting, respiratory distress by FMPs of two villages in Bagerhat district [88]; toothache by a FMP of Jamalpur district [106]; jaundice by a TMP of the Sardar (Dhangor) community in Chuadanga district [116].
*Emblica officinalis* Gaertn.	To increase taste, jaundice, gastric problems, indigestion by the Garo tribe living in Netrakona district [19]; tooth pain in Vasu Bihar village, Bogra district [39]; loss of appetite in Faridpur district [40]; appetite stimulant, indigestion by FMPs of Sylhet Division, Bangladesh [41]; burning sensations in urinary tract, leucorrhea, hair loss, reduce graying of hair in Shitol Para village, Jhalokati district [42]; alopecia, appetizer by FMPs of three villages in Natore and Rajshahi districts [43]; loss of hair, to stop vomiting by FMPs of Daudkandi sub-district of Comilla district [44]; hair loss, indigestion, debility by FMPs of Dinajpur district [45]; tooth pain by FMPs of Vasu Bihar village, Bogra district [49]; to increase appetite, skin diseases, fever, to increase strength, burning sensations during urination, hair loss, graying of hair by FMPs of Station Purbo Para village, Jamalpur district [33]; to prevent hair loss, respiratory tract disorders, hepatic disorders, gastrointestinal disorders by FMPs of Shetabganj village, Dinajpur district [32]; to stimulate appetite by FMPs of six villages in Greater Naogaon district [53]; loss of hair, irritation during urination by FMPs of seven villages in Ishwardi Upazilla, Pabna district [54]; stomach disorders, flatulence, indigestion by a FMP of Gachabari village in Tangail district [56]; haemorrhoids, gastrointestinal disorders, ulcer, gastric pain by tribal medicinal practitioners (TMPs) of the Chakma tribe residing in Rangamati district [57]; blood purifier, anemia, hair loss, coughs, spleen disorders, gastrointestinal disorders by FMPs of three villages in Kurigram district [58]; graying of hair by TMPs of Goala tribe of Moulvibazar district [59]; hair loss by a FMP of Savar in Dhaka district [60]; to increase sexual strength, to improve appetite by TMPs of 15 clans of the Garo tribe of Madhupur, Tangail district [63]; jaundice, to keep head cool, hair loss, graying of hair by FMPs of Bhola district [64]; blood dysentery by a FMP of Jhalokathi in Barisal district [65]; intestinal dysfunction, blood purifier by the tribal healers of Oraon tribe of Sylhet district [67]; headache, conjunctivitis by the TMPs of the Rai Clan of the Tipra tribe of Sylhet Division [68]; loss of appetite by a FMP of a village in Faridpur district [40]; mucus by TMPs of two Marma tribal communities in two villages of Khagrachhari district [69]; fever by TMPs and FMPs practicing within a Khasia tribal community in Jaflong area, Sylhet district [70]; diabetes, hair loss, dandruff, to strengthen hair by a Pahan TMP in Dinajpur district [71]; hair loss, blood purifier by FMPs and TMPs in the vicinity of Lawachara Forest Reserve, Moulvibazar district [72]; to maintain good health by FMPs of three areas in Pirojpur district [74]; fever, skin problems, loss of appetite, poisonous bites of animals or insects, diabetes by the Rakhain tribe inhabiting the Chittagong Hill Tracts region [76]; long-term fever, loss of appetite, sexual stimulant by the Tripura tribe residing in Chittagong Hill Tracts, Bangladesh [78]; to increase libido by FMPs of Badarganj and Shekhertek villages in Rangpur district [79]; biliary problem, alleviation of respiratory, stomach or hepatic problem, diabetes, fatigue, thirst, burning sensations in body especially in palms of hands or soles of feet, vomiting tendency, insanity by FMPs of three villages in Sreepur Upazilla, Magura district [80]; stomach troubles, gastric problems by FMPs of several areas of Faridpur and Rajbari districts [83]; anemia by TMPs of the Pankho tribe of Bilaichari Union, Rangamati district [86]; to increase strength, to clear urine by TMPs of Rai Kshatriya tribe of Pabna district [85]; vaginitis, burning sensations by FMPs of two villages in Bagerhat district [88loss of sensitivity in skin, chronic mucus, continuous sneezing with running water from nose, loss of sensitivity in skin due to allergy, small pustules on skin of children by FMPs of Barisal Town, Barisal district [98]; aphrodisiac, energizer, fever, body ache by TMPs of Tonchongya tribe of Roangchaari Upazila in Bandarban district [99]; cardiovascular disorders by TMPs of the Bauri tribal community of Moulvibazar district [102]; bleeding from gums, loss of appetite, headache, paralysis by a TMP of the Deb barma clan of the Tripura tribe of Moulvibazar district [103]; appetizer, gonorrhoea, toothache, itch by FMPs of Boalia sub-district, Rajshahi district [109]; erectile dysfunction by FMPs of Bheramara area in Kushtia district [110]; mucus, biliary disorders, loss of appetite, prevent hair loss by the Santal tribe residing in Thakurgaon district [111]; diabetes, cardiovascular disorders, weakness of heart, hysteria, osteoporosis by a TMP of the Sardar (Dhangor) community in Chuadanga district [116]; anemia by TMPs of Mro community of Gazalia Union in Bandarbans district [118]; fistula, vitamin C deficiency, lack of appetite, diarrhoea, dysentery, hepatitis, cold by FMPs of two villages by the Rupsha River in Bagerhat district [120].
*Enydra fluctuans* Lour.	Hepatitis in Vasu Bihar village, Bogra district [39]; blood problems, leucorrhea in Shitol Para village, Jhalokati district [42]; rheumatoid arthritis, constipation, itch, impotency, appetizer, edema by FMPs of three villages in Natore and Rajshahi districts [43]; chicken pox by FMPs in villages by the Ghaghot River of Rangpur district [48]; rabies, sedative, carminative, dermatitis by FMPs in villages by the Bangali River of Bogra district [48]; hepatitis by FMPs of Vasu Bihar village, Bogra district [49]; to keep head cool, burning sensations in the body by FMPs of six villages in Greater Naogaon district [53]; physical weakness, vision problems by FMPs of seven villages in Ishwardi Upazilla, Pabna district [54]; gastric ulcer by FMPs of three areas in Pirojpur district [74]; constipation, bitter, astringent, leprosy, bile secretion disorders, respiratory tract disorders, blood purifier by FMPs of three villages in Sreepur Upazilla, Magura district [80].
*Feronia limonia* Swingle	To enhance digestion by FMPs of three areas in Pirojpur district [74].
*Ficus racemosa* L.	Diabetes by the Garo tribe living in Netrakona district [19]; dysentery by FMPs of two villages in Rajshahi district [38]; coughs, blood dysentery by FMPs of Sylhet Division, Bangladesh [41]; ‘prodor’ disease (any disease prior to, during or following menstruation) in Shitol Para village, Jhalokati district [42]; leucorrhea by FMPs of Noakhali district [47]; stomach ache by FMPs of Balidha village, Jessore district [50]; to keep healthy, diabetes by FMPs of Station Purbo Para village, Jamalpur district [33]; diabetes by FMPs of Vitbilia village in Pabna district [52]; dysentery by FMPs of six villages in Greater Naogaon district [53]; pimples, eczema, bleeding due to external cuts and wounds, burning sensations during urination by FMPs of seven villages in Ishwardi Upazilla, Pabna district [54]; coughs, mucus, diarrhea, dysentery, debility by FMPs of a village in Narayanganj district [55]; loss of sexual desire, roughness of skin, biliary disorders, coughs, blood purifier, change of color of skin as in jaundice, acne, astringent by FMPs of three villages in Sreepur Upazilla, Magura district [80]; weakness, eye diseases, diabetes by TMPs of Bongshi tribe in Tangail district [82]; jaundice by FMPs of several areas of Faridpur and Rajbari districts [83]; to induce urination, to increase strength, gonorrhea, urethritis, to increase sexual desire, roughness of skin, biliary disorders, coughs, blood purifier, change of color of skin as in jaundice, acne, astringent by FMPs of two villages in Bagerhat district [88]; diabetes by a Chakma tribal practitioner practicing among Garo and Kush tribes in Sherpur district; [93]; diabetes by FMPs of Barisal Town, Barisal district [98]; headache, small pox, flatulence, cancer, dermatitis, burn by FMPs of two villages by the Rupsha River in Bagerhat district [120].
*Hibiscus rosa-sinensis* L.	Dysentery, debility by the Garo tribe living in Netrakona district [19]; leucorrhea in two villages of Rajshahi district [38]; dysentery by FMPs of Sylhet Division, Bangladesh [41]; gonorrhoea, constipation, sex stimulant, hematemesis, amenorrhea in Shitol Para village, Jhalokati district [42]; appetizer, anti-hemorrhagic by FMPs of three villages in Natore and Rajshahi districts [43]; lack of calcium by FMPs of Daudkandi sub-district of Comilla district [44]; stomach upsets and dysentery by the Garo tribe inhabiting the Madhupur forest region of Bangladesh [46]; leucorrhea by FMPs of Noakhali district [47]; loss of appetite by FMPs of Vasu Bihar village, Bogra district [49]; menstrual irregularities by FMPs of Balidha village, Jessore district [50]; to improve hair quality, coughs, flatulency by FMPs of Shetabganj village, Dinajpur district [32]; leucorrhea, passing of blood with urine in women, badhok disease (infertility in women due to problem in uterus) by FMPs of Vitbilia village in Pabna district [52]; menstrual difficulties by FMPs of six villages in Greater Naogaon district [53]; irregular menstruation, burns, blood dysentery by FMPs of a village in Narayanganj district [55]; prolonged menstruation by a FMP of Gachabari village in Tangail district [56]; diarrhea, infections on palm of hand by tribal medicinal practitioners (TMPs) of the Chakma tribe residing in Rangamati district [57]; hair loss by TMPs of Goala tribe of Moulvibazar district [59]; general weakness, debility by FMPs of four villages in Natore and Rajshahi districts [61]; excessive bleeding, leucorrhea by TMPs of the Tudu sub-clan of the Santal tribe in Joypurhat district [62]; leucorrhea by TMPs of 15 clans of the Garo tribe of Madhupur, Tangail district [63]; infertility by FMP from Jhenaidah district [66]; premature ejaculation by the tribal healers of Oraon tribe of Sylhet district [67]; tongue lesions, conjunctivitis, puerperal fever by the TMPs of the Rai Clan of the Tipra tribe of Sylhet Division [68]; dysentery in cattle, hair loss in humans by TMPs of two Marma tribal communities in two villages of Khagrachhari district [69]; injury by a Pahan TMP in Dinajpur district [71]; blood dysentery by FMPs and TMPs in the vicinity of Lawachara Forest Reserve, Moulvibazar district [72]; to induce vomiting, irregular menstruation by FMPs of three areas in Pirojpur district [74]; cataract by the Marma tribe living in Naikhongchhari, Bandarban district [75]; conjunctivitis, helminthiasis, viral fever, any fever, rheumatic pain, diabetes by the Rakhain tribe inhabiting the Chittagong Hill Tracts region [76]; puerperal fever by the Santal tribe residing in Rajshahi district [77]; infertility in females, to prevent death of infant following birth by TMPs of Rai Kshatriya tribe of Pabna district [85]; burning sensations during urination by a Chakma TMP practicing among Garo and Kush tribes in Sherpur district; dysentery by a Garo TMP practicing among Garo and Kush tribes in Sherpur district [93]; hair loss, excessive graying of hair, loss of brightness in hair by folk herbalists in Comilla district [94]; burning sensations during urination, yellowish color of urine by TMPs of the Nag clan of the Rai Ghatual tribe in Moulvibazar district [96]; frequent urination by FMPs of Dhamrai sub-district, Dhaka district [97]; dysentery by TMPs of the Manipuri tribe in Kamalganj Upazila, Moulvibazar district [101]; being touched by ‘evil wind’ by TMPs of the Soren clan of the Santal tribe in Rajshahi district [104]; blood dysentery by a FMP of Sreemangal Upazila in Maulvibazar district [105]; menstrual disorders by FMPs of Bheramara area in Kushtia district [110]; premature ejaculation by the Santal tribe residing in Thakurgaon district [111].
*Hyptis suaveolens* (L.) Poit.	Constipation by FMPs of Sylhet Division, Bangladesh [41]; gonorrhea by the Garo tribe inhabiting the Madhupur forest region of Bangladesh [46]; constipation, dysentery, burning sensations in stomach (acidity) by FMPs of a village in Narayanganj district [53low semen density, intestinal dysfunction by the tribal healers of Oraon tribe of Sylhet district [67]; tremor, constipation, burning sensations in the hands or body by TMPs of two Marma tribal communities in two villages of Khagrachhari district [69]; weakness by FMPs and TMPs in the vicinity of Lawachara Forest Reserve, Moulvibazar district [72];]; leucorrhea in women, low sperm density in men by TMPs of Bongshi tribe in Tangail district [82]; stomach ache in children by TMPs of the Pankho tribe of Bilaichari Union, Rangamati district [86]; constipation by TMPs of the Harbang clan of the Tripura tribe of Mirsharai area, Chittagong district [95]; cooling agent, kidney disease, urinary tract infections, dysuria, laxative by TMPs of Tonchongya tribe of Roangchaari Upazila in Bandarban district [99]; tumor, rheumatism by TMPs of the Manipuri tribe in Kamalganj Upazila, Moulvibazar district [101]; physical weakness, sense of hotness in head by a TMP of the Deb barma clan of the Tripura tribe of Moulvibazar district [103]; constipation, burning sensations during urination, anemia, low sperm count by a FMP of Sreemangal Upazila in Maulvibazar district [105]; malaria, headache, insect repellent by TMPs of Santal tribe of Rangpur district [107]; liver diseases, cancer, constipation by FMPs of Boalia sub-district, Rajshahi district [109]; constipation in Paikgacha sub-district of Khulna district [113]; diabetes by TMPs of Tonchongya tribe of Bandarban district [114].
*Ipomoea aquatica* Forssk.	Galactagogue, leucorrhea in Shitol Para village, Jhalokati district [42]; rheumatic swelling by FMPs of Noakhali district [47]; snake bite, astringent, skin disorder by FMPs in villages by the Bangali River of Bogra district [48]; gall bladder stones by FMPs of Daulatdia Ghat, Kushtia district [51]; to keep head cool, burning sensations in hands, feet, head or body by FMPs of six villages in Greater Naogaon district [53]; jaundice, diarrhea, skin diseases by FMPs of a village in Narayanganj district [55]; constipation by tribal medicinal practitioners (TMPs) of the Chakma tribe residing in Rangamati district [57]; antidote to poisoning, chicken pox by FMPs of three villages in Kurigram district [58]; gonorrhea, antidote to poisoning, to increase milk of nursing mother, low sperm count, low semen volume by FMPs of Bhola district [64]; cuts and wounds by a Pahan TMP in Dinajpur district [71]; gonorrhea, low sperm count by FMPs of three areas in Pirojpur district [74]; snake bite, haemorrhoids, indigestion, burns by FMPs of two villages by the Rupsha River in Bagerhat district [120].
*Kalanchoe pinnata* (Lam.) Pers.	Gall bladder stones, bloating, to stop bleeding from cuts and wounds in Rajshahi district [38]; asthma in Vasu Bihar village, Bogra district [39]; kidney stones in Faridpur district [40]; burning sensations during urination, kidney stones by FMPs of Sylhet Division, Bangladesh [41]; diarrhea, to stop bleeding, irregular urination, burning sensations in urinary tract in Shitol Para village, Jhalokati district [42]; colic, sexual disorders, appetizer by FMPs of three villages in Natore and Rajshahi districts [43]; gastric problems by FMPs of Daudkandi sub-district of Comilla district [44]; kidney stones, any type of wounds, indigestion by FMPs of Dinajpur district [41urinary problems, kidney or gall bladder stones by FMPs of Noakhali district [47]; kidney and gall bladder stones by FMPs in villages by the Padma River of Rajshahi district [48]; asthma by FMPs of Vasu Bihar village, Bogra district [49]; stomach ache by FMPs of Balidha village, Jessore district [50]; to clarify urine, hematemesis, cough, mucus, epilepsy, stomach ache in children by FMPs of Station Purbo Para village, Jamalpur district [33]; respiratory tract disorders, hepatic disorders, gastrointestinal disorders, spleen disorders, acne by FMPs of Shetabganj village, Dinajpur district [32]; gall bladder stones, pain from piles by FMPs of Daulatdia Ghat, Kushtia district [51]; gall bladder stones by FMPs of Vitbilia village in Pabna district [52]; bloating, gastrointestinal stones, kidney stones, gastrointestinal disorders by FMPs of six villages in Greater Naogaon district [53]; diarrhea, bleeding from cuts and wounds, gall bladder stones by FMPs of seven villages in Ishwardi Upazilla, Pabna district [54]; diarrhea, cuts and wounds by FMPs of a village in Narayanganj district [55]; urinary and sexual problems in men by a FMP of Gachabari village in Tangail district [56]; constipation, diabetes, stomach or kidney stones by tribal medicinal practitioners (TMPs) of the Chakma tribe residing in Rangamati district [57]; formation of stones in the stomach, headache by FMPs of three villages in Kurigram district [58]; urinary tract infections, kidney stones by a FMP of Savar in Dhaka district [60]; indigestion, cholelithiasis by FMPs of four villages in Natore and Rajshahi districts [61]; to stop bleeding from external cuts and wounds by TMPs of 15 clans of the Garo tribe of Madhupur, Tangail district [63]; urinary disorders by FMPs of Bhola district [64]; haemorrhoids, blood dysentery, acne by a FMP of Jhalokathi in Barisal district [65]; kidney stone, stomach stone, bloating by FMPs of two villages in Natore district [31]; hotness in head, headache by FMPs of Chuadanga district [66]; dysentery, indigestion by the TMPs of the Rai Clan of the Tipra tribe of Sylhet Division [68]; kidney stones by a FMP of a village in Faridpur district [40]; long-term coughs, burning sensations in the stomach by TMPs of two Marma tribal communities in two villages of Khagrachhari district [69]; heart disorders by FMPs and TMPs in the vicinity of Lawachara Forest Reserve, Moulvibazar district [72]; excessive urination, abdominal pain, dysentery, insecticide by FMPs of three areas in Pirojpur district [74]; muscle pain, scabies, boils, rheumatism by the Marma tribe living in Naikhongchhari, Bandarban district [75];]; coughs, mucus, fever, sudden loss of consciousness (epilepsy-like), constipation, piles by the Tripura tribe residing in Chittagong Hill Tracts, Bangladesh [78];]; kidney stones by FMPs of Badarganj and Shekhertek villages in Rangpur district [79]; stone formation in stomach by FMPs of Terbaria and Babla villages in Tangail district [81]; bitter, astringent, alleviation of respiratory, stomach or hepatic problems, piles, swelling or tumor, burning sensations during urination, kidney or gall bladder stones, spleen disorders, urinary problems arising from diabetes or other endocrinological disorders, vaginal diseases by FMPs of three villages in Sreepur Upazilla, Magura district [80]; alleviation or prevention of respiratory, stomach and hepatic problems, piles, swelling or tumor, burning sensations during urination, kidney or gall bladder stones, spleen disorders, urinary problems arising from endocrinological disorders like diabetes, vaginal diseases, urinary disorders, vaginitis, insufficient sperm count by FMPs of two villages in Bagerhat district [88]; stomach disorders by TMPs of the Khatriya and Kashya clans of the Bagdi tribe in Rajbari district [89]; kidney stones by TMPs of the Tripura tribe residing in Comilla district [90]; burning sensations during urination by a Chakma TMP practicing among Garo and Kush tribes in Sherpur district; typhoid by a Chakma TMP practicing among Garo and Kush tribes in Sherpur district [93]; burning sensations in thge stomach, cough and mucus in children by TMPs of the Harbang clan of the Tripura tribe of Mirsharai area, Chittagong district [95]; pain, boils, abscess, rheumatism, eczema by TMPs of Tonchongya tribe of Roangchaari Upazila in Bandarban district [99]; kidney or gall bladder stone, hypertension, cholera by TMPs of Chakma tribe of Rangapanir Chara Area in Khagrachaari district [34]; cold, dysentery, diabetes, heart diseases by TMPs of the Manipuri tribe in Kamalganj Upazila, Moulvibazar district [101]; spermatorrhea by a FMP of Sreemangal Upazila in Maulvibazar district [105]; kidney and gall bladder stones by Christians living in Mirzapur village of Dinajpur ditrict, Bangladesh [108]; headache, asthma, stone formation in any part of the body by FMPs of Boalia sub-district, Rajshahi district [109]; kidney stones by FMPs of Bheramara area in Kushtia district [110]; diarrhea in Rampal sub-district of Bagerhat district [113]; asthma by TMPs of Mro community of Gazalia Union in Bandarbans district [118].
*Lannea coromandelica* (Houtt.) Merr.	Seminal problems by the Garo tribe inhabiting the Madhupur forest region of Bangladesh [46]; puerperal fever by TMPs of the Murmu tribal community residing in Rajshahi district [87].
*Mangifera indica* L.	Influenza, helminthiasis by the Garo tribe living in Netrakona district [19]; stomach pain by FMPs of two villages in Rajshahi district [38]; diabetes, dandruff, cracking of soles of feet by FMPs of Sylhet Division, Bangladesh [41]; impotency, diabetes, eye disorders by FMPs of three villages in Natore and Rajshahi districts [43]; dysentery by the Garo tribe inhabiting the Madhupur forest region of Bangladesh [46]; jaundice, carminative, dermatitis, lack of appetite, malaria, syphilis by FMPs in villages by the Ghaghot River of Rangpur district [48]; dysentery, to prevent graying of hair by FMPs of Balidha village, Jessore district [50]; dysentery, headache by FMPs of Station Purbo Para village, Jamalpur district [33]; diarrhea, cracked soles of feet by FMPs of Shetabganj village, Dinajpur district [32]; diabetes by FMPs of Daulatdia Ghat, Kushtia district [51]; diarrhoea, dysentery by FMPs of six villages in Greater Naogaon district [53]; toothache, tooth infections, dysentery by FMPs of a village in Narayanganj district [55]; tooth ache by a FMP of Gachabari village in Tangail district [56]; dysentery, passing of blood with urine by FMPs of three villages in Kurigram district [58]; to reduce fat in the body by a FMP of Savar in Dhaka district [60]; lung pain, gastric troubles by TMPs of 15 clans of the Garo tribe of Madhupur, Tangail district [63]; diabetes, sexual weakness by FMPs of Bhola district [64]; puerperal fever by the TMPs of the Rai Clan of the Tipra tribe of Sylhet Division [68]; gastric problems by TMPs of two Marma tribal communities in two villages of Khagrachhari district [69]; jaundice by FMPs of Badarganj and Shekhertek villages in Rangpur district [79]; stomach pain by FMPs of several areas of Faridpur and Rajbari districts [83]; constipation by TMPs of Naik clan of Rajbongshi tribe of Moulvibazar district [84]; jaundice by TMPs of the Tripura tribe residing in Comilla district [90]; diabetes by a Chakma tribal practitioner practicing among Garo and Kush tribes in Sherpur district; [93]; piles, jaundice by folk herbalists in Comilla district [94]; stomach ache, cuts and wounds, meho (endocrinological disorder, diabetes) by FMPs of Barisal Town, Barisal district [98]; bloating, sex stimulant by a FMP of Sreemangal Upazila in Maulvibazar district [105]; eye diseases, antidote to poison, edema, cholera, dysentery, diabetes by FMPs of Boalia sub-district, Rajshahi district [109]; anti-inflammatory, jaundice itch by TMPs of Santal tribe of Rangpur district [112]; diarrhea by TMPs of Mro community of Gazalia Union in Bandarbans district [118].
*Mikania cordata* (Burm.f.) B.L.Rob.	To stop bleeding from cuts and wounds by FMPs of Sylhet Division, Bangladesh [41]; to stop bleeding from cuts and wounds in Shitol Para village, Jhalokati district [42]; skin diseases by FMPs of Dinajpur district [45]; gastric pain, ulcer, fresh wounds and cuts by the Garo tribe inhabiting the Madhupur forest region of Bangladesh [46]; dysentery, gastric ulcer, diabetes, cuts and wounds to stop bleeding by FMPs of Noakhali district [47]; diabetes, skin disorder by FMPs in villages by the Bangali River of Bogra district [48]; to provide a cooling effect by FMPs of Balidha village, Jessore district [50]; cuts and wounds (to stop bleeding) by FMPs of Vitbilia village in Pabna district [52]; cuts and wounds by FMPs of a village in Narayanganj district [55]; gastric troubles by a FMP of Gachabari village in Tangail district [56]; blood dysentery, blood coming out of anus by a FMP of Savar in Dhaka district [60]; bleeding from external cuts and wounds by TMPs of 15 clans of the Garo tribe of Madhupur, Tangail district [63]; gastric problems by the tribal healers of Oraon tribe of Sylhet district [67]; gastric problems by TMPs and FMPs practicing within a Khasia tribal community in Jaflong area, Sylhet district [70]; to stop bleeding from cuts and wounds by the Marma tribe living in Naikhongchhari, Bandarban district [75]; stop bleeding from wounds, astringent, chest pain after eating, acidity, dysentery by the Tripura tribe residing in Chittagong Hill Tracts, Bangladesh [78]; cuts and wounds by FMPs of Badarganj and Shekhertek villages in Rangpur district [79]; to stop bleeding from cuts and wounds by FMPs of three villages in Sreepur Upazilla, Magura district [80]; bleeding from external cuts and wounds by TMPs of Naik clan of Rajbongshi tribe of Moulvibazar district [84]; to stop bleeding from cuts and wounds by TMPs of the Tripura tribe residing in Comilla district [90]; cuts, wounds, stomach ache by TMPs of the Harbang clan of the Tripura tribe of Mirsharai area, Chittagong district [95]; bloating, stomach pain, helminthiasis, sprain, fracture by FMPs of Dhamrai sub-district, Dhaka district [97]; cuts and wounds, ulcer by FMPs of Barisal Town, Barisal district [98]; to stop bleeding from cuts and wounds by TMPs of the Bauri tribal community of Moulvibazar district [102]; cuts and wounds, dengue fever by the Santal tribe residing in Thakurgaon district [111]; bleeding from external cuts and wounds by TMPs of Tonchongya tribe of Bandarban district [114]; to stop bleeding from cuts and wounds by TMPs of Khasia tribe in several sub-districts in Sylhet district [117].
*Mimosa pudica* L.	Gynecological problems, sex stimulant by the Garo tribe living in Netrakona district [19]; pain in body, head and teeth by FMPs of Sylhet Division, Bangladesh [41]; dental pain, gingivitis in Shitol Para village, Jhalokati district [42]; impotency, appetizer, spleen enlargement by FMPs of three villages in Natore and Rajshahi districts [43]; jaundice by FMPs of Daudkandi sub-district of Comilla district [44]; rheumatic pain by FMPs of Dinajpur district [45]; loss of urinary control by FMPs in villages by the Padma River of Rajshahi district [48]; to increase sexual power, to expedite delivery, piles, wounds, chronic dysentery, prevent decaying of gums, pus in ears by FMPs of Station Purbo Para village, Jamalpur district [33]; coughs, gall bladder disorders, hematemesis by FMPs of Shetabganj village, Dinajpur district [32]; tooth diseases by FMPs of six villages in Greater Naogaon district [53]; jaundice, skin diseases by FMPs of a village in Narayanganj district [55]; leg infections especially between the fingers, conjunctivitis, burning sensations in eyes, to expedite delivery by a FMP of Gachabari village in Tangail district [56]; passing of blood during urination, burning sensations in urinary tract by tribal medicinal practitioners (TMPs) of the Chakma tribe residing in Rangamati district [57]; to increase sexual strength, jaundice by TMPs of 15 clans of the Garo tribe of Madhupur, Tangail district [63]; impotency, appetizer, spleen enlargement by the tribal healers of Oraon tribe of Sylhet district [67]; piles by the TMPs of the Rai Clan of the Tipra tribe of Sylhet Division [68]; burning sensations in hands or feet by TMPs of two Marma tribal communities in two villages of Khagrachhari district [69]; burning sensations in hands or feet by a Pahan TMP in Dinajpur district [71]; eczema, scabies, abscesses by the Marma tribe living in Naikhongchhari, Bandarban district [75]; impotency, aphrodisiac, coughs, gall bladder problems, vaginitis by FMPs of two villages in Bagerhat district [88]; problems during childbirth by a FMP practicing among tea garden workers in Sreemangal, Maulvibazar district [92]; jaundice by folk herbalists in Comilla district [94]; rheumatism, insect repellent by TMPs of the Soren clan of the Santal tribe in Rajshahi district [104]; to expedite delivery by a FMP of Sreemangal Upazila in Maulvibazar district [105]; plague, edema, elephantiasis, epilepsy by Christians living in Mirzapur village of Dinajpur ditrict, Bangladesh [108]; diarrhea, hypertension, antidote to poison by FMPs of Boalia sub-district, Rajshahi district [109]; swelling due to injury by TMPs of the Sigibe clan of the Khumi tribe of Thanchi sub-district in Bandarban district [115]; blood purifier, skin infections by a FMP of Jamalpur district [116].
*Moringa oleifera* Lam.	Diabetes by the Garo tribe living in Netrakona district [19]; body pain and fever in two villages of Rajshahi district [38]; helminthiasis in Vasu Bihar village, Bogra district [39]; sex stimulant, headache, coughs, mucus by FMPs of Sylhet Division, Bangladesh [41]; appetite stimulant, carminative, heart disorders, rheumatic fever, paralysis, liver pain, to increase bile secretion, sex stimulant, contraceptive in Shitol Para village, Jhalokati district [42]; hypertension, rheumatoid arthritis, leprosy, conjunctivitis, pain by FMPs of three villages in Natore and Rajshahi districts [43]; sterility by FMPs of Daudkandi sub-district of Comilla district [44]; chicken pox, body pain by FMPs of Dinajpur district [45]; nasal catarrh, decreased eyesight, bone fracture, sores by the Garo tribe inhabiting the Madhupur forest region of Bangladesh [46]; contraceptive, gout by FMPs in villages by the Padma River of Rajshahi district [48]; helminthiasis by FMPs of Vasu Bihar village, Bogra district [49]; rheumatism, ear disease, headache by FMPs of Balidha village, Jessore district [50]; diabetes, acidity, hypertension by FMPs of Station Purbo Para village, Jamalpur district [33]; paralysis, body pain by FMPs of six villages in Greater Naogaon district [53]; hypertension, swelling of gums, malnutrition by FMPs of three villages in Kurigram district [58]; constipation, liver problems, joint pain by a FMP of Savar in Dhaka district [60]; indigestion by FMPs of four villages in Natore and Rajshahi districts [61]; body pain, fever by FMPs of two villages in Rajshahi district [70]; piles by FMPs of three areas in Pirojpur district [74]; to stimulate appetite, roughness of skin, to increase sperm, helminthiasis, obesity, coughs, restless feeling, bloating, swelling due to injury, formation of blood clots on skin, good for eyes, goiter, pain, headache by FMPs of three villages in Sreepur Upazilla, Magura district [80]; hepatitis, jaundice by FMPs of several areas of Faridpur and Rajbari districts [83]; to break water during childbirth, diabetes by TMPs of Rai Kshatriya tribe of Pabna district [85]; to increase appetite, roughness of skin, pain, to increase sperm, acne, helminthiasis, obesity, coughs, restless feeling, bloating, swelling due to injury, formation of blood clots on skin, goiter, good for eyes, headache by FMPs of two villages in Bagerhat district [88]; rheumatic pain by a Garo TMP practicing among Garo and Kush tribes in Sherpur district; tumor by a Chakma TMP practicing among Garo and Kush tribes in Sherpur district [93]; joint pain, weakness by TMPs of the Bauri tribal community of Moulvibazar district [102]; jaundice by a TMP of the Deb barma clan of the Tripura tribe of Moulvibazar district [103]; constipation, epilepsy, abortifacient, skin eruptions, leucoderma by TMPs of Santal tribe of Rangpur district [107]; paralysis by Christians living in Mirzapur village of Dinajpur ditrict, Bangladesh [108]; cancer, night blindness, rheumatoid arthritis, helminthiasis by FMPs of Boalia sub-district, Rajshahi district [109].
*Musa paradisiaca* L.	Anemia, hematemesis, debility, dysentery by FMPs of Sylhet Division, Bangladesh [41]; excessive bleeding during childbirth by FMPs of three villages in Kurigram district [58]; eczema by a FMP of Savar in Dhaka district [60]; chronic dysentery by FMPs of three areas in Pirojpur district [74]; fever with shivering, waist pain, insanity by FMPs of several areas of Faridpur and Rajbari districts [83]; dysentery by FMPs of Barisal Town, Barisal district [98].
*Nyctanthes arbor-tristis* L.	Constipation in children, fever by the Garo tribe living in Netrakona district [19]; liver disorders in Faridpur district [40]; arthritis, malaria, expectorant in Shitol Para village, Jhalokati district [42]; fever by FMPs of Daudkandi sub-district of Comilla district [44]; fever, rheumatism, mucus by FMPs of Noakhali district, coughs, fever by FMPs of Feni district [47]; cough by FMPs of Balidha village, Jessore district [50]; rheumatism, pitto-jor (fever due to metabolic disorders) by FMPs of Daulatdia Ghat, Kushtia district [51]; skin diseases by tribal medicinal practitioners (TMPs) of the Chakma tribe residing in Rangamati district [57]; fever including dengue and malarial fever, helminthiasis by a FMP of Savar in Dhaka district [60]; bilious fever, fever by TMPs of 15 clans of the Garo tribe of Madhupur, Tangail district [63]; stomach pain by FMP from Jhenaidah district [66]; hair loss, helminthiasis by the TMPs of the Rai Clan of the Tipra tribe of Sylhet Division [68]; liver disorders by a FMP of a village in Faridpur district [40]; malaria, helminthiasis by FMPs of three areas in Pirojpur district [74]; pitto-jor (fever due to metabolic imbalances in body), fever by FMPs of Terbaria and Babla villages in Tangail district [81]; bitter, rheumatism, tuberculosis, fever by FMPs of three villages in Sreepur Upazilla, Magura district [80]; chronic fever by FMPs of three villages in Kurigram district [84]; fever by TMPs of the Pankho tribe of Bilaichari Union, Rangamati district [86]; bitter, tuberculosis, rheumatism, fever by FMPs of two villages in Bagerhat district [88]; fever by FMPs of Barisal Town, Barisal district [98]; migraine by Christians living in Mirzapur village of Dinajpur ditrict, Bangladesh [108].
*Ocimum tenuiflorum* L.	Cough, fever, bronchitis, diabetes, indigestion by the Garo tribe living in Netrakona district [19]; cold in two villages of Rajshahi district [38]; fever in Vasu Bihar village, Bogra district [39]; coughs, asthma, respiratory difficulties, mucus, leucorrhea by FMPs of Dinajpur district [45]; coughs, mucus, asthma by FMPs of Noakhali district [47]; fever by FMPs of Vasu Bihar village, Bogra district [49]; cough, mucus, asthma by FMPs of Station Purbo Para village, Jamalpur district [33]; contraceptive, immunity enhancer by FMPs of Daulatdia Ghat, Kushtia district [51]; coughs, cold, fever by FMPs of Vitbilia village in Pabna district [52]; meho (endocrinological disorders, diabetes), coughs by FMPs of six villages in Greater Naogaon district [53]; cold, skin disease, toothache by FMPs of seven villages in Ishwardi Upazilla, Pabna district [54]; mucus, cough, respiratory difficulties, sudden fits of vomiting, fever, headache by a FMP of Gachabari village in Tangail district [56]; coughs, respiratory difficulties, fever, diabetes, skin diseases by tribal medicinal practitioners (TMPs) of the Chakma tribe residing in Rangamati district [57]; asthma, tuberculosis, coughs, mucus, itches by FMPs of three villages in Kurigram district [58]; cold, coughs, bronchitis, pneumonia by FMPs of four villages in Natore and Rajshahi districts [61]; mucus, coughs by TMPs of 15 clans of the Garo tribe of Madhupur, Tangail district [63]; coughs, mucus, respiratory difficulties by FMPs of Bhola district [64]; allergy by a FMP of Jhalokathi in Barisal district [65]; coughs by the tribal healers of Gor tribe of Sylhet district [67]; coughs by TMPs of two Marma tribal communities in two villages of Khagrachhari district [69]; coughs in children, hoarseness of voice by a Pahan TMP in Dinajpur district [71]; coughs by FMPs and TMPs in the vicinity of Lawachara Forest Reserve, Moulvibazar district [72]; dry cough by FMPs of three areas in Pirojpur district [74]; feeling of restlessness, excessive sexual desire, burning sensations in body especially in palms of hands or soles of feet, increases bile secretion, leprosy, less urination, blood purifier, piles, coughs, carminative by FMPs of three villages in Sreepur Upazilla, Magura district [80]; respiratory difficulties in children due to catching cold by TMPs of Bongshi tribe in Tangail district [82]; if infant does not drink milk or cries incessantly by TMPs of the Pankho tribe of Bilaichari Union, Rangamati district [86]; coughs by TMPs of the Tripura tribe residing in Comilla district [90]; if infant refuses to take milk from nursing mother and cries incessantly by a Tonchongya tribal healer of Rangamati district [91]; fever by a Chakma tribal practitioner practicing among Garo and Kush tribes in Sherpur district; rheumatic problems, gastric problems by a Kush tribal practitioner practicing among Garo and Kush tribes in Sherpur district [93]; coughs, acne by folk herbalists in Comilla district [94]; fever, loss of appetite by TMPs of the Nag clan of the Rai Ghatual tribe in Moulvibazar district [96]; coughs, mucus by FMPs of Dhamrai sub-district, Dhaka district [97]; coughs, cold by TMPs of Tonchongya tribe of Roangchaari Upazila in Bandarban district [99]; coughs by TMPs of the Manipuri tribe in Kamalganj Upazila, Moulvibazar district [101]; asthma by TMPs of the Bauri tribal community of Moulvibazar district [102]; cold by a FMP of Sreemangal Upazila in Maulvibazar district [105]; coughs, mucus by the Teli clan of the Telegu tribe of Maulvibazar district [100]; bronchitis, liver diseases, cancer by FMPs of Boalia sub-district, Rajshahi district [109]; malaria, erectile dysfunction, coughs, colds by FMPs of Bheramara area in Kushtia district [110]; coughs, mucus, uneasy feeling in the body by the Santal tribe residing in Thakurgaon district [111].
*Phoenix sylvestris* (L.) Roxb.	To expedite delivery in pregnant women by FMPs of six villages in Greater Naogaon district [53]; helminthiasis, biliary problems, diuretic, acne by TMPs of Santal tribe of Rangpur district [107].
*Piper betel* Blanco	Bronchitis, antidote to poison, indigestion by the Garo tribe living in Netrakona district [19]; to remove head lice, boils by FMPs of Sylhet Division, Bangladesh [41]; swelling of hand, leg, nose or skin, flatulence or dyspepsia, aphrodisiac in Shitol Para village, Jhalokati district [42]; stomach disorders by FMPs of Station Purbo Para village, Jamalpur district [33]; tuberculosis, coughs, meho (endocrinological disorders, diabetes) by FMPs of six villages in Greater Naogaon district [53]; external cuts and wounds, minor infections by a FMP of Gachabari village in Tangail district [56]; constipation by a FMP of Savar in Dhaka district [60]; digestive, to remove foul odor from mouth, constipation by FMPs of Bhola district [64]; headache by a FMP of Jhalokathi in Barisal district [65]; gastric problems, piles by FMPs of Chuadanga district [66]; stomach pain in expecting mother by FMP from Jhenaidah district [66]; respiratory difficulties in children by the TMPs of the Rai Clan of the Tipra tribe of Sylhet Division [68]; cold, small size of penis, fever, penile disorders by FMPs of two villages in Rajshahi district [70]; energy enhancer by FMPs of three areas in Pirojpur district [74]; lesions on the face by FMPs of several areas of Faridpur and Rajbari districts [83]; to increase memory, premature ejaculation by FMPs of two villages in Bagerhat district [88]; sex stimulant, to stop bleeding by FMPs of Dhamrai sub-district, Dhaka district [97]; cuts and wounds, helminthiasis by TMPs of the Manipuri tribe in Kamalganj Upazila, Moulvibazar district [101]; being touched by ‘evil wind’ by TMPs of the Soren clan of the Santal tribe in Rajshahi district [104]; to stop bleeding from external cuts and wounds by a FMP of Sreemangal Upazila in Maulvibazar district [105]; toothache by a FMP of Jamalpur district [106].
*Psidium guajava* L.	Toothache, acne, diabetes by the Garo tribe living in Netrakona district [19]; toothache, diarrhea by FMPs of Sylhet Division, Bangladesh [41]; dental pain, gingivitis, scabies in Shitol Para village, Jhalokati district [42]; piles by FMPs of Daudkandi sub-district of Comilla district [44]; gastric problems, cuts and wounds by FMPs of Balidha village, Jessore district [50]; dysentery by FMPs of a village in Narayanganj district [55]; flatulence, gastrointestinal disorders by tribal medicinal practitioners (TMPs) of the Chakma tribe residing in Rangamati district [57]; dysentery, puerperal fever by FMPs of three villages in Kurigram district [58]; oral hygiene by FMPs of four villages in Natore and Rajshahi districts [61]; toothache, diarrhea by the tribal healers of Oraon tribe of Sylhet district [67]; toothache by TMPs of two Marma tribal communities in two villages of Khagrachhari district [69]; toothache by a Pahan TMP in Dinajpur district [71]; dysentery, anorexia by FMPs of three areas in Pirojpur district [74];dysentery, coughs, mucus, cold, wounds, respiratory problem, maintain texture of skin, maintain normal heart condition by the Rakhain tribe inhabiting the Chittagong Hill Tracts region [76]; diarrhea, debility by FMPs of Terbaria and Babla villages in Tangail district [81]; dysentery by FMPs in villages by the Padma River of Rajshahi district [82]; to increase strength and sperm count, and appetite, fever, nutritive, piles by FMPs of two villages in Bagerhat district [88]; tooth infections, loss of appetite by a TMP of the Deb barma clan of the Tripura tribe of Moulvibazar district [103]; diabetes, toothache, carminative by TMPs of Santal tribe of Rangpur district [107].
*Punica granatum* L.	Fungal infection of the nail by the Garo tribe living in Netrakona district [19]; piles in Vasu Bihar village, Bogra district [39]; diarrhea, to increase strength by FMPs ofSylhet Division, Bangladesh [41]; menorrhagia, constipation, dysentery in Shitol Para village, Jhalokati district [42]; dysentery by FMPs of Daudkandi sub-district of Comilla district [44]; dysentery by the Garo tribe inhabiting the Madhupur forest region of Bangladesh [46]; helminthiasis, dysentery by FMPs in villages by the Ghaghot River of Rangpur district [48]; piles by FMPs of Vasu Bihar village, Bogra district [49]; blood dysentery by FMPs of Balidha village, Jessore district [50]; inflammation of nails in hand or leg, dysentery, blood dysentery, loss of appetite, heart disorders by FMPs of Station Purbo Para village, Jamalpur district [33]; menstrual problems, diarrhea, tooth infections by FMPs of Station Purbo Para village, Jamalpur district [33]; blood dysentery by FMPs of six villages in Greater Naogaon district [53]; diarrhoea by FMPs of six villages in Greater Naogaon district [53]; dysentery by FMPs of three villages in Kurigram district [58]; skin sores by a FMP of Savar in Dhaka district [60]; excessive bleeding by TMPs of the Tudu sub-clan of the Santal tribe in Joypurhat district [62]; puerperal fever by the TMPs of the Rai Clan of the Tipra tribe of Sylhet Division [68]; respiratory tract problems, biliary or hepatic problems, coughs, thirst, burning sensations in body especially in palms of hands or soles of feet, fever, throat diseases, to induce satisfied feeling, to increase sperm, slightly astringent, to increase strength, memory, appetite, carminative by FMPs of three villages in Sreepur Upazilla, Magura district [80]; severe pain, whitish discharge from vagina, hair loss by FMPs of several areas of Faridpur and Rajbari districts [83]; diabetes, heart diseases, dysentery, stimulant, tumor by FMPs of Boalia sub-district, Rajshahi district [109]; to increase strength, debility by FMPs of Bheramara area in Kushtia district [110].
*Scoparia dulcis* L*.*	Weakness due to anemia in two villages of Rajshahi district [38]; sexual diseases, nerve disorders by FMPs of Sylhet Division, Bangladesh [41]; gall bladder stones by FMPs of Dinajpur district [45]; dysentery by the Garo tribe inhabiting the Madhupur forest region of Bangladesh [46]; diabetes, cuts and wounds, gastric ulcer, weakness, fever, coughs, bronchitis, diarrhoea, dysentery, edema, toothache, jaundice, diabetes by FMPs of Noakhali district [47]; infertility in male or female, leucorrhea, malaria, dog bite, debility, piles by FMPs in villages by the Bangali River of Bogra district [48]; debility, premature ejaculation, low sperm count by FMPs in villages by the Padma River of Rajshahi district [48]; diabetes by FMPs of Station Purbo Para village, Jamalpur district [33]; burning sensations during urination, severe fever, meho by FMPs of six villages in Greater Naogaon district [53]; gastric ulcer, anemia by FMPs of seven villages in Ishwardi Upazilla, Pabna district [54]; pain in chin or throat, tonsillitis, throat cancer, facial redness, eczema, skin diseases by tribal medicinal practitioners (TMPs) of the Chakma tribe residing in Rangamati district [57]; meho (endocrinological disorder) by FMPs of three villages in Kurigram district [58]; gastric problems, dysentery, diabetes by a FMP of Savar in Dhaka district [60]; diarrhea by FMPs of Chuadanga district [66]; dysentery by FMP from Jhenaidah district [66]; fever, cold, coughs by TMPs of two Marma tribal communities in two villages of Khagrachhari district [69]; vomiting tendency by TMPs and FMPs practicing within a Khasia tribal community in Jaflong area, Sylhet district [70]; body ache, gastric ulcer by FMPs of three areas in Pirojpur district [74]; respiratory problems and loss of appetite in children by the Marma tribe living in Naikhongchhari, Bandarban district [75]; blood dysentery by the Santal tribe residing in Rajshahi district [77]; continuous hiccups by FMPs of Badarganj and Shekhertek villages in Rangpur district [79]; urinary problems by FMPs of Terbaria and Babla villages in Tangail district [81]; diarrhea in children by TMPs of Bongshi tribe in Tangail district [82]; jaundice by TMPs of Rai Kshatriya tribe of Pabna district [85]; spermatorrhea by TMPs of the Pankho tribe of Bilaichari Union, Rangamati district [86]; physical weakness, dysentery by TMPs of the Murmu tribal community residing in Rajshahi district [87]; stomach pain in infants by a FMP practicing among tea garden workers in Sreemangal, Maulvibazar district [92]; cough in children, diarrhea by TMPs of the Harbang clan of the Tripura tribe of Mirsharai area, Chittagong district [95]; diabetes by FMPs of Dhamrai sub-district, Dhaka district [97]; dysentery in children by FMPs of Barisal Town, Barisal district [98]; snake bite, insect bite, antidote to poison by TMPs of Tonchongya tribe of Roangchaari Upazila in Bandarban district [99]; swelling of fingers by TMPs of Chakma tribe of Rangapanir Chara Area in Khagrachaari district [34]; dysentery in children by TMPs of the Manipuri tribe in Kamalganj Upazila, Moulvibazar district [101]; diabetes by the Santal tribe residing in Thakurgaon district [111]; constipation in Paikgacha sub-district of Khulna district, diarrhea, dysentery, colic in Rampal sub-district of Bagerhat district [113].
*Sesamum indicum* L.	
*Spondias pinnata* (L.f.) Kurz	Skin diseases, gastrointestinal disorders by FMPs of Sylhet Division, Bangladesh [41]; to enhance digestion by FMPs of three areas in Pirojpur district [47]; piles by FMPs of Chuadanga district [66]; blood dysentery by the TMPs of the Rai Clan of the Tipra tribe of Sylhet Division [68]; wounds, otitis, otalgia by TMPs of Tonchongya tribe of Roangchaari Upazila in Bandarban district [99].
*Streblus asper* Lour.	Appendicitis by FMPs of two villages in Rajshahi district [38]; gastrointestinal disorders, gastric problems by FMPs of Sylhet Division, Bangladesh [41]; bone disease in cattle in Shitol Para village, Jhalokati district [42]; dysuria, dysentery by the Garo tribe inhabiting the Madhupur forest region of Bangladesh [46]; fever, chicken pox, diarrhoea, dysentery, rheumatic fever, joint pain by FMPs of Noakhali district [47]; rabies by FMPs in villages by the Ghaghot River of Rangpur district [48]; calcium deficiency in bones, calcium deficiency induced disease, to keep stomach cool, to stay healthy, to increase strength by FMPs of Station Purbo Para village, Jamalpur district [33]; stomach pain by FMPs of six villages in Greater Naogaon district [53]; to increase energy by the tribal healers of Oraon tribe of Sylhet district [67]; dysentery by TMPs of two Marma tribal communities in two villages of Khagrachhari district [69]; passing of semen with urine by TMPs and FMPs practicing within a Khasia tribal community in Jaflong area, Sylhet district [70]; blood dysentery, cuts and wounds by a Pahan TMP in Dinajpur district [71]; toothpaste by FMPs and TMPs in the vicinity of Lawachara Forest Reserve, Moulvibazar district [72]; any type of tooth problem, malaria, fever with convulsions, diarrhoea by the Tripura tribe residing in Chittagong Hill Tracts, Bangladesh [78]; indigestion by FMPs of Badarganj and Shekhertek villages in Rangpur district [79]; leucorrhea in wome, low sperm density in men by TMPs of Bongshi tribe in Tangail district [82]; to prevent death of infant following birth by TMPs of Rai Kshatriya tribe of Pabna district [85]; to increase lactation in nursing mothers by TMPs of the Pankho tribe of Bilaichari Union, Rangamati district [86]; blood purifier, gall bladder problems, piles, flatulency, coughs, diarrhea by FMPs of two villages in Bagerhat district [88]; coughs, respiratory difficulties, to remove fear from mind by TMPs of the Tripura tribe residing in Comilla district [90]; tuberculosis by a FMP practicing among tea garden workers in Sreemangal, Maulvibazar district [92]; constipation, chapped skin in winter by FMPs of Dhamrai sub-district, Dhaka district [97]; rheumatism by FMPs of Barisal Town, Barisal district [98]; blood purifier by TMPs of the Soren clan of the Santal tribe in Rajshahi district [104]; elephantiasis by the Santal tribe residing in Thakurgaon district [111]; jaundice, blood in urine by TMPs of the Hodi tribe in Sherpur district [112]; ear ache by TMPs of the Sigibe clan of the Khumi tribe of Thanchi sub-district in Bandarban district [115].
*Syzygium aromaticum* (L.) Merr. & L.M. Perry	Coughs, debility, to increase mental strength by FMPs of Dinajpur district [45]; coughs, mucus by FMPs of Station Purbo Para village, Jamalpur district [33]; low semen volume, low sperm density by a FMP of Jhalokathi in Barisal district [65]; infertility in women by FMPs of Chuadanga district [66]; dry cough, stomach pain by the TMPs of the Rai Clan of the Tipra tribe of Sylhet Division [68]; coughs by TMPs of the Tripura tribe residing in Comilla district [90]; to strengthen stomach by a FMP of Jamalpur district [106].
*Syzygium cumini* (L.) Skeels	Diabetes by the Garo tribe living in Netrakona district [19]; dysentery, debility, anti-emetic, diabetes by FMPs of Sylhet Division, Bangladesh [41]; diabetes in Shitol Para village, Jhalokati district [42]; tonic, diabetes by FMPs of three villages in Natore and Rajshahi districts [43]; anemia by FMPs in villages by the Ghaghot River of Rangpur district [48]; diabetes by FMPs of Balidha village, Jessore district [50]; tooth infection, dysentery, diabetes, stone in kidney or penis by FMPs of Station Purbo Para village, Jamalpur district [33]; diabetes by FMPs of six villages in Greater Naogaon district [53]; diabetes by FMPs of seven villages in Ishwardi Upazilla, Pabna district [54]; diabetes, chronic dysentery by FMPs of three villages in Kurigram district [58]; swelling of abdomen, pain in navel by TMPs of 15 clans of the Garo tribe of Madhupur, Tangail district [63]; loss of appetite, diabetes, constipation, helmintic infections by FMPs of Bhola district [64]; diabetes by FMPs of three areas in Pirojpur district [74]; diabetes and urinary problems by the Marma tribe living in Naikhongchhari, Bandarban district [75]; inflammation, infrequent urination, burning sensation in urinary tract, fever, gastrointestinal problems, diarrhoea, blood with stool, toothache, skin disorders by the Rakhain tribe inhabiting the Chittagong Hill Tracts region [76]; low semen density by FMPs of Badarganj and Shekhertek villages in Rangpur district [79]; diabetes by FMPs of several areas of Faridpur and Rajbari districts [83]; coughs, burning sensations in the body, gall bladder problems, anemia, anti-infective by FMPs of two villages in Bagerhat district [88]; diabetes by a Chakma tribal practitioner practicing among Garo and Kush tribes in Sherpur district; [93]; suffering from urination or urinary pressure during taking a bath by FMPs of Barisal Town, Barisal district [98]; diabetes, dermatitis by TMPs of Santal tribe of Rangpur district [107]; digestive aid, rheumatoid arthritis by FMPs of Bheramara area in Kushtia district [110]; diabetes by the Santal tribe residing in Thakurgaon district [111]; hepatitis, diabetes, appetizer, burn, anemia, bloating, injury by FMPs of two villages by the Rupsha River in Bagerhat district [120].
*Tamarindus indica* L.	Fever, to stop vomiting, asthma by the Garo tribe living in Netrakona district [19]; diabetes and fever in Vasu Bihar village, Bogra district [39]; spleen problems, to reduce obesity by FMPs of Sylhet Division, Bangladesh [41]; bleeding due to piles in Shitol Para village, Jhalokati district [42]; diabetes, fever by FMPs of Vasu Bihar village, Bogra district [49]; eye diseases, cataract, rheumatism, dysentery by FMPs of Balidha village, Jessore district [50]; chronic dysentery, rheumatic pain, cold, oral lesions, burning sensations in hands or feet by FMPs of Station Purbo Para village, Jamalpur district [33]; burning sensations during urination, pain due to sprain, old infections that would not heal by FMPs of Shetabganj village, Dinajpur district [32]; to increase milk production in cows by FMPs of six villages in Greater Naogaon district [53]; constipation, loss of appetite, diarrhea, chronic fever, dysentery by FMPs of a village in Narayanganj district [55]; skin infections, burning sensations during urination by FMPs of three villages in Kurigram district [58]; uterine muscle relaxant by FMPs of four villages in Natore and Rajshahi districts [61]; premature ejaculation, bloating, indigestion, coughs, burning sensations in the body, leucoderma, acne, loss of appetite, frequent thirsts, edema, blood poisoning, pain, blood dysentery by FMPs of Bhola district [64]; edema by FMP from Jhenaidah district [66]; hypertension by FMPs of three areas in Pirojpur district [74]; burning sensations during urination by TMPs of Bongshi tribe in Tangail district [82]; increase digestion, premature ejaculation, coughs, flatulence, hypertension by FMPs of two villages in Bagerhat district [88]; diabetes by a Chakma tribal practitioner practicing among Garo and Kush tribes in Sherpur district; [93]; dysentery, burning sensations during urination by FMPs of Dhamrai sub-district, Dhaka district [97]; blood purifier, goiter by TMPs of the Soren clan of the Santal tribe in Rajshahi district [104]; conjunctivitis, burning sensations during urination, bone fracture, sprain with swelling by a FMP of Jamalpur district [106]; diabetes, anorexia, insect repellent by TMPs of Santal tribe of Rangpur district [107]; diabetes, appetizer, jaundice, eczema, conjunctivitis by FMPs of Boalia sub-district, Rajshahi district [109]; syphilis, infections within the penis, difficulties in urination, burning sensations during urination by FMPs of Bheramara area in Kushtia district [110]; gastritis, indigestion in Rampal sub-district of Bagerhat district [113]; anemia by TMPs of Mro community of Gazalia Union in Bandarbans district [118].
*Terminalia arjuna* (Roxb. ex DC.). Wight & Arn.	Heart disease, dysentery, diarrhea, jaundice by the Garo tribe living in Netrakona district [19]; heart disorders, to get protection from evil doings by FMPs of two villages in Rajshahi district [38]; heart disorders in Faridpur district [40]; cardiovascular disorders, appetite stimulant by FMPs of Sylhet Division, Bangladesh [41]; cardiovascular diseases, cholera, blood dysentery, piles with bleeding in Shitol Para village, Jhalokati district [42]; leucorrhea, rheumatoid arthritis, infertility, weakness by FMPs of three villages in Natore and Rajshahi districts [43]; sex stimulant, heart diseases by FMPs of Daudkandi sub-district of Comilla district [44]; heart diseases, rheumatism by FMPs of Dinajpur district [45]; erectile dysfunction by FMPs in villages by the Padma River of Rajshahi district [48]; heart disorders, indigestion by FMPs of Balidha village, Jessore district [50]; to increase sexual power, cough, asthma, heart disorder, dysentery by FMPs of Station Purbo Para village, Jamalpur district [33]; heart diseases by FMPs of Daulatdia Ghat, Kushtia district [51]; respiratory problems, cough, fever, debility, hypotension by FMPs of six villages in Greater Naogaon district [53]; heart disease, bone fracture by FMPs of seven villages in Ishwardi Upazilla, Pabna district [54]; dysentery, flatulence, stomach pain, indigestion by a FMP of Gachabari village in Tangail district [56]; heart disease, pain in heart, blood coming from mouth, chronic dysentery by FMPs of three villages in Kurigram district [58]; heart disorders, hepatitis, kidney problems, passing of semen with urine by FMPs of several areas of Faridpur and Rajbari districts [83]; stomach and heart disorders, graying of hair by TMPs of Goala tribe of Moulvibazar district [59]; chest pain, weak nerves, lung pain, gastric troubles by TMPs of 15 clans of the Garo tribe of Madhupur, Tangail district [63]; heart disorders, low semen density, blood purifier, coughs, leucorrhea by FMPs of Bhola district [64]; blood dysentery by a FMP of Jhalokathi in Barisal district [65]; heart disorders by FMPs of two villages in Natore district [31]; used as preventive medicine against spermatorrhea and cardiovascular disorders as well as to raise body resistance against diseases and to keep the body healthy and mind contented by FMP of Jhenidah district [66]; body pain, intestinal dysfunction by the tribal healers of Oraon tribe of Sylhet district [67]; bone pain, puerperal fever by the TMPs of the Rai Clan of the Tipra tribe of Sylhet Division [68]; heart disorders by a FMP of a village in Faridpur district [40]; heart disorders, low semen density by FMPs and TMPs in the vicinity of Lawachara Forest Reserve, Moulvibazar district [72]; abnormal heart beat by FMPs of three areas in Pirojpur district [74]; heart disorders, hepatic disorders, jaundice, maintenance of normal blood pressure by the Rakhain tribe inhabiting the Chittagong Hill Tracts region [76]; low sperm count, dysentery by FMPs of Badarganj and Shekhertek villages in Rangpur district [79]; loss of sexual desire, heart disorders, constipation, infections due to cuts and wounds, b lood purifier, obesity, diabetes, acne, coughs by FMPs of three villages in Sreepur Upazilla, Magura district [80]; spermatorrhea by TMPs of Bongshi tribe in Tangail district [82]; snake bite by TMPs of Rai Kshatriya tribe of Pabna district [85]; dysentery, flatulency by TMPs of the Murmu tribal community residing in Rajshahi district [87]; heart disorders, fluttering of heart by TMPs of the Tripura tribe residing in Comilla district [90]; any type of heart disorders by TMPs of the Harbang clan of the Tripura tribe of Mirsharai area, Chittagong district [95]; burning sensations during urination, hypertension, asthma by folk herbalists in Comilla district [94]; abnormal rhythms of heart by FMPs of Barisal Town, Barisal district [98]; sex stimulant by TMPs of Chakma tribe of Rangapanir Chara Area in Khagrachaari district [34]; heart diseases by TMPs of the Manipuri tribe in Kamalganj Upazila, Moulvibazar district [101]; cardiovascular disorders, whitish discharge during urination, burning sensations during urination, puerperal fever by TMPs of the Bauri tribal community of Moulvibazar district [102]; chest pain due to heart disorders, burning sensations during urination, bone fracture by a TMP of the Deb barma clan of the Tripura tribe of Moulvibazar district [103]; knee and waist pain by TMPs of the Soren clan of the Santal tribe in Rajshahi district [104]; heart disorders, watery eyes by a FMP of Sreemangal Upazila in Maulvibazar district [105]; pain due to injury, diabetes by a FMP of Jamalpur district [106]; hypertension, anemia, leprosy by FMPs of Boalia sub-district, Rajshahi district [109]; depression on both sides of the head and chest and appearance of yellow color in palm of hands and eyes by TMPs of the Hodi tribe in Sherpur district [112]; osteoporosis by a TMP of the Sardar (Dhangor) community in Chuadanga district [116]; heart disease, gynaecological disorders, central nervous system stimulant, leprosy, gonorrhea by FMPs of two villages by the Rupsha River in Bagerhat district [120].
*Terminalia bellirica* (Gaertn.) Roxb.	Coughs, to increase strength, appetite stimulant, to increase eye sight by FMPs of Sylhet Division, Bangladesh [41]; stimulant, impotency by FMPs of three villages in Natore and Rajshahi districts [43]; impotency, coughs, indigestion by FMPs of Dinajpur district [45]; tonic, diarrhea, dysentery, coughs, breathing problems, hair tonic, joint pain by FMPs of Noakhali district [47]; asthma, allergy, to maintain heart, lungs and liver in good condition by FMPs of Station Purbo Para village, Jamalpur district [33]; to cure any disease by FMPs of Vitbilia village in Pabna district [52]; blood purifier, appetite stimulant by FMPs of six villages in Greater Naogaon district [53]; stomach disorders, flatulence, indigestion by a FMP of Gachabari village in Tangail district [56]; abscess, burning sensations on skin, haemorrhoids by tribal medicinal practitioners (TMPs) of the Chakma tribe residing in Rangamati district [57]; coughs, spleen disorders, to clear bowels, flatulence, dysentery by FMPs of three villages in Kurigram district [58]; helminthiasis, loss of hair by the tribal healers of Oraon tribe of Sylhet district [67]; jaundice by TMPs of two Marma tribal communities in two villages of Khagrachhari district [69]; diabetes by a Pahan TMP in Dinajpur district [71]; to keep healthy by FMPs of three areas in Pirojpur district [74]; long-term fever, loss of appetite, sex stimulant by the Tripura tribe residing in Chittagong Hill Tracts, Bangladesh [78]; to increase libido, acidity by FMPs of Badarganj and Shekhertek villages in Rangpur district [79]; astringent, coughs, biliary disorders, good for eyes and hair, helminthiasis, breaking down of voice, thirst, vomiting tendency, rheumatism by FMPs of three villages in Sreepur Upazilla, Magura district [80]; gastric problems by FMPs of several areas of Faridpur and Rajbari districts [83]; constipation by TMPs of Goala tribe of Moulvibazar district [59]; anemia by TMPs of the Pankho tribe of Bilaichari Union, Rangamati district [86]; blood purifier by a FMP practicing among tea garden workers in Sreemangal, Maulvibazar district [92]; dysentery, blood dysentery, irritable bowel syndrome, gastric problems, indigestion by a Chakma tribal practitioner practicing among Garo and Kush tribes in Sherpur district; constipation by a Kush TMP practicing among Garo and Kush tribes in Sherpur district [93]; cough, mucus, asthma by TMPs of the Harbang clan of the Tripura tribe of Mirsharai area, Chittagong district [95]; aphrodisiac, energizer, fever, body ache by TMPs of Tonchongya tribe of Roangchaari Upazila in Bandarban district [99]; fever witn shivering, asthma by TMPs of the Bauri tribal community of Moulvibazar district [102]; bleeding from gums, loss of appetite, headache, paralysis by a TMP of the Deb barma clan of the Tripura tribe of Moulvibazar district [103]; skin diseases, physical weakness by a FMP of Sreemangal Upazila in Maulvibazar district [105]; constipation, sexual diseases by FMPs of Boalia sub-district, Rajshahi district [109]; erectile dysfunction by FMPs of Bheramara area in Kushtia district [110]; diabetes, low density of semen, kidney problems, cardiovascular disorders, weakness of heart, hysteria, osteoporosis by a TMP of the Sardar (Dhangor) community in Chuadanga district [116].
*Terminalia chebula* Retz.	Digestive aid, quenches thirst, blood dysentery, bloating, constipation by FMPs of Sylhet Division, Bangladesh [41]; constipation, nausea in Shitol Para village, Jhalokati district [42]; infections, indigestion by FMPs of three villages in Natore and Rajshahi districts [43]; purgative, cough relief by FMPs of Daudkandi sub-district of Comilla district [44]; bacterial diseases by FMPs of Dinajpur district [45]; stomachic by the Garo tribe inhabiting the Madhupur forest region of Bangladesh [46]; jaundice by FMPs in villages by the Ghaghot River of Rangpur district [48]; bloating, gastrointestinal disorders, stomach ache, heart disorders, debility, helminthiasis by FMPs of Station Purbo Para village, Jamalpur district [33]; constipation, less urination by FMPs of Daulatdia Ghat, Kushtia district [51]; blackening of hair, acne, acidity by FMPs of Vitbilia village in Pabna district [52]; blood purifier, appetite stimulant by FMPs of six villages in Greater Naogaon district [53]; constipation, vomiting by FMPs of seven villages in Ishwardi Upazilla, Pabna district [54]; vomiting tendency, constipation, skin diseases by FMPs of a village in Narayanganj district [55]; haemorrhoids, diabetes by tribal medicinal practitioners (TMPs) of the Chakma tribe residing in Rangamati district [57]; coughs, spleen disorders, flatulence, constipation, helminthiasis by FMPs of three villages in Kurigram district [58]; to stimulate appetite by TMPs of 15 clans of the Garo tribe of Madhupur, Tangail district [63]; hemorrhoids by a FMP of Jhalokathi in Barisal district [65]; indigestion, vomiting, constipation, intestinal dysfunction by the tribal healers of Oraon tribe of Sylhet district [67]; weakness by the TMPs of the Rai Clan of the Tipra tribe of Sylhet Division [68]; gastric problems by TMPs of two Marma tribal communities in two villages of Khagrachhari district [69]; diabetes by a Pahan TMP in Dinajpur district [71]; to keep healthy by FMPs of three areas in Pirojpur district [74]; astringent, excessive sexual desire, to increase intelligence, good for eyes, to increase longevity, respiratory problems, coughs, piles, leprosy, edema, helminthiasis, breaking down of voice, chronic dysentery, constipation, tumor or swelling, jaundice, loss of appetite, stone dissolving by FMPs of three villages in Sreepur Upazilla, Magura district [80]; gastric problems by FMPs of several areas of Faridpur and Rajbari districts [83]; anemia by TMPs of the Pankho tribe of Bilaichari Union, Rangamati district [86]; piles, asthmatic problems, coughs, fever by FMPs of two villages in Bagerhat district [88]; irritable bowel syndrome, gastric problems, indigestion by a Chakma tribal practitioner practicing among Garo and Kush tribes in Sherpur district; constipation by a Kush TMP practicing among Garo and Kush tribes in Sherpur district [93]; loss of sensitivity in skin, chronic mucus, continuous sneezing with running water from nose, loss of sensitivity in children due to allergy, small pustules on the skin of children by FMPs of Barisal Town, Barisal district [98]; aphrodisiac, energizer, fever, body ache by TMPs of Tonchongya tribe of Roangchaari Upazila in Bandarban district [99]; all kinds of diseases by TMPs of Chakma tribe of Rangapanir Chara Area in Khagrachaari district [34]; fever with shivering by TMPs of the Bauri tribal community of Moulvibazar district [102]; bleeding from gums, loss of appetite, headache, paralysis by a TMP of the Deb barma clan of the Tripura tribe of Moulvibazar district [103]; asthma, heart diseases, eye diseases, itch, night blindness by FMPs of Boalia sub-district, Rajshahi district [109]; erectile dysfunction by FMPs of Bheramara area in Kushtia district [110]; diabetes, cardiovascular disorders, weakness of heart, hysteria, osteoporosis by a TMP of the Sardar (Dhangor) community in Chuadanga district [116]; loss of appetite, malaria, hepatitis, sexual disorders, indigestion by FMPs of two villages by the Rupsha River in Bagerhat district [120].
*Zingiber officinale* Roscoe	Coughs, to reduce vomiting, gastric problems by the Garo tribe living in Netrakona district [19];]; edema, asthma, chest diseases and vomiting tendency in two villages in Rajshahi district [38]; throat pain, loss of appetite, to aid digestion, dysentery by FMPs of a village in Narayanganj district [55]; fever including dengue and malarial fever, abscess, common cold by a FMP of Savar in Dhaka district [60]; sudden sense of fear by TMPs of 15 clans of the Garo tribe of Madhupur, Tangail district [63]; haemorrhoids, blood dysentery, headache by a FMP of Jhalokathi in Barisal district [65]; bone fracture by FMPs of two villages in Natore district [31]; allergy in humans and domestic animals, infertility in women by FMPs of Chuadanga district [66]; puerperal fever, scabies by the TMPs of the Rai Clan of the Tipra tribe of Sylhet Division [68]; dysentery by a Pahan TMP in Dinajpur district [71]; arthritis, gout by FMPs of three areas in Pirojpur district [74]; vomiting tendency, fever by TMPs of Bongshi tribe in Tangail district [82]; orchitis in men, infertility in women, jaundice, rheumatic pain, passing of semen with urine and asthma in humans, bloating and foot and mouth disease in cattle by FMPs of several areas of Faridpur and Rajbari districts [83]; rheumatic pain by TMPs of Naik clan of Rajbongshi tribe of Moulvibazar district [84]; puerperal fever by TMPs of the Murmu tribal community residing in Rajshahi district [87]; bone fracture by TMPs of the Khatriya and Kashya clans of the Bagdi tribe in Rajbari district [89]; coughs, helminthiasis by TMPs of the Tripura tribe residing in Comilla district [90]; pain in leg by a FMP practicing among tea garden workers in Sreemangal, Maulvibazar district [92]; sexual weakness by a Chakma TMP practicing among Garo and Kush tribes in Sherpur district [93]; dog bite, bone fracture by folk herbalists in Comilla district [94]; to strengthen stomach, to increase sperm count and sperm density by a FMP of Jamalpur district [106]; debility, digestive aid by FMPs of Bheramara area in Kushtia district [110]; infertility with seizures by TMPs of the Hodi tribe in Sherpur district [112]; skin ulcer, gout, pain, infections, burns by TMPs of Khasia tribe in several sub-districts in Sylhet district [117].
*Ziziphus mauritiana* Lam.	Influenza, coughs, dysentery, pain by the Garo tribe living in Netrakona district [19]; chicken pox, measles by FMPs of Sylhet Division, Bangladesh [41]; dysentery with blood and mucus in Shitol Para village, Jhalokati district [42]; piles, tooth problem by FMPs of Daudkandi sub-district of Comilla district [44]; fever, flatulence, diarrhea by tribal medicinal practitioners (TMPs) of the Chakma tribe residing in Rangamati district [57]; anti-emetic, headache by FMPs of two villages in Bagerhat district [88]; hypertension, piles, dysentery, blood dysentery by TMPs of 15 clans of the Garo tribe of Madhupur, Tangail district [63]; fever by TMPs of two Marma tribal communities in two villages of Khagrachhari district [69]; fever by TMPs and FMPs practicing within a Khasia tribal community in Jaflong area, Sylhet district [70]; carminative, anorexia, diabetes, dermatitis, eye diseases by TMPs of Santal tribe of Rangpur district [107]; indigestion in Paikgacha sub-district of Khulna district [113].

The question naturally arises as to whether there are any unique uses of the various plant species used by the Garo, Hajong and Bangalee traditional medicinal practitioners. In fact, there are a number of uses which are unique to these three communities and which previously have not been reported. *A. catechu*, which was observed in the present study to be used for diarrhea and skin diseases, has been previously reported to be used for blood dysentery [31]. The various uses of *A. chinensis* described in the present study have not been reported before. *A. malaccensis* was used by the practitioners for body pain, skin diseases, ulcer, edema and jaundice. The only previously reported use of this plant was for treatment of headache [32]. The use of *A. racemosus* for treatment of epilepsy and stomach ulcers has not been reported before. *B. oleracea*, used to treat gynaecological disorders and as a tonic has no previously reported ethnomedicinal uses in Bangladesh. *C. officinalis* was used by the practitioners to treat old wound, itches, menstrual problems, stomach upset, ulcer and inflammation. The previously reported use of this plant was against ear ache, skin infections, and insect bite [33]. The use of *C. occidentalis* to treat leg pain is new.

Other hitherto unreported uses of the various plant species include use of *C. asiatica* for skin lesions; use of *C. tamala* for headache; use of *C. verum* for coughs; use of *C. quadrangularis* for stomach upset, stomach ulcer and malaria fever; use of *C. grandis* for scabies, eczema and itches; use of *C. cordifolia* for wounds; use of *C. dactylon* for diabetes; use of *D. regia* for piles and boils (the previously reported use of this plant was to increase sexual energy [34]); use of *D. indica* for fever and coughs; use of *D. peregrina* for dysentery and cholera; use of *E. cardamomum* for asthma; use of *F. limonia* for pimples; use of *I. aquatica* for piles; use of *L. coromandelica* for urinary problems and diabetes; use of *N. arbor-tristis* for constipation; use of *P. sylvestris* for nervousness, coughs and fever; use of *P. guajava* for menstrual disorders; use of *S. dulcis* for fever; use of *S. indicum* for fistula, burns associated with infection, pain and blisters; use of *S. aromaticum* for asthma; use of *S. cumini* for excessive bleeding during menstruation; and use of *T. indica* for sinusitis and chronic cold. Thus this study adds to the reported ethnomedicinal uses of the plant species mentioned, which in turn can lead scientists to perform further relevant research on the pharmacological properties of the various plant species, isolation of bioactive constituents and validating the traditional uses.

### Relevance of the findings for public health and/or environmental issues

Bangladesh is a developing country with the vast majority of people (including tribal/indigenous communities) living in rural areas with inadequate access to modern doctors and clinics. Moreover, such doctors, clinics and allopathic medicine are not affordable to these people. As such, any scientific studies carried out with medicinal plants used traditionally and involving pharmacological activity studies, isolation and identification of bioactive components, followed by clinical trials can go a long way in mitigating the sufferings of these poor illiterate communities, for these plants are still to some extent available and easily affordable. From that view point, ethnomedicinal studies like this can spur scientific interest leading to scientific validation of traditional uses of medicinal plants.

The other relevant point is such studies and findings can spur conservation efforts in preserving both plants and knowledge of their uses, for both are fast disappearing because of rapid deforestation caused from increases in population, and rural people forgetting their traditional knowledge because of the introduction of ‘city culture and habits’. Such introduction is causing the rural people to somewhat disdain their traditional way of living and culture, considering these as ‘primitive’ and not fit for the modern age. Plants have always formed a good source for many efficacious allopathic medicines and thorough documentation of traditional ways of using medicinal plants to cure various diseases can provide a modern day scientist with important research material and ideas to conduct relevant disease-alleviating research.

## Conclusion

The three communities, namely Garo, Hajong and Bangalee of Garo Hills heavily depend on the ethnopharmacological remedies for primary health care, especially fever, cold, coughs, headache, body pain, diarrhea, dysentery, constipation, indigestion, wounds, boils, skin diseases, helminthiasis, and urinary troubles. One of the important finding from this study reveals that the THPs never considered the importance of the preservation and documentation of their knowledge. The focus group discussion and personal interviews reflects the reluctance of the young generation towards their native ethnobotanical practice. The present study provides an overview of the medicinal plant usage in Durgapur Garo Hills area. The current investigation identified a total of 71 plant species used for 82 different ailments, which can be further subdivided in 16 major ailment categories. Extensive use of plants to manage dermatological (25 species) and gastrointestinal disorders (36 species) signifies that these two diseases are quite widespread in the study area. Unplanned urbanisation is adversely affecting the natural habitat of numerous plant species with important medicinal values. Inclination towards modernisation is creating a negative attitude towards the age old practice of ethnobotanical medicine, whereas, prescribing allopathic medicine by non-professionals is putting the health system at risk. Our present investigation created positive impact especially on the local people who expressed their interest after learning the fact that there is sufficient scientific basis of the healing power of the plants. This will help in developing public awareness towards the conservation of the traditional knowledge as well as to preserve the plant diversity for the future generation. This is a necessity because a number of uses of plant species for medicinal purposes are unique to this study and may contribute to further research and development of novel drugs.

## References

[1] Chowdhury KAN (2007). Residence, Gender and Power in the Garo Society of Bangladesh. Doctoral Thesis.

[2] Burling R (1997). The Strong Women of Modhupur.

[3] Bleie T (2005). Tribal People, Nationalism and the Human Rights Challenges: the Adivasis of Bangladesh.

[4] Khan MA, Hasan MN, Jahan N, Das PR, Islam MT, Bhuiyan MSA (2012). Ethnomedicinal wisdom and famine food plants of the Hajong community of Baromari village in Netrakona district of Bangladesh. Am-Eur J Sustain Agr.

[5] Qureshi R, Bhatti GR (2004). Floristic and ethnobotanical study of Desert-Nara Region, Sindh. Shah Abdul Latif University, Pakistan Research Repository.

[6] Banglapedia, National Encyclopedia of Bangladesh. Dhaka, Bangladesh: Asiatic Society of Bangladesh; 2013.

[7] Alexiades MN (1996). Selected guidelines for ethnobotanical research: a field manual.

[8] Martin GJ (2004). Ethnobotany: A methods manual. Earthscan Publications Ltd.

[9] Cotton CM (1996). Ethnobotany: Principles and applications.

[10] Maundu P (1995). Methodology for collecting and sharing indigenous knowledge: a case study. Indigen Knowl Dev Monit.

[11] Phillips O, Gentry AH, Reynel C, Wilkin P, Galvez-Durand BC (1994). Quantitative ethnobotany and Amazonian conservation. Conserv Biol.

[12] Canales M, Hernandez T, Caballero J, Romo De Vivar A, Avila G, Duran A (2005). Informant consensus factor and antibacterial activity of the medicinal plants used by the people of San Rafael Coxcatlan, Puebla, Mexico. J Ethnopharmacol.

[13] Heinrich M, Ankli A, Frei B, Weimann C, Sticher O (1998). Medicinal plants in Mexico: healers’ consensus and cultural importance. Soc Sci Med.

[14] Chandra PK (2005). Ethnomedicinal botany of the Apatani in the Eastern Himalayan region of India. J Ethnobiol Ethnomed.

[15] Lulekal E, Kelbessa E, Bekele T, Yineger H (2008). An ethnobotanical study of medicinal plants in Mana Angetu District, southeastern Ethiopia. J Ethnobiol Ethnomed.

[16] Islam MK, Saha S, Mahmud I, Mohamad K, Awang K, Jamal Uddin S (2014). An ethnobotanical study of medicinal plants used by tribal and native people of Madhupur forest area, Bangladesh. J Ethnopharmacol.

[17] Pengelly A (2004). The constituents of medicinal plants.

[18] Daniel M (2006). Medicinal Plants: Chemistry and Properties.

[19] Rahmatullah M, Mukti IJ, Haque AKMF, Mollik MAH, Parvin K, Jahan R (2009). An Ethnobotanical Survey and Pharmacological Evaluation of Medicinal Plants used by the Garo Tribal Community living in Netrakona district, Bangladesh. Adv Nat Appl Sci.

[20] Akerreta S, Cavero RY, Calvo MI (2007). First comprehensive contribution to medical ethnobotany of Western Pyrenees. J Ethnobiol Ethnomed.

[21] Nanyingi MO, Mbaria JM, Lanyasunya AL, Wagate CG, Koros KB, Kaburia HF (2008). Ethnopharmacological survey of Samburu district. J Ethnobiol Ethnomed, Kenya.

[22] Murad W, Azizullah A, Adnan M, Tariq A, Khan KU, Waheed S (2013). Ethnobotanical assessment of plant resources of Banda Daud Shah, District Karak, Pakistan. J Ethnobiol Ethnomed.

[23] Muthu C, Ayyanar M, Raja N, Ignacimuthu S (2006). Medicinal plants used by traditional healers in Kancheepuram District of Tamil Nadu, India. J Ethnobiol Ethnomed.

[24] Balick M (1996). Cox P. Plants culture and people.

[25] Ghorbani A (2005). Studies in pharmaceutical ethnobotany in the region of Turkmen Sahra, North of Iran (part 1): general results. J Ethnopharmacol.

[26] Giday M, Asfaw Z, Woldu Z (2009). Medicinal plants of the Meinit ethnic group of Ethiopia: an ethnobotanical study. J Ethnopharmacol.

[27] Telefo PB, Lienou LL, Yemele MD, Lemfack MC, Mouokeu C, Goka CS (2011). Ethnopharmacological survey of plants used for the treatment of female infertility in Baham, Cameroon. J Ethnopharmacol.

[28] Song MJ, Kim H, Heldenbrand B, Jeon J, Lee S (2013). Ethnopharmacological survey of medicinal plants in Jeju Island, Korea. J Ethnobiol Ethnomed.

[29] Uniyal SK, Singh KN, Jamwal P, Lal B (2006). Traditional use of medicinal plants among the tribal communities Chhota, Western Himalaya. J Ethnobiol Ethnomed.

[30] Sanz-Biset J, Campos-de-la-Cruz J, Epiquién-Rivera MA, Cañigueral S (2009). A first survey on the medicinal plants of the Chazuta valley (Peruvian Amazon). J Ethnopharmacol.

[31] Hasan A, Shaown MSH, Ripa RJ, Khatun A, Khan MAA, Akter MS (2013). Ethnomedicinal plants of two villages in Natore district, Bangladesh. Am-Eur J Sustain Agr.

[32] Rahmatullah M, Islam T, Hasan ME, Ahmed R, Jamal F, Jahan R (2010). A survey of medicinal plants used by the folk medicinal practitioners of Shetabganj village in Dinajpur ditrict, Bangladesh. Am-Eus J Sustain Agr.

[33] Rahmatullah M, Rahman MA, Haque MZ, Mollik MAH, Miajee ZUMEU, Begum R (2010). A survey of medicinal plants used by folk medicinal practitioners of Station Purbo Para village of Jamalpur Sadar Upazila in Jamalpur district, Bangladesh. Am Eur J Sustain Agr.

[34] Malek I, Mia N, Mustary ME, Hossain MJ, Sathi SM, Parvez MJ (2014). Medicinal plants of the Chakma community of Rangapanir Chara Area of Khagrachaari district, Bangladesh. Am-Eur J Sustain Agr.

[35] Neves JM, Matos C, Moutinho C, Queiroz G, Gomes LR (2009). Ethnopharma-cological notes about ancient uses of medicinal plants in Tras-os-Montes (northern of Portugal). J Ethnopharmacol.

[36] Kadir MF, Bin Sayeed MS, Mia MM (2012). Ethnopharmacological survey of medicinal plants used by indigenous and tribal people in Rangamati, Bangladesh. J Ethnopharmacol.

[37] Teklehaymanot T, Giday M (2012). Ethnobotanical study of medicinal plants used by people in Zegie Peninsula, northwestern Ethiopia. J Ethnobiol Ethnomed.

[38] Hasan SA, Uddin MM, Huda KN, Das A, Tabassum N, Hossain MR (2014). Ethnomedicinal plants of two village folk medicinal practitioners in Rajshahi district, Bangladesh: Comparision of their folk medicinal uses with Ayurvedic uses. Am-Eur J Sustain Agr.

[39] Rahmatullah M, Islam MR, Kabir MZ, Harun-or-Rashid M, Jahan R, Begum R (2010). Folk medicinal practices in Vasu Bihar village, Bogra district, Bangladesh. Am-Eur J Sustain Agric.

[40] Biswas B, Arafat MY, Hossain MS, Sakib-uz-Zaman M, Khatun Z, Rahmatullah M (2014). Ethnomedicinal practices of a village folk medicinal practitioner in Faridpur district, Bangladesh. Am-Eur J Sustain Agr.

[41] Rahmatullah M, Khatun MA, Morshed N, Neogi PK, Khan SUA, Hossan MS (2010). A randomized survey of medicinal plants used by folk medicinal practitioners of Sylhet Division, Bangladesh. Adv Nat Appl Sci.

[42] Rahmatullah M, Nuruzzaman M, Hossan MS, Khatun MA, Rahman MM, Jamal F (2010). An ethnomedicinal survey of folk medicinal practitioners of Shitol Para village, Jhalokati district, Bangladesh. Adv Nat Appl Sci.

[43] Rahmatullah M, Mollik MAH, Jilani MA, Hossain MA, Hossain MS, Rahman MM (2010). Medicinal plants used by folk medicinal practitioners in three villages of Natore and Rajshahi districts, Bangladesh. Adv Nat Appl Sci.

[44] Rahmatullah M, Momen MA, Rahman MM, Nasrin D, Hossain MS, Khatun Z (2010). A randomized survey of medicinal plants used by folk medicinal practitioners in Daudkandi sub-district of Comilla district, Bangladesh. Adv Nat Appl Sci.

[45] Rahmatullah M, Noman A, Hossan MS, Harun-Or-Rashid M, Rahman T, Chowdhury MH (2009). A survey of medicinal plants in two areas of Dinajpur district, Bangladesh including plants which can be used as functional foods. Am-Eur J Sustain Agr.

[46] Mia MMK, Kadir MF, Hossan MS, Rahmatullah M (2009). Medicinal plants of the Garo tribe inhabiting the Madhupur forest region of Bangladesh. Am-Eur J Sustain Agr.

[47] Rahmatullah M, Mahmud AA, Rahman MA, Uddin MF, Hasan M, Khatun MA (2011). An ethnomedicinal survey conducted amongst folk medicinal practitioners in the two southern districts of Noakhali and Feni, Bangladesh. Am-Eur J Sustain Agr.

[48] Rahmatullah M, Mollik MAH, Harun-or-Rashid M, Tanzin R, Ghosh KC, Rahman H (2010). A comparative analysis of medicinal plants used by folk medicinal healers in villages adjoining the Ghaghot, Bangali and Padma Rivers of Bangladesh. Am-Eur J Sustain Agr.

[49] Rahmatullah M, Islam MR, Kabir MZ, Harun-or-Rashid M, Jahan R, Begum R (2010). Folk medicinal practices in Vasu Bihar village, Bogra district, Bangladesh. Am-Eur J Sustain Agr.

[50] Rahmatullah M, Hasan MM, Ahmed M, Khan MW, Hossan MS, Rahman MM (2010). A survey of medicinal plants used by folk medicinal practitioners in Balidha village of Jessore district, Bangladesh. Am-Eur J Sustain Agr.

[51] Rahmatullah M, Azam MNK, Mollik MAH, Hasan MM, Hassan AI, Jahan R (2010). Medicinal plants used by the Kavirajes of Daulatdia Ghat, Kushtia district, Bangladesh. Am-Eur J Sustain Agr.

[52] Rahmatullah M, Mollik MAH, Ali M, Abbas MFB, Jahan R, Chowdhury MH (2010). An ethnomedicinal survey of Vitbilia village in Sujanagar sub-district of Pabna district, Bangladesh. Am-Eur J Sustain Agr.

[53] Rahmatullah M, Hasan MR, Hossan MS, Jahan R, Chowdhury MH, Seraj S (2010). A survey of medicinal plants used by folk medicinal practitioners of six villages in Greater Naogaon district, Bangladesh. Am-Eur J Sustain Agr.

[54] Hasan MM, Annay MEA, Sintaha M, Khaleque HN, Noor FA, Nahar A (2010). A survey of medicinal plant usage by folk medicinal practitioners in seven villages of Ishwardi Upazilla, Pabna district, Bangladesh. Am-Eur J Sustain Agr.

[55] Karim MS, Rahman MM, Shahid SB, Malek I, Rahman MA, Jahan S (2011). Medicinal plants used by the folk medicinal practitioners of Bangladesh: a randomized survey in a village of Narayanganj district. Am-Eur J Sustain Agr.

[56] Haque MA, Shaha MK, Ahmed SU, Akter R, Rahman H, Chakravotry S (2011). Use of inorganic substances in folk medicinal formulations: a case study of a folk medicinal practitioner in Tangail district, Bangladesh. Am-Eur J Sustain Agr.

[57] Esha RT, Chowdhury MR, Adhikary S, Haque KMA, Acharjee M, Nurunnabi M (2012). Medicinal plants used by tribal medicinal practitioners of three clans of the Chakma tribe residing in Rangamati district, Bangladesh. Am-Eur J Sustain Agr.

[58] Das PR, Islam MT, Mahmud ASMSB, Kabir MH, Hasan ME, Khatun Z (2012). An ethnomedicinal survey aconducted among the folk medicinal practitioners of three villages in Kurigram district, Bangladesh. Am-Eur J Sustain Agr.

[59] Mou SM, Mahal MJ, Bhuiyan P, Zakaria ASM, Datta B, Rana M (2012). Medicinal plants and formulations of the Goala tribe of Moulvibazar, Bangladesh. Am-Eur J Sustain Agr.

[60] Hossain S, Mahmud S, Rahmatullah M (2012). Inter-country exchanges of folk medicinal practices: a case study of a folk medicinal practitioner of Savar in Dhaka district, Bangladesh. Am-Eur J Sustain Agr.

[61] Mawla F, Khatoon S, Rehana F, Jahan S, Shelley MMR, Hossain S (2012). Ethnomedicinal plants of folk medicinal practitioners in four villages of Natore and Rajshahi districts, Bangladesh. Am-Eur J Sustain Agr.

[62] Zahan T, Ahmed I, Omi SI, Naher K, Islam S, Mahmud ASMSHB (2013). Ethnomedicinal uses of medicinal plants by the Tudu sub-clan of the Santal tribe in Joypurhat district of Bangladesh. Am-Eur J Sustain Agr.

[63] Jahan N, Khan A, Hasan MN, Hossain MU, Das U, Sultana S (2013). Ethnomedicinal plants of fifteen clans of the Garo tribal community of Madhupur in Tangail district, Bangladesh. Am-Eur J Sustain Agr.

[64] Tuhin MIH, Asaduzzaman M, Islam E, Khatun Z, Rahmatullah M (2013). Medicinal plants used by folk medicinal herbalists in seven villages of Bhola district, Bangladesh. Am-Eur J Sustain Agr.

[65] Naher S, Ferdous B, Datta T, Rashid UF, Tasnim TN, Akter S (2013). Ayurvedic influences in folk medicine: a case study of a folk medicinal practitioner of Jhalokathi in Barisal district, Bangladesh. Am-Eur J Sustain Agr.

[66] Khatun A, Khan MAA, Rahman MA, Akter MS, Hasan A, Parvin W (2013). Ethnomedicinal usage of plants and animals by folk medicinal practitioners of three villages in Chuadanga and Jhenaidah districts, Bangladesh. Am-Eur J Sustain Agr.

[67] Azam MNK, Ahmed MN, Rahman MM, Rahmatullah M (2013). Ethnomedicines used by the Oraon and Gor tribes of Sylhet district, Bangladesh. Am-Eur J Sustain Agr.

[68] Nahar MN, Ferdous J, Samanta FZ, Shuly KA, Nahar S, Saha R (2013). Ethnomedicinal plants of the Rai clan of the Tipra tribe of Sylhet Division, Bangladesh. Am-Eur J Sustain Agr.

[69] Malek I, Miah MR, Khan MF, Awal RBF, Nahar N, Khan I (2014). Medicinal plants of two practitioners in two Marma tribal communities of Khagrachhari district, Bangladesh. Am-Eur J Sustain Agr.

[70] Hasan MMA, Musulli S, Asadujjaman, Rabbi MG, Salahuddin M, Abedin MM, et al. Ethnomedicinal wisdom of tribal and folk medicinal practitioners practicing among Khasia tribal communities in Jaflong, Sylhet district, Bangladesh. Am-Eur J Sustain Agr. 2014;8:69–77.

[71] Shahneowaj AHM, Al-Mamun M, Sultana T, Papri A, Akter MN, Ashiq AR (2014). Medicinal practices of a Pahan tribal healer in Dinajpur district, Bangladesh. Am-Eur J Sustain Agr.

[72] Hossain M, Sharif R, Mamun AH, Rayhana N, Begum K, Tripty F (2014). Phytomedeicines of traditional health-care professionals in the vicinity of Lawachara Forest Reserve, Moulvibazar district, Bangladesh. Am-Eur J Sustain Agr.

[73] Rahmatullah M, Khatun Z, Barua D, Alam MU, Jahan S, Jahan R (2013). Medicinal plants used by traditional practitioners of the Kole and Rai tribes of Bangladesh. J Alternat Complement Med.

[74] Rahmatullah M, Haque MR, Islam SK, Jamal F, Bashar ABMA, Ahmed R (2010). A survey on the use of medicinal plants by folk medicinal practitioners in three areas of Pirojpur district, Bangladesh. Am-Eur J Sustain Agr.

[75] Rahmatullah M, Hossan MS, Hanif A, Roy P, Jahan R, Khan M (2009). Ethnomedicinal applications of plants by the traditional healers of the Marma tribe of Naikhongchhari, Bandarban district, Bangladesh. Adv Nat Appl Sci.

[76] Hanif A, Hossan MS, Mia MMK, Islam MJ, Jahan R, Rahmatullah M (2009). Ethnobotanical survey of the Rakhain tribe inhabiting the Chittagong Hill Tracts Region of Bangladesh. Am-Eur J Sustain Agr.

[77] Shahidullah M, Al-Mujahidee M, Uddin SMN, Hossan MS, Hanif A, Bari S (2009). Medicinal plants of the Santal tribe residing in Rajshahi district, Bangladesh. Am-Eur J Sustain Agr.

[78] Hossan MS, Hanif A, Khan M, Bari S, Jahan R, Rahmatullah M (2009). Ethnobotanical survey of the Tripura tribe of Bangladesh. Am-Eur J Sustain Agr.

[79] Rahman MA, Islam S, Naim N, Chowdhury MH, Jahan R, Rahmatullah M (2010). Use of medicinal plants by folk medicinal practitioners among a heterogenous population of Santals and non-Santals in two villages of Rangpur district, Bangladesh. Am-Eur J Sustain Agr.

[80] Rahmatullah M, Mollik MAH, Islam MK, Islam MR, Jahan FI, Khatun Z (2010). A survey of medicinal and functional food plants used by the folk medicinal practitioners of three villages in Sreepur Upazilla, Magura district, Bangladesh. Am-Eur J Sustain Agr.

[81] Rahmatullah M, Mollik MAH, Ahmed MN, Bhuiyan MZA, Hossain MM, Azam MNK (2010). A survey of medicinal plants used by folk medicinal practitioners in two villages of Tangail district, Bangladesh. Am-Eur J Sustain Agr.

[82] Rahmatullah M, Das PR, Islam T, Ripa RJ, Hasan E, Akter S (2012). Medicinal plants and formulations of the Bongshi tribe of Bangladesh. Am-Eur J Sustain Agr.

[83] Mukti M, Ahmed A, Chowdhury S, Khatun Z, Bhuiyan P, Debnath K (2012). Medicinal plant formulations of Kavirajes in several areas of Faridpur and Rajbari districts, Bangladesh. Am-Eur J Sustain Agr.

[84] Mou SM, Mahal MJ, Bhuiyan P, Zakaria ASM, Datta B, Rana M (2012). Medicinal plants and formulations of small tribes of Bangladesh: a case study of the Naik clan of the Rajbongshi tribe. Am-Eur J Sustain Agr.

[85] Disha IT, Khatun Z, Rahmatullah M (2012). Incantations, medicinal plants and formulations of the Rai Kshatriya tribe of Pabna district, Bangladesh. Am-Eur J Sustain Agr.

[86] Sarker MN, Mahin AA, Munira S, Akter S, Parvin S, Malek I (2013). Ethnomedicinal plants of the Pankho community of Bilaichari Union in Rangamati district, Bangladesh. Am-Eur J Sustain Agr.

[87] Hasan ME, Piya NS, Chowdhury HR, Sarker ML, Azad TT, Roney MSI (2013). Medicinal plants and formulations of the Murmu tribal community residing in Rajshahi district of Bangladesh. Am-Eur J Sustain Agr.

[88] Walid R, Suvro KFA, Harun-or-Rashid M, Mukti M, Rahman S, Rahmatullah M (2013). Ethnomedicinal plants of folk medicinal practitioners of two villages in Bagerhat district of Bangladesh. Am-Eur J Sustain Agr.

[89] Mukti M, Rahman MA, Bashar ABMA, Hossain S, Rahmatullah M (2013). Medicinal plants of the Khatriya and Kashya clans of the Bagdi people of Rajbari district in Bangladesh. Am-Eur J Sustain Agr.

[90] Goswami H, Hassan MR, Rahman H, Islam E, Asaduzzaman M, Prottoy MA (2013). Ethnomedicinal wisdom of the Tripura tribe of Comilla district, Bangladesh: a combination of medicinal plant knowledge and folk beliefs. Am-Eur J Sustain Agr.

[91] Wahab A, Roy S, Habib A, Bhuiyan MRA, Roy P, Khan MGS (2013). Ethnomedicinal wisdom of a Tonchongya tribal healer practicing in Rangamati district, Bangladesh. Am-Eur J Sustain Agr.

[92] Kabir MH, Hasan N, Rahman MM, Rahman MA, Khan JA, Hoque NT (2013). Tribal medicine in tribes who have lost their identities: Medicinal plants of tea garden workers in Sreemangal, Maulvibazar district, Bangladesh. Am-Eur J Sustain Agr.

[93] Rifat MRH, Prottoy MA, Arabi MAHS, Sultana R, Chakrabortty S, Eva K (2014). Blending of indigenous medicinal practices: A case of Chakma, Garo and Kush tribal practitioners practicing among Garo and Kush tribes in Sherpur district, Bangladesh. Am-Eur J Sustain Agr.

[94] Eva SA, Mouri TI, Sheela SA, Noshine M, Sultana F, Rahman T (2014). Folkloric knowledge of medicinal plants: an account of eight folk herbalists in Comilla district, Bangladesh. Am-Eur J Sustain Agr.

[95] Rahmatullah M, Rahman MA, Hossan MS, Taufiq-Ur-Rahman M, Jahan R, Mollik MAH (2010). A pharmacological and phytochemical evaluation of medicinal plants used by the Harbang clan of the Tripura tribal community of Mirsharai Area, Chittagong district, Bangladesh. J Alternat Complement Med.

[96] Das PR, Islam MT, Jahan R, Rahmatullah M (2013). Ethnomedicinal plants used by the Nag clan of the Rai Ghatual tribe of Moulvibazar district, Bangladesh. Ancient Sci Life.

[97] Rahmatullah M, Das AK, Mollik MAH, Jahan R, Khan M, Rahman T (2009). An ethnomedicinal survey of Dhamrai sub-district in Dhaka district, Bangladesh. Am-Eur J Sustain Agr.

[98] Chowdhury AR, Jahan FI, Seraj S, Khatun Z, Jamal F, Ahsan S (2010). A survey of medicinal plants used by Kavirajes of Barisal Town in Barisal district, Bangladesh. Am-Eur J Sustain Agr.

[99] Hossan MS, Roy P, Seraj S, Mou SM, Monalisa MN, Jahan S (2012). Ethnomedicinal knowledge among the Tonchongya tribal community of Roangchaari Upazila of Bandarban district, Bangladesh. Am-Eur J Sustain Agr.

[100] Aiubali, Rahman MM, Hossan MY, Aziz N, Mostafa MN, Mahmud MS, et al. Home remedies of the Teli clan of the Telegu tribe of Maulvibazar district, Bangladesh. Am-Eur J Sustain Agr. 2013;7:290–4.

[101] Hasan GM, Hossain D, Ahmed M, Jui NN, Mia MF, Malek I (2014). Traditional phytotherapy among the Manipuri tribe in Kamalganj Upazila of Moulvibazar district, Bangladesh. Am-Eur J Sustain Agr.

[102] Das PR, Islam MT, Mostafa MN, Rahmatullah M (2013). Ethnomedicinal plants of the Bauri tribal community of Moulvibazar district, Bangladesh. Ancient Sci Life.

[103] Kabir MH, Hasan N, Rahman MM, Rahman MA, Khan JA, Hoque NT (2014). A survey of medicinal plants used by the Deb barma clan of the Tripura tribe of Moulvibazar district, Bangladesh. J Ethnobiol Ethnomed.

[104] Hasan ME, Akter S, Piya NS, Nath PK, Nova USR, Chowdhury HR (2012). Variations in selection of medicinal plants by tribal healers of the Soren clan of the Santal tribe: a study of the Santals in Rajshahi district, Bangladesh. Am-Eur J Sustain Agr.

[105] Rana MS, Islam MM, Bosunia SN, Mahmud SR, Santa SA, Snigdha SH (2014). A survey of medicinal plants used by a village folk medicinal practitioner in Sreemangal Upazila of Maulvibazar district, Bangladesh. Am-Eur J Sustain Agr.

[106] Jibon RI, Tusher SM, Sabuj MH, Islam MS, Hossain MS, Paul AK (2014). Medicinal plants of a folk medicinal practitioner of Jamalpur district, Bangladesh and comparative ethnomedicinal usages of some plants. Am-Eur J Sustain Agr.

[107] Rahmatullah M, Mollik AH, Rahman S, Hasan N, Agarwala B, Jahan R (2010). A medicinal plant study of the Santal tribe in Rangpur district, Bangladesh. J Alternat Complement Med.

[108] Rahmatullah M, Kabir AABT, Rahman MM, Hossan MS, Khatun Z, Khatun MA (2010). Ethnomedicinal practices among a minority group of Christians residing in Mirzapur village of Dinajpur district, Bangladesh. Adv Nat Appl Sci.

[109] Rahmatullah M, Mollik MAH, Khatun MA, Jahan R, Chowdhury AR, Seraj S (2010). A survey on the use of medicinal plants by folk medicinal practitioners in five villages of Boalia sub-district, Rajshahi district, Bangladesh. Adv Nat Appl Sci.

[110] Rahmatullah M, Ferdausi D, Mollik MAH, Azam MNK, Taufiq-Ur-Rahman M, Jahan R (2009). Ethnomedicinal survey of Bheramara area in Kushtia district, Bangladesh. Am-Eur J Sustain Agr.

[111] Rahmatullah M, Mollik MAH, Azam ATMA, Islam MR, Chowdhury MAM, Jahan R (2009). Ethnobotanical survey of the Santal tribe residing in Thakurgaon district, Bangladesh. Am-Eur J Sustain Agr.

[112] Rahmatullah M, Haque ME, Mondol MRK, Mandal A, Azad MAZ, Seraj S (2011). Medicinal plants of the Hodis: a disappearing tribe of Bangladesh. J Alternat Complement Med.

[113] Rahmatullah M, Mollik MAH, Paul AK, Jahan R, Khatun MA, Seraj S (2010). A comparative analysis of medicinal plants used to treat gastrointestinal disorders in two sub-districts of Greater Khulna Division, Bangladesh. Adv Nat Appl Sci.

[114] Rashid MM, Rafique FB, Debnath N, Rahman A, Zerin SZ, Harun-ar-Rashid, et al. Medicinal plants and formulations of a community of the Tonchongya tribe in Bandarban district of Bangladesh. Am-Eur J Sustain Agr. 2012;6:292–8.

[115] Sarker B, Akther F, Ayman U, Sifa R, Jahan I, Sarker M (2012). Ethnomedicinal investigations among the Sigibe clan of the Khumi tribe of Thanchi sub-district of Bandarban district, Bangladesh. Am-Eur J Sustain Agr.

[116] Rahmatullah M, Biswas KR (2012). Traditional medicinal practices of a Sardar healer of the Sardar (Dhangor) community of Bangladesh. J Alternat Complement Med.

[117] Rahmatullah M, Pk SR, Al-Imran M, Jahan R (2013). The Khasia tribe of Sylhet district, Bangladesh, and their fast-disappearing knowledge of medicinal plants. J Alternat Complement Med.

[118] Haque MM, Choudhury MS, Hossain MS, Haque MA, Seraj S, Rahmatullah M (2012). Ethnographic information and medicinal formulation of a Mro community of Gazalia Union in the Bandarbans district of Bangladesh. Am-Eur J Sustain Agr.

[119] Moonmoon M, Islam SA, Bristy STJ, Kader MB, Akhter S, Kumar PKS (2014). Medicinal plant knowledge of a folk medicinal practitioner in Aria Bazar village, Bogra district, Bangladesh. Am-Eur J Sustain Agr.

[120] Mollik MAH, Hassan AI, Paul TK, Sintaha M, Khaleque HN, Noor FA (2010). A survey of medicinal plant usage by folk medicinal practitioners in two villages by the Rupsha River in Bagerhat district, Bangladesh. Am-Eur J Sustain Agr.

